# Stereoselective Palladium-Catalyzed Hiyama Cross-Coupling Reaction of Tetrasubstituted *gem*-Difluoroalkenes

**DOI:** 10.1021/acs.orglett.3c04037

**Published:** 2023-12-28

**Authors:** Min Li, Gavin Chit Tsui

**Affiliations:** Department of Chemistry, The Chinese University of Hong Kong, Shatin, New Territories, Hong Kong SAR 999077, China

## Abstract

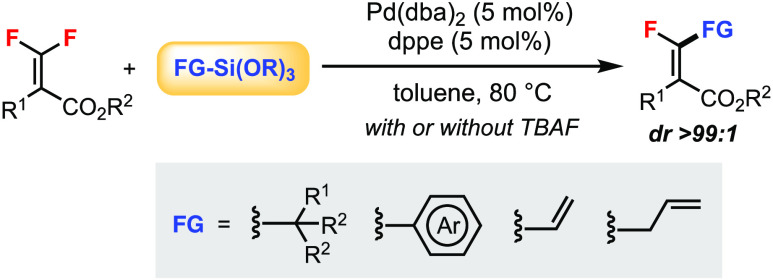

We herein describe a diastereoselective Pd(0)-catalyzed Hiyama cross-coupling reaction of *gem*-difluoroalkenes. The use of organosilicon reagents in this reaction is advantageous over other organometallic reagents by allowing the introduction of a wide range of functional groups, including challenging alkyl groups. Also conveniently, the additive TBAF was not required for (hetero)aryl-substituted difluoroalkenes.

Since its seminal report in 1988,^1^ the Hiyama cross-coupling reaction of organic halides with *organosilicon* reagents has become an indispensable tool for palladium-catalyzed C–C bond formation (Scheme 1a).^2^ Compared to other cross-coupling protocols, the use of organosilicon reagents is attractive due to their stability and low toxicity. A wide range of organosilicon compounds are also commercially available and inexpensive because of the natural abundance of silicon. A nucleophilic activator such as fluoride is commonly employed in these reactions, as the C–Si bonds are less polarized and relatively inert. Despite the significant progress, the Hiyama cross-coupling of organic *fluorides* through C–F bond activation has remained a major obstacle.

**Scheme 1:**
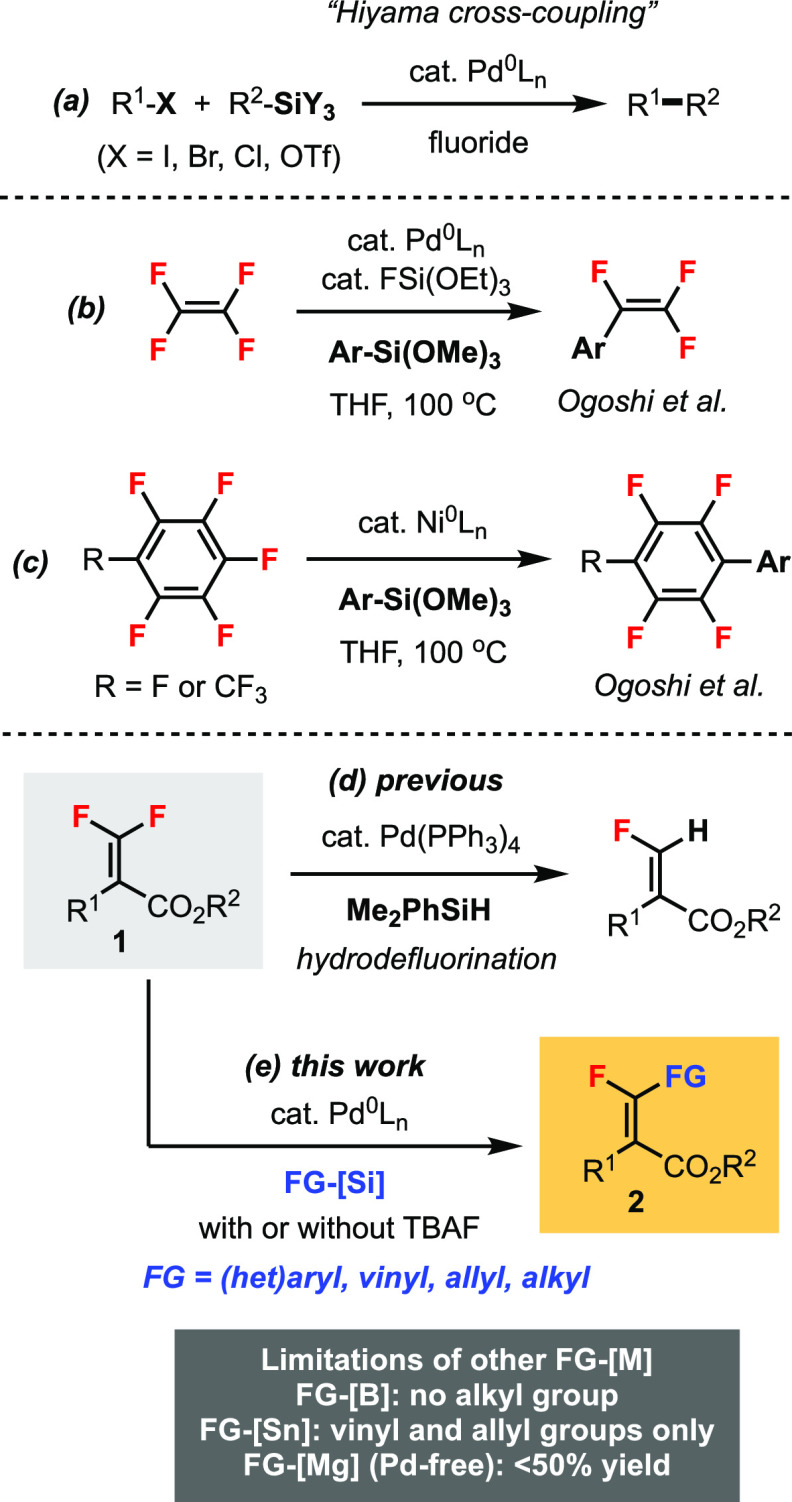
Hiyama Cross-Coupling Reaction of *gem*-Difluoroalkenes

The pioneering work from Ogoshi’s group^3^ demonstrated that by using perfluorinated compounds such as tetrafluoroethylene (TFE), hexafluorobenzene, or octafluorotoluene, the Hiyama coupling of a C–F bond could be achieved using arylsiloxanes under Pd or Ni catalysis (Scheme 1b,c).^4^ On the other hand, the Hiyama cross-coupling of readily available *gem*-difluoroalkenes for the synthesis of valuable *monofluoroalkenes* was unknown.^5^ We have a continuing interest in the stereoselective Pd-catalyzed C–F bond functionalization of tetrasubstituted *gem*-difluoroalkenes **1**.^6^ In terms of Si-based reagents, we have reported the *hydrodefluorination* of **1** using hydrosilane Me_2_PhSiH (Scheme 1d).^6c^ In this work, a novel Hiyama cross-coupling reaction of **1** for the stereoselective synthesis of tetrasubstituted monofluoroalkenes **2** is described (Scheme 1e). Compared to previous protocols using other organometallic reagents FG–[M], this method significantly enhanced the functional group (FG) tolerability due to the use of organosilicon reagents. For instance, the Suzuki–Miyaura coupling of **1** using boronic acids (mainly aryl) did not allow the installation of alkyl groups.^6b^ The Stille coupling was specialized for vinylation and allylation of **1** with the disadvantage of using toxic organotin compounds.^7^ In the Pd-free C–F bond functionalization using Grignard reagents, the reaction scope was broader, but the maximum yield was not more than 50% due to the resolution nature of the reaction.^7b^

We began the optimization studies by using the tetrasubstituted *gem*-difluoroalkene **1a** (β,β-difluoroacrylate) as a standard substrate and triethoxyphenylsilane (3.0 equiv) as the reagent for generating the monofluoroalkene product **2a** (Table 1).^8^ In previous Suzuki–Miyaura coupling of **1a** with PhB(OH)_2_, the catalyst Pd(PPh_3_)_4_ was highly effective.^6b^ However, this catalyst only gave low yields in the Hiyama coupling (entries 1 and 2). Similar trends were observed for the Pd_2_(dba)_3_/dppe catalyst, which was effective for the Stille coupling of **1a** with vinyl-/allyl-SnBu_3_ (entries 3 and 4).^7a^ On the other hand, the *Pd(dba)*_*2*_*/dppe* catalyst gave 42% yield (entry 5). A dramatic increase in yield was observed by adding TBAF (2.0 equiv) to the reaction mixture (entry 6). Furthermore, the Pd catalyst loading could be lowered to 5 mol %, offering **2a** in 96% isolated yield as the *E* isomer with dr > 99:1 (entry 7). Reducing the organosilicon reagent to 1.5 equiv decreased the yield (entry 8). Other ligands with different carbon chains were screened for comparison, including dppm, dppp, and dppb, and all showed poorer reactivities than dppe (entries 9–11). Using Pd(dba)_2_ alone without dppe was ineffective (entry 12). Other fluoride additives were also screened, including KF, CsF, and AgF, and yields were not as good as with TBAF (entries 13–15). In the solvent screening, 1,4-dioxane and DMF were inferior to toluene for this reaction (entries 16 and 17). In all cases, only the *E* isomer of **2a** was detected.

**Table 1:**
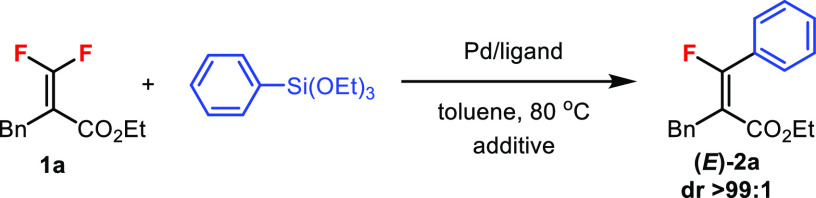
Optimization Studies^a^

entry	Pd (mol %)	ligand (mol %)	additive (equiv)	yield (%)^b^
1	Pd(PPh_3_)_4_ (10)	none	none	49
2	Pd(PPh_3_)_4_ (10)	none	TBAF (2.0)	0
3	Pd_2_(dba)_3_ (5)	dppe (10)	none	0
4	Pd_2_(dba)_3_ (5)	dppe (10)	TBAF (2.0)	34
5	Pd(dba)_2_ (10)	dppe (10)	none	42
6	Pd(dba)_2_ (10)	dppe (10)	TBAF (2.0)	99
**7**	**Pd(dba)**_**2**_ **(5)**	**dppe (5)**	**TBAF** **(2.0)**	**97** **(96)**^c^
8^d^	Pd(dba)_2_ (5)	dppe (5)	TBAF (2.0)	74
9	Pd(dba)_2_ (5)	dppm (5)	TBAF (2.0)	56
10	Pd(dba)_2_ (5)	dppp (5)	TBAF (2.0)	0
11	Pd(dba)_2_ (5)	dppb (5)	TBAF (2.0)	0
12	Pd(dba)_2_ (5)	none	TBAF (2.0)	0
13	Pd(dba)_2_ (5)	dppe (5)	KF (2.0)	54
14	Pd(dba)_2_ (5)	dppe (5)	CsF (2.0)	35
15	Pd(dba)_2_ (5)	dppe (5)	AgF (2.0)	0
16^e^	Pd(dba)_2_ (5)	dppe (5)	TBAF (2.0)	50
17^f^	Pd(dba)_2_ (5)	dppe (5)	TBAF (2.0)	12

a Unless specified otherwise, reactions were carried out using **1a** (0.1 mmol) and triethoxyphenylsilane (0.3 mmol) in toluene (0.2 M) for 12 h under argon.

b Yields were determined by ^19^F NMR analysis using benzotrifluoride as the internal standard. The diastereomeric ratios (dr) were determined by ^19^F NMR analysis.

c Isolated yield using 0.2 mmol of **1a**.

d Using 0.15 mmol of triethoxyphenylsilane.

e Using 1,4-dioxane as the solvent (0.2 M).

f Using DMF as the solvent (0.2 M).

The optimized Hiyama coupling conditions were applicable to the C–F bond functionalization of various tetrasubstituted *gem*-difluoroalkenes **1** (Scheme 2). The reaction at the 1.0 mmol scale was demonstrated to provide **2a** in 79% yield. Besides the phenyl group, vinyl and allyl groups could also be installed (**2b**, **2c**). More importantly, this method allowed the introduction of *alkyl* groups, including linear and branched carbon chains, in good yields (**2d**–**g**, 70–84% yield), which could not be achieved by previously developed protocols.^6,7^ The vinylic substituent group R^1^ could be varied, tolerating different alkyl and benzyl groups (**2h**–**m**). The ester substituent group R^2^ could also be changed to a bulkier *tert*-butyl group and did not affect the reaction (**2n**). All these products (*E*)-**2** were obtained with excellent dr (>99:1).

**Scheme 2:**
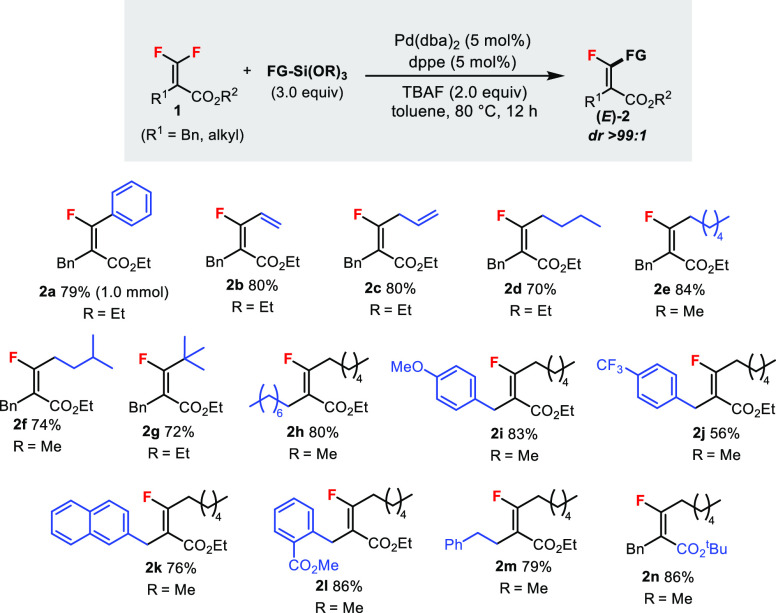
Hiyama Cross-Coupling Reaction of **1** Containing Benzyl or Alkyl Substituent Groups Unless specified otherwise, reactions were carried out using **1** (0.2 mmol) in toluene (1.0 mL) under argon. Isolated yields are reported. The diastereomeric ratios were determined by ^19^F NMR analysis.

The aryl-substituted *gem*-difluoroalkenes **1** are intrinsically more reactive than the alkyl-substituted counterparts.^6c^ In the Hiyama cross-coupling reaction, the phenyl substrate gave monofluorostilbene product **3a** in an excellent yield of 91% even *without* the addition of TBAF (Scheme 3). Similarly, vinyl (**3b**), allyl (**3c**), and primary (**3d**–**f**)/secondary (**3g**, **3h**)/tertiary (**3i**) alkyl groups could also be installed. Basic amine groups were compatible, albeit in lower yields (**3j**, **3k**). Substrates containing different aromatic (**3l**–**p**) and ester (**3q**, **3r**) substituent groups were tolerated. A heteroaryl group (thienyl) was shown to be compatible (**3s**). In all cases, only the *E* products were obtained (dr > 99:1).

**Scheme 3:**
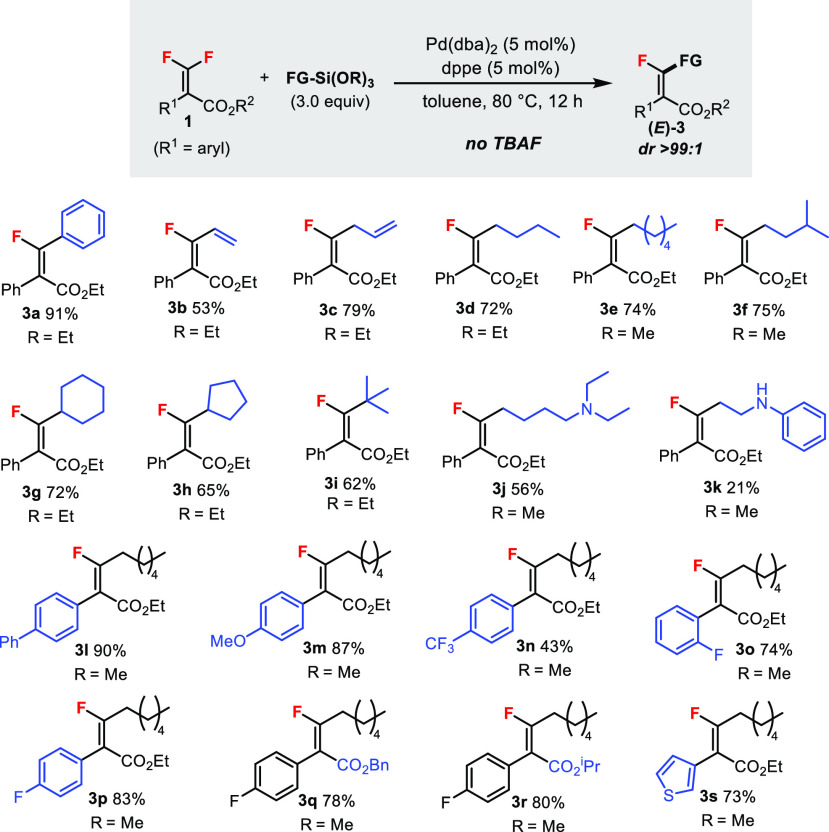
Hiyama Cross-Coupling Reaction of **1** Containing (Hetero)aryl Substituent Groups Unless specified otherwise, reactions were carried out using **1** (0.2 mmol) in toluene (1.0 mL) under argon. Isolated yields are reported. The diastereomeric ratios were determined by ^19^F NMR analysis.

Drug molecule modification involving the C–F bond Hiyama cross-coupling was explored (Scheme 4). Isoxepac (**4**) is a nonsteroidal anti-inflammatory agent (NSAID). It was converted to α-diazo ester **5** in two steps. The *gem*-difluoroalkene **6** could be obtained from **5**. The key Pd-catalyzed Hiyama reaction using an organosilicon reagent enabled the installation of the *n*-hexyl group diastereoselectively in product **7**. Overall, the monofluoroalkene motif was successfully introduced to isoxepac in a short sequence. This approach would be useful for drug discovery since monofluoroalkenes have been identified as peptide bond isosteres for pharmaceutical development.^5^

**Scheme 4:**
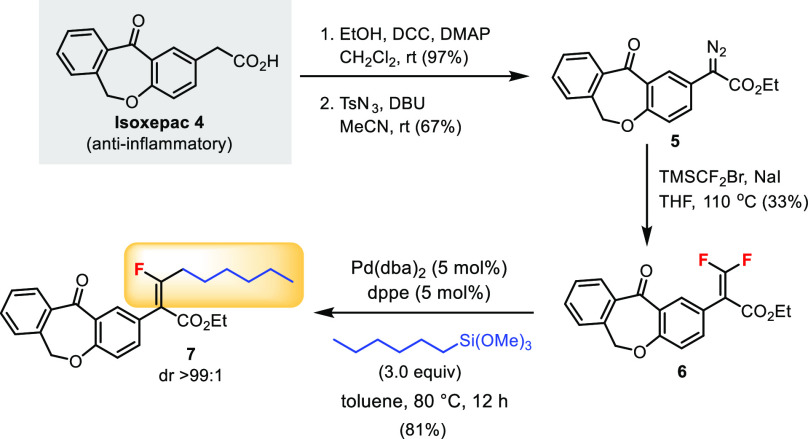
Modification of Drug Molecule Isoxepac

The reaction was not limited to tetrasubstituted β,β-difluoroacrylates. The *gem*-difluoroalkene **8** containing an *amide* moiety also afforded the C–F bond-coupled product (*E*)-**9** with excellent diastereoselectivity (eq 1). Moreover, *trisubstituted* difluoroacrylate **10** smoothly provided the trisubstituted monofluoroalkene product (*E*)-**11** (eq 2).
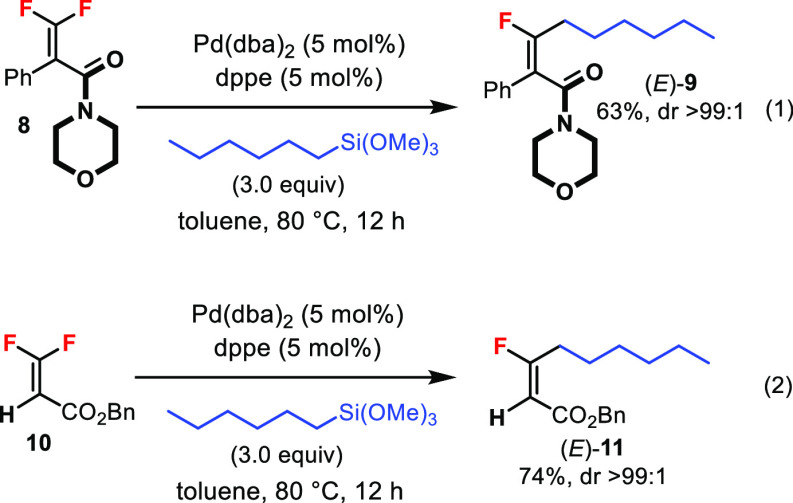
1

Control experiments were conducted to gain more insight into the reaction (Scheme 5). The trisubstituted *gem*-difluoroalkene **12** derived from aldehyde was not reactive under the standard conditions, highlighting the importance of the ester group in **1** for activation (Scheme 5a). Different organosilicon reagents were compared in the vinylation of **1h** (Scheme 5b), and the siloxanes were markedly more reactive than trichlorovinylsilane and triphenylvinylsilane. For the Hiyama cross-coupling of aryl-substituted **1**, no TBAF was needed for the reaction, which was more convenient and ensured functional group tolerability (cf. Scheme 3). In fact, in the allylation reaction of **1h**, adding TBAF decreased the yield of the desired product **3c** due to the generation of isomeric side product **3c′** (Scheme 5c). The isomerization of **3c** (1,4-diene) could be triggered by simply adding TBAF and heat to provide **3c′** (1,3-diene) in good yield (Scheme 5d).^9^

**Scheme 5:**
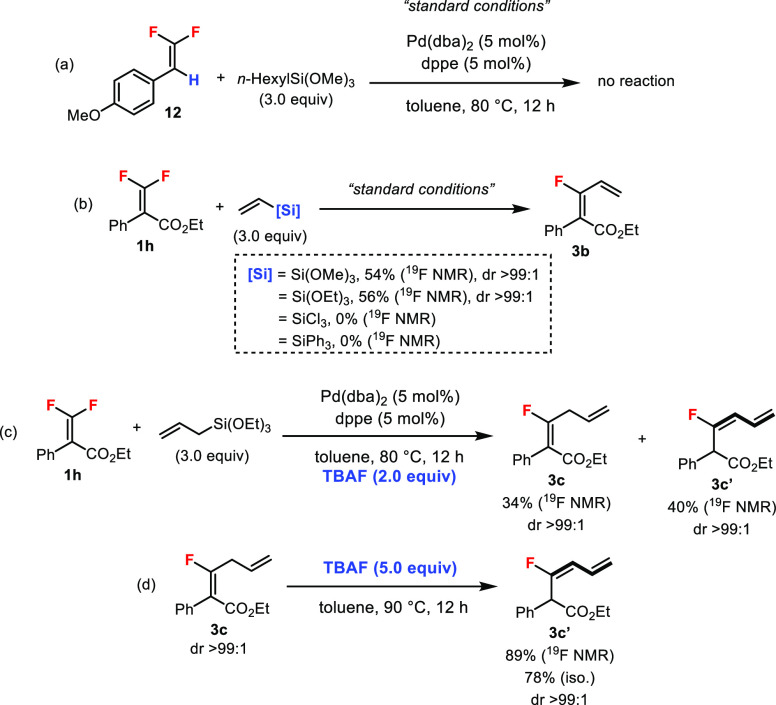
Control Experiments

In conclusion, we have developed a stereoselective C–F bond functionalization utilizing the Hiyama cross-coupling reaction between tetrasubstituted *gem*-difluoroalkenes and organosiloxanes. The reaction enables the installation of various functional groups, including challenging alkyl groups, in the (*E*)-monofluoroalkene products. This protocol significantly overcomes the scope limitations of previous coupling methods with other organometallic reagents.

## Data Availability

The data underlying this study are available in the published article and its Supporting Information

## Supporting Information Available

Experimental procedures, optimization data, characterization data, and spectral data (PDF)
