# Supplementary material for: Stereoselective Palladium-Catalyzed Hiyama Cross-Coupling Reaction of Tetrasubstituted gem-Difluoroalkenes
Source: Org Lett. 2023 Dec 28;26(1):376–9. doi: 10.1021/acs.orglett.3c04037 (PMC10789091; doi:10.1021/acs.orglett.3c04037)

Supporting Information

Stereoselective Palladium-Catalyzed Hiyama Cross-Coupling Reaction of Tetrasubstituted *gem*-Difluoroalkenes

*Min Li and Gavin Chit Tsui**

* Department of Chemistry, The Chinese University of Hong Kong, Shatin, New Territories, Hong Kong SAR
E-mail: gctsui@cuhk.edu.hk

Experimental Procedures and Spectral Data

<u>Table of Contents:</u>

I.	General Experimental	**S2**
II.	Materials	**S2**
III.	Instrumentation	**S2**
IV.	Experimental Procedures	**S5**
V.	Optimization Studies	**S7**
VI.	Derivatizations	**S8**
VII.	Characterization Data	**S9**
VIII.	Reference	**S19**
IX.	Spectra	**S20**

I. General Experimental.

Unless otherwise noted, cross-coupling reactions were carried out under argon in a 10 mL glass tube with magnetic stirring. Reactions that require heating were carried out in the oil bath. Analytical thin layer chromatography (TLC) was performed with Merck silica gel 60 F_{254} aluminum plates. Visualization was done under a UV lamp (254 nm) and by immersion in potassium permanganate ($KMnO_4$), followed by heating using a heat gun. Organic solutions were concentrated by rotary evaporation at 23-35 °C. Purification of reaction products were generally done by flash column chromatography with Silicycle 60-230 mesh silica gel.

II. Materials.

Anhydrous NaI, and $TMSCF_3$ were purchased from J&K Scientific. $Pd_2(dba)_3$, triethoxyphenylsilane, triethoxyvinylsilane and triethoxyallylsilane were purchased from Dikemann. $Pd(dba)_2$ and dppe were purchased from Acros. Diazo compounds for substrates synthesis were prepared according to literature procedure. Other chemicals for substrates preparation were purchased from Acros, J&K Scientific, Aldrich and Dikemann.

III. Instrumentation.

Proton nuclear magnetic resonance spectra (^{1}H NMR), carbon nuclear magnetic resonance spectra (^{13}C NMR) and fluorine nuclear magnetic resonance spectra (^{19}F NMR) were recorded at 23 °C on Bruker 400 MHz or 500 MHz spectrometer in $CDCl_3$. Chemical shifts of ^{1}H NMR spectra were reported as parts per million in δ scale using residual solvent signal ($CDCl_3$: 7.26 ppm) or tetramethylsilane (0.00 ppm) as internal standard. Chemical shifts of ^{13}C NMR spectra were reported using residual solvent signal of $CDCl_3$ (77.16 ppm) on the δ scale. Chemical shifts of ^{19}F NMR were reported as parts per million in δ scale using benzotrifluoride (-63.72 ppm) as internal standard. Data are represented as follows: chemical shift (δ ppm), multiplicity (s = singlet, d = doublet, t = triplet, q = quartet, m = multiplet), coupling constant (*J*, Hz) and integration. GC-MS analysis results were obtained on a Shimadzu GCMSQP2010 SE GC-MS Spectrometer. High resolution mass spectra (HRMS) were obtained on a Finnigan MAT 95XL GC Mass Spectrometer or a Thermo Scientific Q Exactive Focus Mass Spectrometer or a Bruker SolariX 9.4T FTMS.

Substrates 1 (tetrasubstituted *gem*-difluoroalkenes)

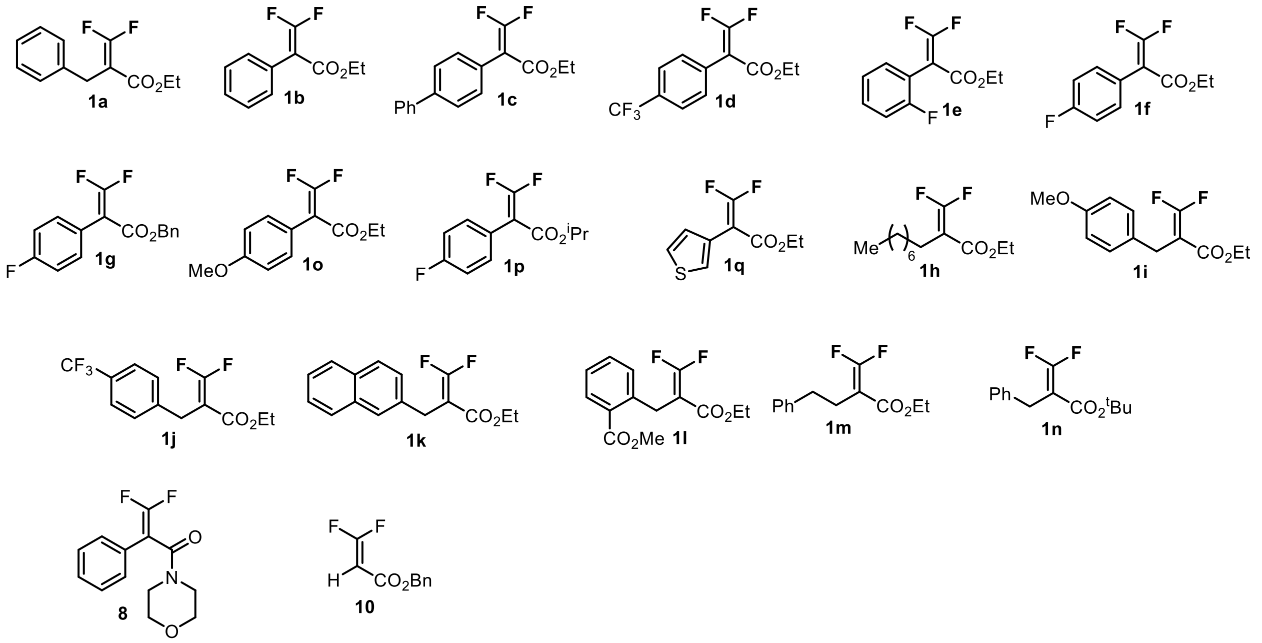

Note: Note: Substrates **1** are known compounds and prepared according to the literature procedure.[1, 2] Substrate **8** was prepared according to the literature procedure.[2] Substrate **10** was prepared according to the literature procedure.[6]

Substrates 2 (organosilicon reagents)

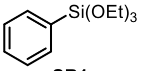

SR1

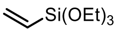

SR2

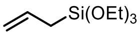

SR3

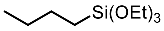

SR4

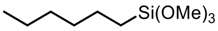

SR5

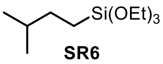

SR6

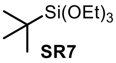

SR7

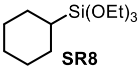

SR8

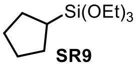

SR9

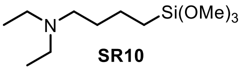

SR10

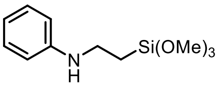

SR11

Note: Note: Substrates **SR7, SR8** and **SR9** are known compounds and prepared according to the literature procedure.[3]

IV. Experimental Procedures.

General procedure (I) for the synthesis of *gem*-difluoroalkenes 1 (using 1a as an example):

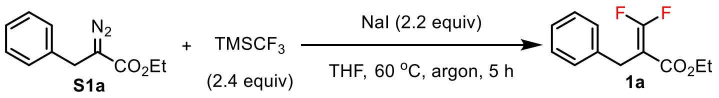

gem-Difluoroalkenes **1** were synthesized according to literature procedure.[1] For example, to an oven-dried 100 mL flask equipped with a stir bar was added dry NaI (989.0 mg, 6.6 mmol), followed by the addition of **S1a** (612.2 mg, 3.0 mmol) and $TMSCF_3$ (1.0238 g, 7.2 mmol) in 60 mL anhydrous THF under argon. The resulting mixture was heated at 60 °C with an oil bath for 5 h with rigorous stirring. After cooling to room temperature, the reaction mixture was extracted with 200 mL CH_2Cl_2, washed with H_2O (80 mL) then brine (80 mL), dried over $MgSO_4$ and concentrated in vacuo. The residue was purified by flash column chromatography on silica gel to afford *gem*-difluoroalkene **1a** as a colorless oil (472.0 mg, 67% yield).

Synthesis of gem-difluoroacrylate 6:

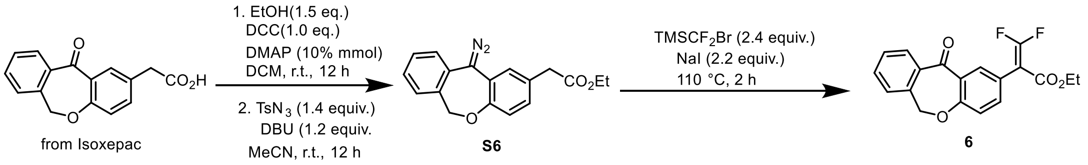

To a solution of Isoxepac (2.68g, 10.0 mmol) in dichloromethane (10.0 mL) was added EtOH(30 mmol, 1.38g), followed by DCC (11 mmol, 2.27 g) and DMAP (1 mmol, 122.2 mg). The mixture was stirred at roomtemperature for 12 h. The reaction mixture was extracted with CH_2Cl_2(3 × 50mL). The combined organic layers were washed with H_2O (2 × 50 mL), then brine (2 × 50 mL), dried over $MgSO_4$ and concentrated in vacuo. Dissolve the product in 60 mL MeCN, followed by TsN_3 (14 mmol, 2.76 g) and DBU (12 mmol, 1.82 g). The mixture was stirred at roomtemperature for 12 h. The reaction mixture was extracted with CH_2Cl_2 (3 × 50mL). The combined organic layers were washed with H_2O (2 × 50 mL), then brine (2 × 50 mL), dried over $MgSO_4$ and concentrated in vacuo. The residue was purified by flash column chromatography on silica gel (EA: hexane = 1 : 10) to afford products **S6** (2.06 g, 67% yield, colourless solid, R_f = 0.38 (DCM: hexane = 1 : 10).

To an oven-dried 100 mL flask equipped with a stir bar was added dry NaI (989.0 mg, 6.6 mmol), followed by the addition of **S6** (1.85 g, 6.0 mmol) and $TMSCF_3$ (2.05 g, 14.4 mmol) in 120 mL anhydrous THF under argon. The resulting mixture was heated at 60 °C with an oil bath for 6 h with rigorous stirring. After cooling to room temperature, the reaction mixture was extracted with 200 mL CH_2Cl_2, washed with H_2O (80 mL) then brine (80 mL), dried over $MgSO_4$ and concentrated in vacuo. The residue was purified by flash column chromatography on silica gel to afford *gem*-difluoroalkene **6** as a colorless oil (691.1 mg, 33% yield, colourless oil, R_f = 0.22 (DCM : hexane = 1 : 5)). **^{1}H NMR** (500 MHz, $CDCl_3$): δ 8.20 (dd, J = 2.4, 0.8 Hz, 1H), 7.89 (dd, J = 7.7, 1.5 Hz, 1H), 7.68 – 7.33 (m, 4H), 7.10 (dd, J = 8.6, 5.9 Hz, 1H), 5.22 (s, 2H), 4.29 (q, J = 7.1 Hz, 2H), 1.31 (t, J = 7.1 Hz, 3H). **^{13}C NMR** (126 MHz, $CDCl_3$): δ (ppm) 164.4 (dd, J = 11.2, 7.7 Hz), 159.7 (dd, J = 311.7, 298.1 Hz), 141.3, 137.9, 133.8, 132.9, 130.6 (t, J = 2.5 Hz), 128.7, 128.4, 128.2 (t, J = 2.4 Hz), 127.8, 127.5, 126.5, 126.2, 126.1, 125.5, 92.2 (dd, J = 20.5, 10.9 Hz), 61.8, 14.2. **^{19}F NMR** (471 MHz, $CDCl_3$): δ -68.57 (d, J = 10.5 Hz, 1F), -70.76 (d, J = 10.8 Hz, 1F). **HRMS** (ESI) m/z: Calcd for $C_{19}H_{14}F2O_4Na$ $[M+Na]^+$: 367.0757; Found 367.0755.

General procedure (II) for the Hiyama cross-coupling of benzyl/alkyl-substituted *gem*-difluoroalkenes:

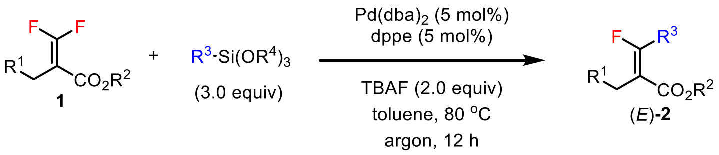

To an oven-dried glass tube equipped with a stir bar was added $Pd(dba)_2$ (5.8 mg, 0.01 mmol) and dppe (4.0 mg, 0.01 mmol). The tube was sealed with a septum, evacuated and refilled with argon three times. A solution of **1** (0.2 mmol) in 1.0 mL toluene was added under argon through syringe, followed by the addition of organosilicon reagents (0.6 mmol) and TBAF (104.6 mg, 0.4 mmol) under argon through syringe. The resulting mixture was heated at 80 °C with stirring in an oil bath for 12 h. After cooling to room temperature, the crude sample was analyzed by ^{19}F NMR using benzotrifluoride (12 μL, 0.1 mmol) as internal standard and the *E/Z* ratio (>99:1) was determined. The crude mixture was extracted with CH_2Cl_2 (3 × 10 mL), the combined organic layers were washed with H_2O (2 × 10 mL) then brine (2 × 10 mL), dried over $MgSO_4$ and concentrated *in vacuo*. The residue was purified by flash column chromatography on silica gel to afford products (*E*)-**2** (*E/Z* >99:1).

General procedure (III) for the Hiyama cross-coupling of (het)aryl-substituted *gem*-difluoroalkenes:

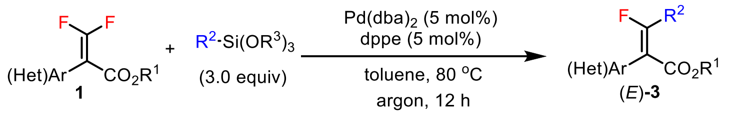

To an oven-dried glass tube equipped with a stir bar was added Pd(dba)$_2$ (5.8 mg, 0.01 mmol) and dppe (4.0 mg, 0.01 mmol). The tube was sealed with a septum, evacuated and refilled with argon three times. A solution of **1** (0.2 mmol) in 1.0 mL toluene was added under argon through syringe, followed by the addition of organosilicon reagents (0.6 mmol) under argon through syringe. The resulting mixture was stirred at 80 °C for 12 h. The crude sample was analyzed by ^{19}F NMR using benzotrifluoride (12 μL, 0.1 mmol) as internal standard and the *E/Z* ratio (>99:1) was determined. The crude mixture was extracted with CH_2Cl_2 (3 × 10 mL), the combined organic layers were washed with H_2O (2 × 10 mL) then brine (2 × 10 mL), dried over $MgSO_4$ and concentrated *in vacuo*. The residue was purified by flash column chromatography on silica gel to afford products (*E*)-**3** (*E/Z* >99:1).

1.0 mmol scale synthesis of (*E*)-2a:

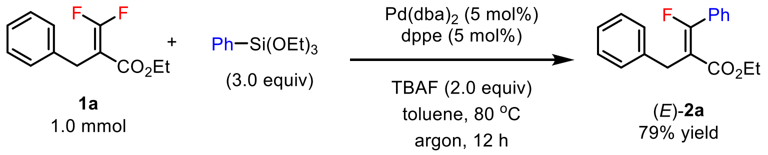

To an oven-dried 25 mL flask equipped with a stir bar was added Pd(dba)$_2$ (57.5 mg, 0.1 mmol) and dppe (39.8 mg, 0.1 mmol). The flask was sealed with a septum, evacuated and refilled with argon three times. A solution of **1a** (226.2 mg, 1.0 mmol) in 10.0 mL toluene was added under argon through syringe, followed by the addition of the triethoxyphenylsilane (721.1 mg, 3.0 mmol) and TBAF (1.046g, 2.0 mmol) under argon through syringe. The resulting mixture was stirred at 80 °C for 12 h. The crude sample was analyzed by ^{19}F NMR using benzotrifluoride (12 μL, 0.1 mmol) as internal standard and the *E/Z* ratio (>99:1) was determined. The reaction mixture was extracted with CH_2Cl_2 (3 × 30 mL). The combined organic layers were washed with H_2O (2 × 30 mL), then brine (2 × 30 mL), dried over $MgSO_4$ and concentrated *in vacuo*. The residue was purified by flash column chromatography on silica gel (DCM : hexane = 1 : 5) to afford (*E*)-**2a** as a colorless oil (224.4 mg, 79%, *E/Z* >99:1).

V. Optimization studies.

Table S1. Optimization studies using 1a.[a]

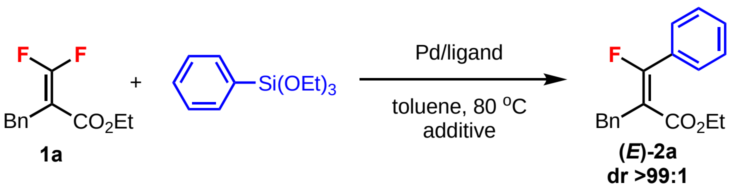

entry	[Pd] (x mol %)	$PhSi(OEt)_3$ (x eq)	Ligand (x mol %)	Additive (x equiv)	Solvent (M)	Temp (°C)/ Time (h)	**2a** (%)[b]
1	$Pd(PPh_3)_4$ (10)	3.0	none	none	toluene (0.2)	80/12	49
2	$Pd(dba)_2$ (10)	3.0	dppe (10)	none	toluene (0.2)	80/12	42
3	$Pd_2(dba)_3$ (5)	3.0	dppe (10)	none	toluene (0.2)	80/12	0
4	$Pd(OAc)_2$ (10)	3.0	none	none	toluene (0.2)	80/12	0
5	$Pd(PPh_3)_4$ (10)	3.0	none	TBAF (2.0)	toluene (0.2)	80/12	0
6	$Pd(dba)_2$ (10)	3.0	dppe (10)	TBAF (2.0)	toluene (0.2)	80/12	99
7	$Pd(dba)_2$ (5)	3.0	dppe (5)	TBAF (2.0)	toluene (0.2)	80/12	97
**8**	**$Pd(dba)_2$ (5)**	**3.0**	**dppe (5)**	**TBAF (2.0)**	**toluene (0.2)**	**80/12**	**98 (iso.96)**[c]
9	none	0	none	TBAF (2.0)	toluene (0.2)	80/12	0
10	none	3.0	none	TBAF (2.0)	toluene (0.2)	80/12	0
11	$Pd(dba)_2$ (5)	3.0	none	TBAF (2.0)	toluene (0.2)	80/12	0
12	none	3.0	dppe (5)	TBAF (2.0)	toluene (0.2)	80/12	0
13	$Pd(dba)_2$ (5)	3.0	dppm (5)	TBAF (2.0)	toluene (0.2)	80/12	56
14	$Pd(dba)_2$ (5)	3.0	dppp (5)	TBAF (2.0)	toluene (0.2)	80/12	0
15	$Pd(dba)_2$ (5)	3.0	dppb (5)	TBAF (2.0)	toluene (0.2)	80/12	0
16	$Pd(dba)_2$ (5)	1.5	dppe (5)	TBAF (2.0)	toluene (0.2)	80/12	74
17	$Pd(dba)_2$ (5)	5.0	dppe (5)	TBAF (2.0)	toluene (0.2)	80/12	99
18	$Pd_2(dba)_3$ (5)	3.0	dppe (10)	TBAF (2.0)	toluene (0.2)	80/12	34
19	$Pd(OAc)_2$ (10)	3.0	None	TBAF (2.0)	toluene (0.2)	80/12	20
20	$Pd(dba)_2$ (5)	3.0	dppe (5)	KF (2.0)	toluene (0.2)	80/12	54
21	$Pd(dba)_2$ (5)	3.0	dppe (5)	AgF (2.0)	toluene (0.2)	80/12	0
22	$Pd(dba)_2$ (5)	3.0	dppe (5)	NaF (2.0)	toluene (0.2)	80/12	48
23	$Pd(dba)_2$ (5)	3.0	dppe (5)	CsF (2.0)	toluene (0.2)	80/12	35
24	$Pd(dba)_2$ (5)	3.0	dppe (5)	CuF_2 (2.0)	toluene (0.2)	80/12	20
25	$Pd(dba)_2$ (5)	3.0	dppe (5)	FeF_3 (2.0)	toluene (0.2)	80/12	28
26	$Pd(dba)_2$ (5)	3.0	dppe (5)	TBAF (2.0)	DMF (0.2)	80/12	12
27	$Pd(dba)_2$ (5)	3.0	dppe (5)	TBAF (2.0)	1,4-dioxane (0.2)	80/12	50

[a]Unless specified otherwise, reactions were carried out using **1a** (0.1 mmol) and triethoxyphenylsilane (0.3 mmol) in toluene (0.2 M) for 12 h under argon. [b]Yield was determined by ^{19}F NMR analysis using benzotrifluoride as the internal standard. The diastereomeric ratio (dr >99:1) was determined by ^{19}F NMR analysis. [c]Isolated yield using 0.2 mmol **1a**.

Derivatizations

Modification of drug molecule isoxepac.

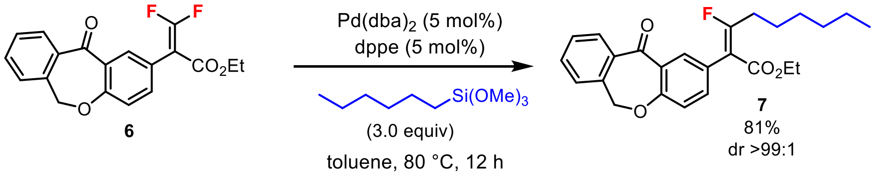

To an oven-dried glass tube equipped with a stir bar was added $Pd(dba)_2$ (5.8 mg, 0.01 mmol) and dppe (4.0 mg, 0.01 mmol). The tube was sealed with a septum, evacuated and refilled with argon three times. A solution of **6** (0.2 mmol) in 1.0 mL toluene was added under argon through syringe, followed by the addition of hexyltrimethoxysilane **SR5** (123.8mg, 0.6 mmol) under argon through syringe. The resulting mixture was stirred at 80 °C for 12 h. The crude sample was analyzed by ^{19}F NMR using benzotrifluoride (12 µL, 0.1 mmol) as internal standard and the *E/Z* ratio (>99:1) was determined. The crude mixture was extracted with CH_2Cl_2 (3 × 10 mL), the combined organic layers were washed with H_2O (2 × 10 mL) then brine (2 × 10 mL), dried over $MgSO_4$ and concentrated *in vacuo*. The residue was purified by flash column chromatography on silica gel to afford products **7** (*E/Z* >99:1).

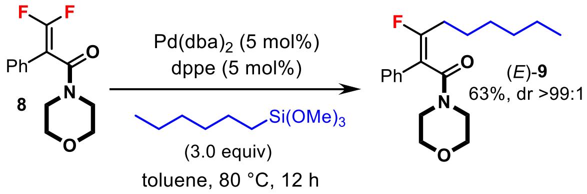

To an oven-dried glass tube equipped with a stir bar was added $Pd(dba)_2$ (5.8 mg, 0.01 mmol) and dppe (4.0 mg, 0.01 mmol). The tube was sealed with a septum, evacuated and refilled with argon three times. A solution of **8** (0.2 mmol) in 1.0 mL toluene was added under argon through syringe, followed by the addition of hexyltrimethoxysilane **SR5** (123.8mg, 0.6 mmol) under argon through syringe. The resulting mixture was stirred at 80 °C for 12 h. The crude sample was analyzed by ^{19}F NMR using benzotrifluoride (12 µL, 0.1 mmol) as internal standard and the *E/Z* ratio (>99:1) was determined. The crude mixture was extracted with CH_2Cl_2 (3 × 10 mL), the combined organic layers were washed with H_2O (2 × 10 mL) then brine (2 × 10 mL), dried over $MgSO_4$ and concentrated *in vacuo*. The residue was purified by flash column chromatography on silica gel to afford products *(E)*-**9** (*E/Z* >99:1).

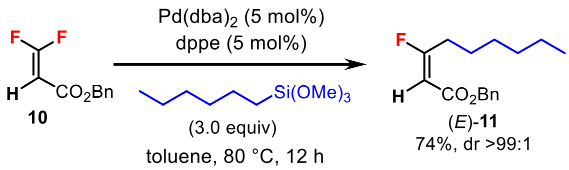

To an oven-dried glass tube equipped with a stir bar was added $Pd(dba)_2$ (5.8 mg, 0.01 mmol) and dppe (4.0 mg, 0.01 mmol). The tube was sealed with a septum, evacuated and refilled with argon three times. A solution of **10** (0.2 mmol) in 1.0 mL toluene was added under argon through syringe, followed by the addition of hexyltrimethoxysilane **SR5** (123.8mg, 0.6 mmol) under argon through syringe. The resulting mixture was stirred at 80 °C for 12 h. The crude sample was analyzed by ^{19}F NMR using benzotrifluoride (12 µL, 0.1 mmol) as internal standard and the *E/Z* ratio (>99:1) was determined. The crude mixture was extracted with CH_2Cl_2 (3 × 10 mL), the combined organic layers were washed with H_2O (2 × 10 mL) then brine (2 × 10 mL), dried over $MgSO_4$ and concentrated *in vacuo*. The residue was purified by flash column chromatography on silica gel to afford products *(E)*-**11** (*E/Z* >99:1).

VI. Characterization Data.

ethyl (*E*)-2-benzyl-3-fluoro-3-phenylacrylate (2a)

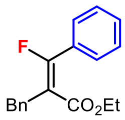

Following the general procedure (II), reaction was run using *gem*-difluoroalkene **1a** (45.2 mg, 0.2 mmol), triethoxyphenylsilane **SR1** (73.2 mg, 0.6 mmol), Pd(dba)$_2$ (5.8 mg, 0.01 mmol), dppe (4.0 mg, 0.01 mmol) and TBAF (104.6 mg, 0.4 mmol) in 1.0 mL toluene for 12 h. The product was purified by flash column chromatography on silica gel (DCM : hexane = 1 : 10) and obtained as a colorless oil (54.6 mg, 96% yield), R_f= 0.28 (DCM : hexane = 1 : 3). **^{1}H NMR**(500 MHz, $CDCl_3$): δ(ppm) 7.37 (d, *J*= 7.4 Hz, 2H), 7.35 -7.31 (m, 3H), 7.21 -7.15 (m, 5H), 3.91 (q, *J*= 7.1 Hz, 2H), 3.77 (d, *J*= 3.2 Hz, 2H), 0.87 (t, *J*= 7.2 Hz, 3H).**^{13}C NMR**(126 MHz, $CDCl_3$): δ(ppm) 167.4 (d, *J*= 16.4 Hz), 164.1 (d, *J*= 262.9 Hz), 139.2 (d, *J*= 2.2 Hz), 132.0 (d, *J*= 27.3 Hz), 130.1 (d, *J*= 2.2 Hz), 128.6, 128.5 (d, *J*= 4.2 Hz), 128.5, 128.0, 126.3, 114.6 (d, *J*= 23.1 Hz), 60.6, 32.5 (d, *J*= 5.0 Hz), 13.5.**^{19}F NMR**(471 MHz, $CDCl_3$): δ(ppm) -85.70 (s, 1F). The spectra are in full accordance with the literature report.[4]

ethyl (*E*)-2-benzyl-3-fluoropenta-2,4-dienoate (2b)

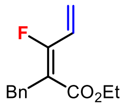

Following the general procedure (II), reaction was run using *gem*-difluoroalkene **1a** (45.2 mg, 0.2 mmol), triethoxyvinylsilane **SR2** (114.2 mg, 0.6 mmol), Pd(dba)$_2$ (5.8 mg, 0.01 mmol), dppe (4.0 mg, 0.01 mmol) and TBAF (104.6 mg, 0.4 mmol) in 1.0 mL toluene for 12 h. The product was purified by flash column chromatography on silica gel (DCM : hexane = 1: 10) and obtained as a colorless oil (37.4 mg, 80% yield), R_f = 0.36 (DCM : hexane = 1: 5). **^{1}H NMR** (500 MHz, $CDCl_3$): δ (ppm) 7.39 - 7.28 (m, 1H), 7.28 - 7.17 (m, 5H), 5.86 (d, *J* = 17.1, Hz, 1H), 5.50 (d, *J* = 11.2, Hz, 1H), 4.16 (q, *J* = 7.1 Hz, 2H), 3.78 (d, *J* = 3.5 Hz, 2H), 1.22 (t, *J* = 7.1 Hz, 3H). **^{13}C NMR** (126 MHz, $CDCl_3$): δ (ppm) 166.4 (d, *J* = 18.0 Hz), 163.8 (d, *J* = 260.7 Hz), 139.5 (d, *J* = 1.8 Hz), 128.5, 128.1, 127.0 (d, *J* = 22.7 Hz), 126.0, 121.1 (d, *J* = 8.8 Hz), 114.3 (d, *J* = 22.2 Hz), 61.0, 31.8 (d, *J* = 7.0 Hz), 14.1. **^{19}F NMR** (471 MHz, $CDCl_3$): δ (ppm) -107.56 (d, *J* = 29.1 Hz). The spectra are in full accordance with the literature report.[5]

benzyl (*E*)-2-benzyl-3-fluorohexa-2,5-dienoate (2c)

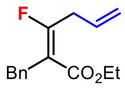

Following the general procedure (II), reaction was run using *gem*-difluoroalkene **1a** (45.2 mg, 0.2 mmol), triethoxyallylsilane **SR3** (122.6 mg, 0.6 mmol), Pd(dba)$_2$ (5.8 mg, 0.01 mmol), dppe (4.0 mg, 0.01 mmol) and TBAF (104.6 mg, 0.4 mmol) in 1.0 mL toluene for 12 h. The product was purified by flash column chromatography on silica gel (DCM : hexane = 1: 5) and obtained as a colorless oil (39.7 mg, 80% yield), R_f = 0.38 (DCM : hexane = 1: 1). **^{1}H NMR** (500 MHz, $CDCl_3$): δ (ppm) 7.25-7.13(m, 5H), 5.89 (ddt,*J*= 16.6, 10.0, 6.5 Hz, 1H), 5.20 (d, *J*= 17.1 Hz, 1H), 5.14 (d, *J*= 10.1,Hz, 1H), 4.12 (q, *J*= 7.1 Hz, 2H), 3.67 (d, *J*=3.6 Hz, 2H), 3.55 (dd, *J*= 26.8, 6.5 Hz, 2H), 1.21 (t, *J*= 7.1 Hz, 3H). **^{13}C NMR** (126 MHz, $CDCl_3$): δ (ppm) 168.9 (d,*J*= 270.2 Hz), 167.0 (d, *J*= 19.0 Hz), 139.9 (d, *J*=2.1 Hz), 131.6, 128.5, 128.4, 126.1, 118.2, 113.5 (d, *J*= 20.7 Hz), 60.9, 35.5 (d, *J*=25.3 Hz), 31.1 (d, *J*= 7.2 Hz), 14.1. **^{19}F NMR** (471 MHz, $CDCl_3$): δ (ppm) -82.38 (tt, *J* = 25.8, 3.7 Hz). The spectra are in full accordance with the literature report.[5]

ethyl (*E*)-2-benzyl-3-fluorohept-2-enoate (2d)

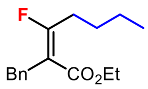

Following the general procedure (II), reaction was run using *gem*-difluoroalkene **1a** (45.2 mg, 0.2 mmol), n-butyltriethoxysilane **SR4** (132.2 mg, 0.6 mmol), Pd(dba)$_2$ (5.8 mg, 0.01 mmol), dppe (4.0 mg, 0.01 mmol) and TBAF (104.6

mg, 0.4 mmol) in 1.0 mL toluene for 12 h. The product was purified by flash column chromatography on silica gel (DCM : hexane = 1 : 10) and obtained as a colorless oil (37.0 mg, 70% yield), R_f = 0.36 (DCM : hexane = 1 : 5). **^{1}H NMR** (500 MHz, $CDCl_3$): δ (ppm) 7.39 - 7.28 (m, 2H), 7.25 - 7.21 (m, 1H), 7.18 (d, J = 7.3 Hz, 2H), 4.12 (q, J = 7.1 Hz, 2H), 3.78 (d, J = 3.6 Hz, 2H), 2.71 (dt, J = 25.9, 7.6 Hz, 2H), 1.60 - 1.58 (m, 2H), 1.42 - 1.30 (m, 2H), 1.18 (t, J = 7.0 Hz, 3H), 0.88 (t, J = 7.2 Hz, 3H). **^{13}C NMR** (126 MHz, $CDCl_3$): δ (ppm) 170.0 (d, J = 271.9 Hz), 167.3 (d, J = 17.6 Hz), 132.9 (d, J = 2.1 Hz), 129.7 (d, J = 2.7 Hz), 128.0, 127.7, 115.6 (d, J = 20.1 Hz), 61.1, 31.8 (d, J = 7.1 Hz), 30.8 (d, J = 24.5 Hz), 28.7, 22.4, 14.2, 14.1. **^{19}F NMR** (471 MHz, $CDCl_3$): δ (ppm) -82.31 (t, J = 25.5 Hz, 1F). The spectra are in full accordance with the literature report.[6]

ethyl (*E*)-2-benzyl-3-fluoronon-2-enoate (2e)

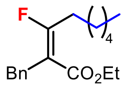

Following the general procedure (II), reaction was run using *gem*-difluoroalkene **1a** (45.2 mg, 0.2 mmol), hexyltrimethoxysilane **SR5** (123.8 mg, 0.6 mmol), $Pd(dba)_2$ (5.8 mg, 0.01 mmol), dppe (4.0 mg, 0.01 mmol) and TBAF (104.6 mg, 0.4 mmol) in 1.0 mL toluene for 12 h. The product was purified by flash column chromatography on silica gel (DCM : hexane = 1 : 10) and obtained as a colorless oil (49.1 mg, 84% yield), R_f = 0.31 (DCM : hexane = 1 : 3). **^{1}H NMR** (500 MHz, $CDCl_3$): δ (ppm) 7.56 - 7.09 (m, 5H), 4.16 (q, J = 7.1 Hz, 2H), 3.70 (d, J = 3.5 Hz, 2H), 2.81 (dt, J = 26.9, 7.6 Hz, 2H), 1.64 (ddd, J = 15.1, 8.3, 5.8 Hz, 2H), 1.45 - 1.27 (m, 6H), 1.24 (t, J = 7.1 Hz, 3H), 0.91 (t, J = 6.8 Hz, 3H). **^{13}C NMR** (126 MHz, $CDCl_3$): δ (ppm) 171.9 (d, J = 270.1 Hz), 167.3 (d, J = 19.6 Hz), 140.0 (d, J = 1.8 Hz), 128.5, 128.2, 126.0, 112.7 (d, J = 21.4 Hz), 60.6, 31.5, 31.1 (d, J = 7.3 Hz), 30.9 (d, J = 24.7 Hz), 28.9, 26.5, 22.5, 14.1, 14.1. **^{19}F NMR** (471 MHz, $CDCl_3$): δ (ppm) -79.8 (t, J = 29.6 Hz, 1F). **HRMS** (ESI) m/z: Calcd for $C_{18}H_{25}FO_2Na$ $[M+Na]^+$: 315.1733; Found 315.1736.

ethyl (*E*)-2-benzyl-3-fluoro-6-methylhept-2-enoate (2f)

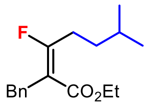

Following the general procedure (II), reaction was run using *gem*-difluoroalkene **1a** (45.2 mg, 0.2 mmol), trimethoxy(3-methylbutyl)silane **SR6** (115.4 mg, 0.6 mmol), $Pd(dba)_2$ (5.8 mg, 0.01 mmol), dppe (4.0 mg, 0.01 mmol) and TBAF (104.6 mg, 0.4 mmol) in 1.0 mL toluene for 12 h. The product was purified by flash column chromatography on silica gel (DCM : hexane = 1 : 5) and obtained as a colorless oil (41.0 mg, 74% yield), R_f = 0.36 (DCM : hexane = 1 : 3). **^{1}H NMR** (500 MHz, $CDCl_3$): δ (ppm) 7.36 - 7.13 (m, 5H), 4.13 (q, J = 7.2 Hz, 2H), 3.75 (d, J = 3.6 Hz, 2H), 2.75 (dt, J = 25.9, 7.6 Hz, 2H), 1.60 - 1.53 (m, 2H), 1.35 - 1.29 (m, J = 7.2 Hz, 1H), 1.17 (m, 6H), 0.87 (t, J = 7.3 Hz, 3H). **^{13}C NMR** (126 MHz, $CDCl_3$): δ (ppm) 170.2 (d, J = 271.9 Hz), 167.1 (d, J = 17.5 Hz), 132.7 (d, J = 2.1 Hz), 129.7 (d, J = 2.7 Hz), 128.1, 127.7, 115.6 (d, J = 20.5 Hz), 61.1, 31.5 (d, J = 7.2 Hz), 30.9 (d, J = 24.6 Hz), 28.7, 22.5, 14.2, 14.2, 14.1. **^{19}F NMR** (471 MHz, $CDCl_3$): δ (ppm) -82.19 (t, J = 25.2 Hz, 1F). The spectra are in full accordance with the literature report.[6]

ethyl (*E*)-2-benzyl-3-fluoro-4,4-dimethylpent-2-enoate (2g)

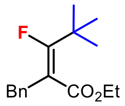

Following the general procedure (II), reaction was run using *gem*-difluoroalkene **1f** (60.46 mg, 0.2 mmol), t-Butyltriethoxysilane **SR7** (132.5 mg, 0.6 mmol), $Pd(dba)_2$ (5.8 mg, 0.01 mmol), dppe (4.0 mg, 0.01 mmol) and TBAF (104.6 mg, 0.4 mmol) in 1.0 mL toluene for 12 h. The product was purified by flash column chromatography on silica gel (DCM : hexane = 1 : 10) and obtained as a colorless oil (38.9 mg, 72% yield), R_f = 0.29 (DCM : hexane = 1 : 5). **^{1}H NMR** (500 MHz, $CDCl_3$): δ (ppm) 7.31 - 7.26 (m, 4H), 7.20 - 7.14 (m, 1H), 4.13 (q, J = 7.1 Hz, 2H), 3.78 (d, J = 3.5 Hz, 2H), 1.20 - 1.18 (m, 12H). **^{13}C NMR** (126 MHz, $CDCl_3$): δ (ppm) 168.3 (d, J = 16.1 Hz), 165.4 (d, J = 266.1 Hz), 133.0 (d, J = 2.6 Hz), 128.4 (d, J = 3.6 Hz), 128.3, 127.7, 114.5 (d, J = 23.5 Hz), 61.2, 36.5 (d, J = 24.8 Hz), 31.4 (d, J = 7.1 Hz), 27.3 (d, J = 4.1 Hz), 14.3. **^{19}F NMR** (471 MHz, $CDCl_3$): δ (ppm) -105.03 (s, 1F). The spectra are in full accordance with the literature report.[6]

ethyl (*E*)-3-fluoro-2-(2-methylheptyl)non-2-enoate (2h)

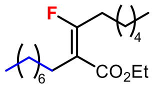

Following the general procedure (II), reaction was run using *gem*-difluoroalkene **1h** (35.6 mg, 0.2 mmol), hexyltrimethoxysilane **SR5** (123.8 mg, 0.6 mmol), Pd(dba)$_2$ (5.8 mg, 0.01 mmol), dppe (4.0 mg, 0.01 mmol) and TBAF (104.6 mg, 0.4 mmol) in 1.0 mL toluene for 12 h. The product was purified by flash column chromatography on silica gel (DCM : hexane = 1 : 8) and obtained as a colorless oil (50.3 mg, 80% yield), R_f = 0.30 (DCM : hexane = 1 : 5). **^{1}H NMR** (500 MHz, CDCl$_3$): δ (ppm) 4.22 (q, J = 7.1 Hz, 2H), 2.71 (dt, J = 26.8, 7.6 Hz, 2H), 2.41 - 2.23 (m, 2H), 1.72 - 1.52 (m, 2H), 1.44 - 1.13 (m, 21H), 0.90 (td, J = 7.0, 2.5 Hz, 6H). **^{13}C NMR** (126 MHz, CDCl$_3$): δ (ppm) 170.9 (d, J = 267.8 Hz), 168.1 (d, J = 20.1 Hz), 113.7 (d, J = 22.1 Hz), 60.5, 31.8 (d, J = 43.0 Hz), 31.0 (d, J = 25.3 Hz), 29.5, 29.4, 29.2 (d, J = 1.7 Hz), 29.0, 26.7, 25.5 (d, J = 6.7 Hz), 22.7 (d, J = 18.2 Hz), 14.3, 14.2, 14.2. **^{19}F NMR** (471 MHz, CDCl$_3$): δ (ppm) -82.57 (t, J = 26.6 Hz, 1F). **HRMS** (ESI) m/z: Calcd for $C_{19}H_{35}FO_2Na$ [M+Na]$^+$: 337.2521; Found 337.2518.

ethyl (*E*)-3-fluoro-2-(4-methoxybenzyl)non-2-enoate (2i)

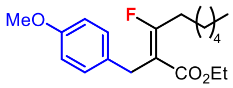

Following the general procedure (II), reaction was run using *gem*-difluoroalkene **1i** (51.2 mg,0.2 mmol), hexyltrimethoxysilane **SR5** (123.8 mg, 0.6 mmol), Pd(dba)$_2$ (5.8 mg, 0.01 mmol), dppe (4.0 mg, 0.01 mmol) and TBAF (104.6 mg, 0.4 mmol) in 1.0 mL toluene for 12 h. The product was purified by flash column chromatography on silica gel (DCM : hexane = 1 : 5) and obtained as a colorless oil (53.3 mg, 83% yield), R_f = 0.35 (DCM : hexane = 1 : 1). **^{1}H NMR** (500 MHz, CDCl$_3$): δ (ppm) 7.15 (s, 2H), 6.83 (d, J = 8.6 Hz, 2H), 4.16 (q, J = 7.1 Hz, 2H), 3.80 (s, 3H), 3.63 (d, J = 3.5 Hz, 2H), 2.79 (dt, J = 26.9, 7.6 Hz, 2H), 1.64 (m, J = 7.5 Hz, 2H), 1.44 - 1.29 (m, 6H), 1.26 (t, J = 7.1 Hz, 3H), 0.91 (t, J = 6.8 Hz, 3H). **^{13}C NMR** (126 MHz, CDCl$_3$): δ (ppm) 171.7 (d, J = 269.7 Hz), 167.5 (d, J = 19.5 Hz), 158.0, 132.2 (d, J = 1.9 Hz), 129.6, 113.8, 113.3 (d, J = 21.5 Hz), 60.7, 55.3, 31.6, 31.0 (d, J = 24.9 Hz), 30.4 (d, J = 7.4 Hz), 29.0, 26.7, 22.7, 14.2, 14.2. **^{19}F NMR** (471 MHz, CDCl$_3$): δ (ppm) -80.50 (t, J = 26.9 Hz, 1F). **HRMS** (ESI) m/z: Calcd for $C_{19}H_{27}FO_3Na$ [M+Na]$^+$: 345.1839; Found 345.1841.

ethyl (*E*)-3-fluoro-2-(4-(trifluoromethyl)benzyl)non-2-enoate (2j)

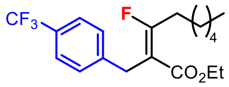

Following the general procedure (II), reaction was run using *gem*-difluoroalkene **1j** (58.8 mg, 0.2 mmol), hexyltrimethoxysilane **SR5** (123.8 mg, 0.6 mmol), Pd(dba)$_2$ (5.8 mg, 0.01 mmol), dppe (4.0 mg, 0.01 mmol) and TBAF (104.6 mg, 0.4 mmol) in 1.0 mL toluene for 12 h. The product was purified by flash column chromatography on silica gel (DCM : hexane = 1: 4) and obtained as a yellow oil (40.3 mg, 56% yield), R_f = 0.38 (DCM : hexane = 1: 1). **^{1}H NMR** (500 MHz, CDCl$_3$): δ (ppm) 7.53 (d, J = 8.1 Hz, 2H), 7.35 (d, J = 8.0 Hz, 2H), 4.17 (q, J = 7.1 Hz, 2H), 3.74 (d, J = 3.4 Hz, 2H), 2.82 (dt, J = 27.1, 7.6 Hz, 2H), 1.76 - 1.54 (m, 2H), 1.47 - 1.28 (m, 6H), 1.24 (t, J = 7.1 Hz, 3H), 0.92 - 0.89 (m, 3H). **^{13}C NMR** (126 MHz, CDCl$_3$): δ (ppm) 172.6 (d, J = 271.1 Hz), 166.9 (d, J = 19.3 Hz), 144.2, 128.7, 128.4 (q, J = 32.2 Hz), 125.2 (q, J = 3.8 Hz), 127.6 - 120.9 (m), 112.0 (d, J = 21.2 Hz), 60.8, 31.5, 31.1 (d, J = 7.5 Hz), 30.9 (d, J = 24.6 Hz), 28.9, 26.5, 22.5, 14.1, 14.0. **^{19}F NMR** (471 MHz, CDCl$_3$): δ (ppm) -62.34 (s, 3H), -78.26 (tt, J = 27.8, 3.8 Hz, 1H). **HRMS** m/z (ESI): calcd. for $C_{19}H_{24}F_4O_2Na$ [M+Na]$^+$: 383.1600 found: 383.1609.

ethyl (*E*)-3-fluoro-2-(naphthalen-2-ylmethyl)non-2-enoate (2k)

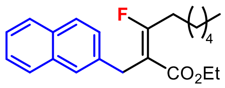

Following the general procedure (II), reaction was run using *gem*-difluoroalkene **1k** (55.2 mg, 0.2 mmol), hexyltrimethoxysilane **SR5** (123.8 mg, 0.6 mmol), Pd(dba)$_2$ (5.8 mg, 0.01 mmol), dppe (4.0 mg, 0.01 mmol) and TBAF (104.6 mg, 0.4 mmol) in 1.0 mL toluene for 12 h. The product was purified by flash column chromatography on silica gel (DCM :

hexane = 1: 5) and obtained as a colorless oil (52.2 mg, 76% yield), R_f = 0.30 (DCM : hexane = 1: 2). **^{1}H NMR** (500 MHz, $CDCl_3$): δ (ppm) 7.96 - 7.74 (m, 3H), 7.67 (s, 1H), 7.53 - 7.36 (m, 3H), 4.16 (q, J = 7.1 Hz, 2H), 3.86 (d, J = 3.5 Hz, 2H), 2.84 (dt, J = 26.9, 7.6 Hz, 2H), 1.67 (tt, J = 7.9, 6.5 Hz, 2H), 1.49 - 1.30 (m, 6H), 1.24 (t, J = 7.1 Hz, 3H), 0.91 (t, J = 6.8 Hz,3H). **^{13}C NMR** (101 MHz, $CDCl_3$): δ (ppm) 172.0 (d, J = 270.2 Hz), 167.3 (d, J = 19.5 Hz), 137.5 (d, J = 1.9 Hz), 133.5, 132.1, 127.8, 127.6 (d, J = 3.5 Hz), 127.3, 126.7, 125.8, 125.2, 112.7 (d, J = 21.4 Hz), 60.6, 31.5, 31.3 (d, J = 7.5 Hz), 30.9 (d, J = 24.8 Hz), 28.9, 26.6, 22.5, 14.1, 14.1. **^{19}F NMR** (471 MHz, $CDCl_3$): δ (ppm) -79.51 (t, J = 26.9 Hz, 1F). **HRMS** m/z (ESI): calcd. for $C_{22}H_{27}FO_2Na$ $[M+Na]^+$: 365.1896; found: 365.1892.

methyl (*E*)-2-(2-(ethoxycarbonyl)-3-fluoronon-2-en-1-yl) benzoate (2l)

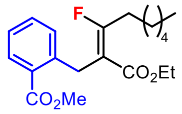

Following the general procedure (II), reaction was run using *gem*-difluoroalkene **1l** (56.8 mg, 0.2 mmol), hexyltrimethoxysilane **SR5** (123.8 mg, 0.6 mmol), $Pd(dba)_2$ (5.8 mg, 0.01 mmol), dppe (4.0 mg, 0.01 mmol) and TBAF (104.6 mg, 0.4 mmol) in 1.0 mL toluene for 12 h. The product was purified by flash column chromatography on silica gel (DCM : hexane = 1: 2) and obtained as a colorless oil (50.6 mg, 86% yield), R_f = 0.29 (DCM : hexane = 1: 1). **^{1}H NMR** (400 MHz, $CDCl_3$): δ (ppm) 7.86 (dd, J = 7.8, 1.5 Hz, 1H), 7.42 (td, J = 7.6, 1.5 Hz, 1H), 7.28 - 7.16 (m, 2H), 4.16 - 4.04 (m, 4H), 3.93 (s, 3H), 2.82 (dt, J = 26.7, 7.6 Hz, 2H), 1.97 - 1.56 (m, 2H), 1.49 - 1.26 (m, 6H), 1.14 (t, J = 7.1 Hz, 3H), 0.83 (t, 7.5 Hz, 3H). **^{13}C NMR** (126 MHz, $CDCl_3$): δ (ppm) 172.2 (d, J = 270.6 Hz), 168.5, 167.4 (d, J = 19.4 Hz), 141.2 (d, J = 1.9 Hz), 131.9, 130.5, 130.1, 129.0, 126.0, 112.2 (d, J = 21.1 Hz), 60.7, 52.1, 31.6, 30.9 (d, J = 24.7 Hz), 29.0, 28.9, 26.7, 22.7, 14.2, 14.1. **^{19}F NMR** (471 MHz, $CDCl_3$): δ (ppm) -80.15 (t, J = 26.7 Hz, 1F). **HRMS** m/z (ESI): calcd. for $C_{20}H_{27}FO_4Na$ $[M+Na]^+$: 373.1788; found: 373.1790.

ethyl (*E*)-3-fluoro-2-phenethylnon-2-enoate (2m)

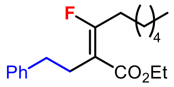

Following the general procedure (II), reaction was run using *gem*-difluoroalkene **1m** (48.0 mg, 0.2 mmol), hexyltrimethoxysilane **SR5** (123.8 mg, 0.6 mmol), $Pd(dba)_2$ (5.8 mg, 0.01 mmol), dppe (4.0 mg, 0.01 mmol) and TBAF (104.6 mg, 0.4 mmol) in 1.0 mL toluene for 12 h. The product was purified by flash column chromatography on silica gel (DCM : hexane = 1: 5) and obtained as a colorless oil (48.4 mg, 79% yield), R_f = 0.35 (DCM : hexane = 1: 5). **^{1}H NMR** (400 MHz, $CDCl_3$): δ (ppm) 7.85 - 6.82 (m, 5H), 4.21 (q, J = 7.1 Hz, 2H), 3.11 - 2.37 (m, 6H), 1.84 - 1.49 (m, 2H), 1.42 - 1.26 (m, 9H), 0.89 (t, 7.1 Hz, 3H). **^{13}C NMR** (126 MHz, $CDCl_3$): δ (ppm) 1 171.8 (d, J = 269.4 Hz), 167.7 (d, J = 20.2 Hz), 141.8, 128.7, 128.4, 126.0, 112.8 (d, J = 21.8 Hz), 60.6, 35.5 (d, J = 1.6 Hz), 31.7, 31.0 (d, J = 24.9 Hz), 29.0, 27.7 (d, J = 6.7 Hz), 26.6, 22.7, 14.4, 14.2. **^{19}F NMR** (471 MHz, $CDCl_3$): δ (ppm) -80.75 (t, J = 26.9 Hz, 1F). **HRMS** m/z (ESI): calcd. for $C_{19}H_{27}FO_2Na$ $[M+Na]^+$:329.1890; found: 329.1892.

tert-butyl (*E*)-2-benzyl-3-fluoronon-2-enoate (2n)

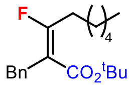

Following the general procedure (II), reaction was run using *gem*-difluoroalkene **1n** (50.7 mg, 0.2 mmol), hexyltrimethoxysilane **SR5** (123.8 mg, 0.6 mmol), $Pd(dba)_2$ (5.8 mg, 0.01 mmol), dppe (4.0 mg, 0.01 mmol) and TBAF (104.6 mg, 0.4 mmol) in 1.0 mL toluene for 12 h. The product was purified by flash column chromatography on silica gel (DCM : hexane = 1: 5) and obtained as a colorless oil (55.9 mg, 86% yield), R_f = 0.37 (DCM : hexane = 1: 3). **^{1}H NMR** (500 MHz, $CDCl_3$): δ (ppm) 7.31 - 7.15 (m, 5H), 3.65 (d, J = 3.4 Hz, 2H), 2.76 (dt, J = 26.7, 7.6 Hz, 2H), 1.71 - 1.53 (m, 2H), 1.40 (s, 9H), 1.42 – 1.17 (m, 6H), 1.07 - 0.67 (m, 3H). **^{13}C NMR** (126 MHz, $CDCl_3$): δ (ppm) 170.5 (d, J = 268.2 Hz), 166.5 (d, J = 19.1 Hz), 140.1 (d, J = 1.8 Hz), 128.4, 128.2, 125.9, 114.0 (d, J = 20.2 Hz), 81.0, 31.6, 31.4 (d, J = 7.7 Hz), 30.7 (d, J = 25.2 Hz), 28.9, 28.0, 26.5, 22.6, 14.1. **^{19}F NMR** (471 MHz, $CDCl_3$): δ (ppm) -83.43 (t, J = 29.6 Hz, 1F). **HRMS** m/z (ESI): calcd. for $C_{20}H_{29}FO_2Na$ $[M+Na]^+$: 343.2044; found: 343.2048.

ethyl (*E*)-3-fluoro-2,3-diphenylacrylate (3a)

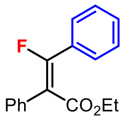

Following the general procedure (III), reaction was run using *gem*-difluoroalkene **1b** (44.8 mg, 0.2 mmol), triethoxyphenylsilane **SR1** (73.2 mg,0.6 mmol), Pd(dba)$_2$ (5.8 mg, 0.01 mmol) and dppe (4.0 mg, 0.01 mmol) in 1.0 mL toluene for 12 h. The product was purified by flash column chromatography on silica gel (DCM : hexane = 1 : 10) and obtained as a colorless oil(49.1 mg, 91% yield), *R*f=0.43 (DCM : hexane = 1 : 1). **^{1}H NMR**(500 MHz, CDCl$_3$): δ(ppm)7.56 (d, *J*= 7.2 Hz, 2H), 7.51 -7.44 (m, 7H), 7.36 (t, *J*= 7.2 Hz, 1H), 4.12 (q, *J*= 7.1 Hz, 2H), 1.09 (t, *J*= 7.1 Hz, 3H).**^{13}C NMR**(101 MHz, CDCl$_3$): δ(ppm)168.1 (d, *J*= 14.9 Hz), 160.9 (d, *J*= 265.0 Hz), 132.3, 132.1 (d, *J*= 27.2 Hz), 130.5 (d, *J*= 1.7 Hz), 129.2 (d, *J*= 3.9 Hz), 128.5, 128.3, 128.2, 128.1 (d, *J*= 4.6 Hz), 116.6 (d, *J*= 20.2 Hz), 61.5, 13.7.**^{19}F NMR**(377 MHz, CDCl$_3$): δ(ppm)-93.69(s, 1F). The spectra are in full accordance with the literature report.[4]

ethyl (*E*)-3-fluoro-2-phenylpenta-2,4-dienoate (3b)

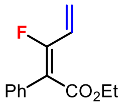

Following the general procedure (III), reaction was run using *gem*-difluoroalkene **1b** (44.8 mg, 0.2 mmol), triethoxyvinylsilane **SR2** (114.2 mg, 0.6 mmol), Pd(dba)$_2$ (5.8 mg, 0.01 mmol) and dppe (4.0 mg, 0.01 mmol) in 1.0 mL toluene for 12 h. The product was purified by flash column chromatography on silica gel (DCM : hexane = 1: 10) and obtained as a colorless oil (23.3 mg, 53% yield), R_f = 0.35 (DCM : hexane = 1: 5). **^{1}H NMR** (500 MHz, CDCl$_3$): δ (ppm) 7.39 - 7.29 (m, 5H), 7.21 (ddd, *J* = 28.2, 17.2, 11.2 Hz, 1H), 5.90 (d, *J* = 17.3 Hz, 1H), 5.56 (d, *J* = 11.1 Hz, 1H), 4.24 (q, *J* = 7.1 Hz, 2H), 1.24 (t, *J* = 7.1 Hz, 3H). **^{13}C NMR** (126 MHz, CDCl$_3$): δ (ppm) 166.9 (d, *J* = 16.0 Hz), 161.5 (d, *J* = 263.0 Hz), 132.4, 129.8 (d, *J* = 3.2 Hz), 128.1, 128.0, 126.7 (d, *J* = 22.6 Hz), 121.7 (d, *J* = 8.2 Hz), 116.2 (d, *J* = 20.3 Hz), 61.4, 14.1. **^{19}F NMR** (471 MHz, CDCl$_3$): δ (ppm) -109.57 (d, *J* = 27.7 Hz). The spectra are in full accordance with the literature report.[5]

ethyl (*E*)-3-fluoro-2-phenylpenta-2,4-dienoate (3c)

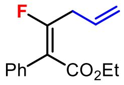

Following the general procedure (III), reaction was run using *gem*-difluoroalkene **1b** (44.8 mg, 0.2 mmol), triethoxyallylsilane **SR3** (122.6 mg, 0.6 mmol), Pd(dba)$_2$ (5.8 mg, 0.01 mmol) and dppe (4.0 mg, 0.01 mmol) in 1.0 mL toluene for 12 h. The product was purified by flash column chromatography on silica gel (DCM : hexane = 1: 10) and obtained as a colorless oil (36.9 mg, 79% yield), R_f = 0.33 (DCM : hexane = 1: 5). **^{1}H NMR** (500 MHz, CDCl$_3$): δ (ppm) 7.39 - 7.28 (m, 5H), 5.92 (ddt, *J* = 16.7, 10.0, 6.5 Hz, 1H), 5.27 (d, *J* = 17.1 Hz, 1H), 5.20 (d, *J* = 10.1 Hz, 1H), 4.20 (q, *J* = 7.1 Hz, 2H), 3.55 (dd, *J* = 25.3, 6.5 Hz, 2H), 1.22 (t, *J* = 7.1 Hz, 3H). **^{13}C NMR** (126 MHz, CDCl$_3$): δ (ppm) 167.2 (d, *J* = 272.5 Hz), 167.0 (d, *J* = 16.9 Hz), 132.4, 131.4, 129.8 (d, *J* = 2.7 Hz), 128.0, 127.8, 118.4, 116.2 (d, *J* = 19.8 Hz), 61.3, 35.6 (d, *J* = 25.0 Hz), 14.2. **^{19}F NMR** (471 MHz, CDCl$_3$): δ (ppm) -84.01 (t, *J* = 25.2 Hz). The spectra are in full accordance with the literature report.[5]

ethyl (*E*)-3-fluoro-2-phenylhept-2-enoate (3d)

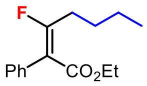

Following the general procedure (III), reaction was run using *gem*-difluoroalkene **1b** (44.8 mg, 0.2 mmol), n-butyltriethoxysilane **SR4** (132.2 mg, 0.6 mmol), Pd(dba)$_2$ (5.8 mg, 0.01 mmol) and dppe (4.0 mg, 0.01 mmol) in 1.0 mL toluene for 12 h. The product was purified by flash column chromatography on silica gel (DCM : hexane = 1 : 15) and obtained as a colorless oil (36.0 mg, 72% yield), R_f = 0.36 (DCM : hexane = 1 : 4). **^{1}H NMR** (500 MHz, CDCl$_3$): δ (ppm) 7.32 - 7.25 (m, 2H), 7.23 - 7.20 (m, 1H), 7.19 (d, *J* = 7.2 Hz, 2H), 4.12 (q, *J* = 7.1 Hz, 2H), 2.74 (dt, *J* = 25.8, 7.6 Hz, 2H), 1.61 - 1.58 (m, 2H), 1.40 - 1.32 (m, 2H), 1.14 (t, *J* = 7.2 Hz, 3H), 0.89 (t, *J* = 7.2 Hz, 3H). **^{13}C NMR** (126 MHz, CDCl$_3$): δ (ppm) 170.2 (d, *J* = 271.8 Hz), 167.4 (d, *J* = 17.5 Hz), 132.9 (d, *J* = 2.2 Hz), 129.8 (d, *J* = 2.7 Hz), 128.2, 127.5, 115.6 (d, *J* = 20.5

Hz), 61.0, 30.9 (d, *J* = 24.5 Hz), 28.7, 22.4, 14.2, 14.1. **^{19}F NMR** (471 MHz, $CDCl_3$): *δ* (ppm) -82.31 (t, *J* = 25.7 Hz, 1F). The spectra are in full accordance with the literature report.[6]

ethyl (*E*)-3-fluoro-2-phenylnon-2-enoate (3e)

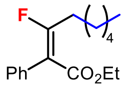

Following the general procedure (III), reaction was run using *gem*-difluoroalkene **1b** (44.8 mg, 0.2 mmol), hexyltrimethoxysilane **SR5** (123.8 mg, 0.6 mmol), $Pd(dba)_2$ (5.8 mg, 0.01 mmol) and dppe (4.0 mg, 0.01 mmol) in 1.0 mL toluene for 12 h. The product was purified by flash column chromatography on silica gel (DCM : hexane = 1 : 10) and obtained as a colorless oil (41.1 mg, 74% yield), R_f = 0.39 (DCM : hexane = 1 : 4). **^{1}H NMR** (500 MHz, $CDCl_3$): *δ* (ppm) 7.44 - 7.18 (m, 5H), 4.23 (q, *J* = 7.1 Hz, 2H), 2.82 (dt, *J* = 25.8, 7.6 Hz, 2H), 1.69 (ddt, *J* = 8.9, 7.6, 3.8 Hz, 2H), 1.51 - 1.39 (m, 2H), 1.35 (ddd, *J* = 7.1, 4.4, 3.1 Hz, 4H), 1.26 (t, *J* = 7.1 Hz, 3H), 1.00 - 0.68 (m, 3H). **^{13}C NMR** (126 MHz, $CDCl_3$): *δ* (ppm) 170.1 (d, *J* = 272.1 Hz), 167.4 (d, *J* = 17.6 Hz), 132.9 (d, *J* = 2.2 Hz), 129.8 (d, *J* = 2.7 Hz), 128.1, 127.6, 115.6 (d, *J* = 20.2 Hz), 61.1, 31.7, 31.2 (d, *J* = 24.6 Hz), 29.0, 26.6, 14.3, 14.2. **^{19}F NMR** (471 MHz, $CDCl_3$): *δ* (ppm) -81.32 (d, *J* = 26.0 Hz, 1F). **HRMS** (ESI) m/z: Calcd for $C_{17}H_{23}FO_2Na$ $[M+Na]^+$: 301.1578; Found 301.1579.

ethyl (*E*)-3-fluoro-6-methyl-2-phenylhept-2-enoate (3f)

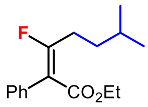

Following the general procedure (III), reaction was run using *gem*-difluoroalkene **1b** (44.8 mg, 0.2 mmol), trimethoxy(3-methylbutyl)silane **SR6** (115.4 mg, 0.6 mmol), $Pd(dba)_2$ (5.8 mg, 0.01 mmol) and dppe (4.0 mg, 0.01 mmol) in 1.0 mL toluene for 12 h. The product was purified by flash column chromatography on silica gel (DCM : hexane = 1 : 15) and obtained as a colorless oil (36.0 mg, 75% yield), R_f = 0.36 (DCM : hexane = 1 : 4). **^{1}H NMR** (500 MHz, $CDCl_3$): *δ* (ppm) 7.31 - 7.28 (m, 2H), 7.24 - 7.18 (m, 3H), 4.14 (q, *J* = 7.1 Hz, 2H), 2.74 (dt, *J* = 26.0, 7.6 Hz, 2H), 1.62 - 1.56 (m, 2H), 1.41 - 1.39 (m, 1H), 1.17 (d, *J* = 7.0 Hz, 6H), 0.89 (t, *J* = 7.3 Hz, 3H). **^{13}C NMR** (126 MHz, $CDCl_3$): *δ* (ppm) 170.2 (d, *J* = 271.9 Hz), 167.5 (d, *J* = 17.6 Hz), 132.8 (d, *J* = 2.2 Hz), 129.8 (d, *J* = 2.7 Hz), 128.0, 127.5, 115.4 (d, *J* = 20.4 Hz), 61.1, 31.0 (d, *J* = 24.6 Hz), 28.6, 22.5, 14.2, 14.2, 14.1. **^{19}F NMR** (471 MHz, $CDCl_3$): *δ* (ppm) -80.79 (t, *J* = 25.0 Hz, 1F). The spectra are in full accordance with the literature report.[6]

ethyl (*E*)-3-cyclohexyl-3-fluoro-2-phenylacrylate (3g)

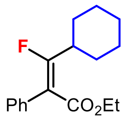

Following the general procedure (III), reaction was run using *gem*-difluoroalkene **1b** (44.8 mg, 0.2 mmol), Triethoxysilyl)cyclohexane **SR8** (147.4 mg, 0.6 mmol), $Pd(dba)_2$ (5.8 mg, 0.01 mmol) and dppe (4.0 mg, 0.01 mmol) in 1.0 mL toluene for 12 h. The product was purified by flash column chromatography on silica gel (DCM : hexane = 1 : 10) and obtained as a colorless oil (40.8 mg, 72% yield), R_f = 0.37 (DCM : hexane = 1 : 3). **^{1}H NMR** (500 MHz, $CDCl_3$): *δ* (ppm) 7.29 (t, *J* = 7.2 Hz, 2H), 7.24 - 7.19 (m, 3H), 4.12 (q, *J* = 7.1 Hz, 2H), 3.20 - 3.14 (m, 1H), 1.80 - 1.73 (m, 4H), 1.63 (d, *J* = 12.7 Hz, 1H), 1.50 - 1.41 (m, 3H), 1.28 (q, *J* = 12.8 Hz, 2H), 1.14 (t, *J* = 7.1 Hz, 3H). **^{13}C NMR** (126 MHz, $CDCl_3$): *δ* (ppm) 172.1 (d, *J* = 275.2 Hz), 167.4 (d, *J* = 17.4 Hz), 132.8 (d, *J* = 2.1 Hz), 129.9 (d, *J* = 3.0 Hz), 128.1, 127.5, 114.2 (d, *J* = 20.6 Hz), 61.1, 39.9 (d, *J* = 22.8 Hz), 29.2, 26.0, 25.9, 14.1. **^{19}F NMR** (471 MHz, $CDCl_3$): *δ* (ppm) -95.88 (d, *J* = 31.7 Hz, 1F). The spectra are in full accordance with the literature report.[6]

ethyl (*E*)-3-cyclopentyl-3-fluoro-2-phenylacrylate (3h)

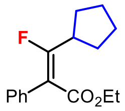

Following the general procedure (III), reaction was run using *gem*-difluoroalkene **1b** (44.8 mg, 0.2 mmol),

cyclopentyltriethoxysilane **SR9** (139.4 mg, 0.6 mmol), $Pd(dba)_2$ (5.8 mg, 0.01 mmol) and dppe (4.0 mg, 0.01 mmol) in 1.0 mL toluene for 12 h. The product was purified by flash column chromatography on silica gel (DCM : hexane = 1 : 10) and obtained as a colorless oil (34.6 mg, 65% yield), R_f = 0.41 (DCM : hexane = 1 : 2). **^{1}H NMR** (500 MHz, $CDCl_3$): δ (ppm) 7.26 (t, J = 7.2 Hz, 2H), 7.20 (dd, J = 14.7, 6.9 Hz, 3H), 4.14 (q, J = 7.1 Hz, 2H), 3.68 - 3.56 (m, 1H), 1.87 (q, J = 8.2 Hz, 2H), 1.71 - 1.63 (m, 4H), 1.58 - 1.55 (m, 2H), 1.18 (t, J = 7.1 Hz, 3H). **^{13}C NMR** (126 MHz, $CDCl_3$): δ (ppm) 171.1 (d, J = 273.1 Hz), 167.8 (d, J = 17.1 Hz), 133.0 (d, J = 1.9 Hz), 129.8 (d, J = 2.8 Hz), 128.1, 127.5, 114.7 (d, J = 21.2 Hz), 61.1, 40.2 (d, J = 24.1 Hz), 30.1 (d, J = 1.8 Hz), 26.4, 14.3. **^{19}F NMR** (471 MHz, $CDCl_3$): δ (ppm) -99.12 (d, J = 35.2 Hz, 1F). The spectra are in full accordance with the literature report.[6]

ethyl (*E*)-3-fluoro-4,4-dimethyl-2-phenylpent-2-enoate (3i)

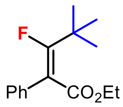

Following the general procedure (III), reaction was run using *gem*-difluoroalkene **1b** (44.8 mg, 0.2 mmol), t-butyl-triethoxysilane **SR7** (132.4 mg, 0.6 mmol), $Pd(dba)_2$ (5.8 mg, 0.01 mmol) and dppe (4.0 mg, 0.01 mmol) in 1.0 mL toluene for 12 h. The product was purified by flash column chromatography on silica gel (DCM : hexane = 1 : 10) and obtained as a colorless oil (31.9 mg, 62% yield), R_f = 0.37 (DCM : hexane = 1 : 3). **^{1}H NMR** (500 MHz, $CDCl_3$): δ (ppm) 7.31 - 7.24 (m, 4H), 7.20 - 7.16 (m, 1H), 4.12 (q, J = 7.2 Hz, 2H), 1.22 - 1.19 (m, 12H). **^{13}C NMR** (126 MHz, $CDCl_3$): δ (ppm) 167.8 (d, J = 16.2 Hz), 165.6 (d, J = 266.3 Hz), 133.0 (d, J = 2.6 Hz), 128.9 (d, J = 3.6 Hz), 128.3, 127.7, 114.3 (d, J = 23.4 Hz), 61.4, 36.7 (d, J = 25.0 Hz), 27.5 (d, J = 4.2 Hz), 14.3. **^{19}F NMR** (471 MHz, $CDCl_3$): δ (ppm) -106.69 (s, 1F). The spectra are in full accordance with the literature report.[6]

ethyl (*E*)-7-(diethylamino)-3-fluoro-2-phenylhept-2-enoate (3j)

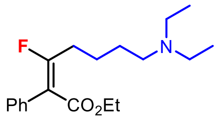

Following the general procedure (III), reaction was run using *gem*-difluoroalkene **1b** (44.8 mg, 0.2 mmol), N,N-Diethyl-3-Triethoxysilylpropan-1-Amine **SR10** (166.4 mg, 0.6 mmol), $Pd(dba)_2$ (5.8 mg, 0.01 mmol) and dppe (4.0 mg, 0.01 mmol) in 1.0 mL toluene for 12 h. The product was purified by flash column chromatography on silica gel (DCM : hexane = 1 : 15) and obtained as a colorless oil (36.0 mg, 56% yield), R_f = 0.37 (DCM : hexane = 1 : 1). **^{1}H NMR** (500 MHz, $CDCl_3$): δ (ppm) 7.32 - 7.28 (m, 2H), 7.25 - 7.23 (m, 1H), 7.20 (d, J = 7.2 Hz, 2H), 4.12 (q, J = 7.2 Hz, 2H), 3.98 (m, 4H), 3.76 (m, 2H), 2.73 (dt, J = 25.8, 7.5 Hz, 2H), 1.60 - 1.53 (m, 2H), 1.39 (m, 2H), 1.14 (d, J = 7.0 Hz, 6H), 0.89 (t, J = 7.2 Hz, 3H). **^{13}C NMR** (126 MHz, $CDCl_3$): δ (ppm) 170.5 (d, J = 271.9 Hz), 167.3 (d, J = 17.5 Hz), 133.1 (d, J = 2.2 Hz), 129.9 (d, J = 2.7 Hz), 128.1, 127.5, 115.6 (d, J = 20.5 Hz), 61.2, 32.1, 31.0 (d, J = 24.8 Hz), 28.5, 22.6, 14.3, 14.3, 14.1. **^{19}F NMR** (471 MHz, $CDCl_3$): δ (ppm) -83.63 (t, J = 25.7 Hz, 1F). **HRMS** (ESI) m/z: Calcd for $C_{19}H_{28}FNO_2Na$ $[M+Na]^+$: 344.1998; Found 344.2001.

ethyl (*E*)-3-fluoro-2-phenyl-5-(phenylamino)pent-2-enoate (3k)

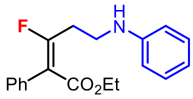

Following the general procedure (III), reaction was run using *gem*-difluoroalkene **1b** (44.8 mg, 0.2 mmol), (2-Anilinoethyl)(trimethoxy)silane **SR11** (166.4 mg, 0.6 mmol), $Pd(dba)_2$ (5.8 mg, 0.01 mmol) and dppe (4.0 mg, 0.01 mmol) in 1.0 mL toluene for 12 h. The product was purified by flash column chromatography on silica gel (DCM : hexane = 1 : 15) and obtained as a colorless oil (13.6 mg, 21% yield), R_f = 0.36 (DCM : hexane = 1 : 1). **^{1}H NMR** (500 MHz, $CDCl_3$): δ (ppm) 7.36 - 7.21 (m, 10H), 4.13 (q, J = 7.1 Hz, 2H), 3.87 - 3.76 (m, 1H), 2.73 (dt, J = 25.9, 7.6 Hz, 2H), 1.39 (m, 2H), 1.18 (t, J = 7.1 Hz, 3H). **^{13}C NMR** (126 MHz, $CDCl_3$): δ (ppm) 172.1 (d, J = 271.6 Hz), 167.3 (d, J = 17.5 Hz), 132.8 (d, J = 2.2 Hz), 130.1 (d, J = 2.4 Hz), 129.8 (d, J = 2.7 Hz), 128.1, 128.1, 127.5, 127.1, 126.8, 115.5 (d, J = 20.5 Hz), 61.1, 30.9 (d, J = 24.6 Hz), 22.6, 14.2. **^{19}F NMR** (471 MHz, $CDCl_3$): δ (ppm) -96.19 (t, J = 25.8 Hz, 1F). **HRMS** (ESI) m/z: Calcd for $C_{19}H_{20}FNO_2Na$ $[M+Na]^+$: 336.1370; Found 336.1375.

ethyl (*E*)-2-([1,1'-biphenyl]-4-yl)-3-fluoronon-2-enoate (3l)

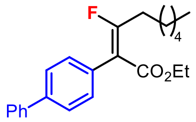

Following the general procedure (III), reaction was run using *gem*-difluoroalkene **1c** (57.6 mg, 0.2 mmol), hexyltrimethoxysilane **SR5** (123.8 mg, 0.6 mmol), $Pd(dba)_2$ (5.8 mg, 0.01 mmol) and dppe (4.0 mg, 0.01 mmol) in 1.0 mL toluene for 12 h. The product was purified by flash column chromatography on silica gel (DCM : hexane = 1 : 10) and obtained as a colorless oil (63,7 mg, 90% yield), R_f = 0.35 (DCM : hexane = 1 : 4). **^{1}H NMR** (500 MHz, $CDCl_3$): δ (ppm) 7.84 - 7.56 (m, 4H), 7.46 (t, J = 7.7 Hz, 2H), 7.36 (d, J = 8.1 Hz, 3H), 4.27 (q, J = 7.1 Hz, 2H), 2.83 (dt, J = 26.0, 7.6 Hz, 2H), 1.72 (tt, J = 7.8, 6.5 Hz, 2H), 1.50 - 1.42 (m, 2H), 1.36 (ddd, J = 7.1, 5.1, 2.7 Hz, 4H), 1.29 (t, J = 7.1 Hz, 3H), 1.01 - 0.83 (m, 3H). **^{13}C NMR** (126 MHz, $CDCl_3$): δ (ppm) 170.2 (d, J = 272.3 Hz), 167.4 (d, J = 17.5 Hz), 140.9, 140.4, 131.8, 130.2 (d, J = 2.8 Hz), 128.9, 127.5, 127.3, 126.9, 115.3 (d, J = 19.9 Hz), 61.2, 31.7, 31.3 (d, J = 24.8 Hz), 29.0, 26.6, 22.7, 14.3, 14.2. **^{19}F NMR** (471 MHz, $CDCl_3$): δ (ppm) -81.44 (t, J = 25.9 Hz, 1F). **HRMS** (ESI) m/z: Calcd for $C_{23}H_{27}FO_2Na$ $[M+Na]^+$: 377.1889; Found 377.1892.

ethyl (*E*)-3-fluoro-2-(4-methoxyphenyl)non-2-enoate (3m)

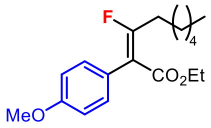

Following the general procedure (III), reaction was run using *gem*-difluoroalkene **1o** (48.6 mg, 0.2 mmol), hexyltrimethoxysilane **SR5** (123.8 mg, 0.6 mmol), $Pd(dba)_2$ (5.8 mg, 0.01 mmol) and dppe (4.0 mg, 0.01 mmol) in 1.0 mL toluene for 12 h. The product was purified by flash column chromatography on silica gel (DCM : hexane = 1 : 10) and obtained as a colorless oil (53.6 mg, 87% yield), R_f = 0.41 (DCM : hexane = 1 : 1). **^{1}H NMR** (500 MHz, $CDCl_3$): δ (ppm) 7.18 (d, J = 8.6 Hz, 2H), 6.88 (d, J = 8.7 Hz, 2H), 4.21 (q, J = 7.1 Hz, 2H), 3.81 (s, 3H), 2.76 (dt, J = 25.9, 7.6 Hz, 2H), 1.66 (p, J = 7.4 Hz, 2H), 1.49 - 1.37 (m, 2H), 1.32 (dt, J = 7.3, 3.7 Hz, 4H), 1.25 (t, J = 7.1 Hz, 3H), 0.90 (t, J = 6.9 Hz, 3H). **^{13}C NMR** (126 MHz, $CDCl_3$): δ (ppm) 169.6 (d, J = 271.0 Hz), 167.7 (d, J = 17.2 Hz), 158.9, 131.0 (d, J = 2.9 Hz), 125.1 (d, J = 2.1 Hz), 115.1 (d, J = 20.0 Hz), 113.6, 61.1, 55.3, 31.7, 31.2 (d, J = 24.8 Hz), 29.0, 26.6, 22.7, 14.3, 14.2. **^{19}F NMR** (471 MHz, $CDCl_3$): δ (ppm) -82.69 (t, J = 25.9 Hz, 1F). **HRMS** (ESI) m/z: Calcd for $C_{18}H_{25}FO_3Na$ $[M+Na]^+$: 331.1688; Found 331.1684.

ethyl (*E*)-3-fluoro-2-(4-(trifluoromethyl)phenyl)non-2-enoate (3n)

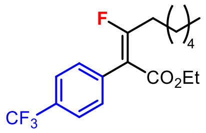

Following the general procedure (III), reaction was run using *gem*-difluoroalkene **1d** (56.6 mg, 0.2 mmol), hexyltrimethoxysilane **SR5** (123.8 mg, 0.6 mmol), $Pd(dba)_2$ (5.8 mg, 0.01 mmol) and dppe (4.0 mg, 0.01 mmol) in 1.0 mL toluene for 12 h. The product was purified by flash column chromatography on silica gel (DCM : hexane = 1 : 10) and obtained as a colorless oil (29.1 mg, 43% yield), R_f = 0.39 (DCM : hexane = 1 : 4). **^{1}H NMR** (500 MHz, $CDCl_3$): δ (ppm) 7.63 (d, J = 8.1 Hz, 2H), 7.39 (d, J = 8.0 Hz, 2H), 4.23 (q, J = 7.1 Hz, 2H), 2.85 (dt, J = 26.2, 7.6 Hz, 2H), 1.70 (ddt, J = 9.0, 7.6, 3.8 Hz, 2H), 1.50 - 1.40 (m, 2H), 1.35 (ddt, J = 7.1, 3.9, 2.4 Hz, 4H), 1.30 - 1.19 (m, 3H), 0.96 - 0.90 (m, 3H). **^{13}C NMR** (126 MHz, $CDCl_3$): δ (ppm) 171.4 (d, J = 274.2 Hz), 166.5 (d, J = 17.3 Hz), 136.5, 130.2 (d, J = 2.7 Hz), 130.0 - 129.1 (m), 124.9 (q, J = 3.8 Hz), 123.1, 61.2, 31.5, 31.0 (d, J = 24.1 Hz), 29.7, 28.9, 26.4, 22.5, 14.1, 14.1. **^{19}F NMR** (471 MHz, $CDCl_3$): δ (ppm) -62.60 (s, 3F), -78.37 (t, J = 26.3 Hz, 1F). **HRMS** (ESI) m/z: Calcd for $C_{18}H_{22}F_4O_2Na$ $[M+Na]^+$: 369.1455; Found 369.1453.

ethyl (*E*)-3-fluoro-2-(2-fluorophenyl)non-2-enoate (3o)

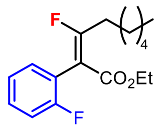

Following the general procedure (III), reaction was run using *gem*-difluoroalkene **1e** (46.6 mg, 0.2 mmol), hexyltrimethoxysilane **SR5** (123.8 mg, 0.6 mmol), Pd(dba)$_2$ (5.8 mg, 0.01 mmol) and dppe (4.0 mg, 0.01 mmol) in 1.0 mL toluene for 12 h. The product was purified by flash column chromatography on silica gel (DCM : hexane = 1 : 10) and obtained as a colorless oil (44.1 mg, 74% yield), R_f = 0.33 (DCM : hexane = 1 : 4). **^{1}H NMR** (500 MHz, CDCl$_3$): δ (ppm) 7.24 (dd, J = 8.5, 5.5 Hz, 2H), 7.06 (t, J = 8.7 Hz, 2H), 4.23 (q, J = 7.1 Hz, 2H), 2.82 (dt, J = 26.1, 7.6 Hz, 2H), 1.69 (p, J = 7.4 Hz, 2H), 1.52 – 1.40 (m, 2H), 1.40 – 1.32 (m, 4H), 1.26 (t, J = 7.1 Hz, 3H), 1.04 – 0.85 (m, 3H). **^{13}C NMR** (126 MHz, CDCl$_3$): δ (ppm) 170.6 (d, J = 272.4 Hz), 167.2 (d, J = 17.2 Hz), 162.2 (d, J = 246.6 Hz), 131.6 (dd, J = 8.2, 2.7 Hz), 115.1 (d, J = 21.5 Hz), 61.2, 31.6, 31.2 (d, J = 24.5 Hz), 29.0, 26.6, 22.7, 14.2, 14.2. **^{19}F NMR** (471 MHz, CDCl$_3$): δ (ppm) -80.27 (J = 26.3 Hz, 1F), -114.64 (q, J = 7.8, 7.0 Hz, 1F). **HRMS** (ESI) m/z: Calcd for $C_{17}H_{22}F_2O_2Na$ [M+Na]$^+$: 319.1485; Found 319.1486.

ethyl (*E*)-3-fluoro-2-(4-fluorophenyl)non-2-enoate (3p)

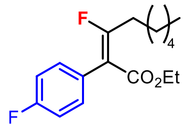

Following the general procedure (III), reaction was run using *gem*-difluoroalkene **1g** (46.6 mg, 0.2 mmol), hexyltrimethoxysilane **SR5** (123.8 mg, 0.6 mmol), Pd(dba)$_2$ (5.8 mg, 0.01 mmol) and dppe (4.0 mg, 0.01 mmol) in 1.0 mL toluene for 12 h. The product was purified by flash column chromatography on silica gel (DCM : hexane = 1 : 10) and obtained as a colorless oil (59.1 mg, 83% yield), R_f = 0.39 (DCM : hexane = 1 : 4). **^{1}H NMR** (500 MHz, CDCl$_3$): δ (ppm) 7.21 (dd, J = 8.5, 5.5 Hz, 2H), 7.03 (t, J = 8.7 Hz, 2H), 4.20 (q, J = 7.1 Hz, 2H), 2.79 (dt, J = 26.1, 7.6 Hz, 2H), 1.67 (p, J = 7.4 Hz, 2H), 1.48 - 1.38 (m, 2H), 1.36 - 1.29 (m, 4H), 1.24 (t, J = 7.1 Hz, 3H), 0.98 - 0.86 (m, 3H). **^{13}C NMR** (126 MHz, CDCl$_3$): δ (ppm) 170.6 (d, J = 272.4 Hz), 167.2 (d, J = 17.2 Hz), 162.2 (d, J = 246.6 Hz), 131.6 (dd, J = 8.2, 2.7 Hz), 115.1 (d, J = 21.5 Hz), 61.2, 31.6, 31.2 (d, J = 24.5 Hz), 29.0, 26.6, 22.7, 14.2, 14.2. **^{19}F NMR** (471 MHz, CDCl$_3$): δ (ppm) -79.16 (J = 26.2 Hz, 1F), -112.24 - -129.92 (m, 1F). **HRMS** (ESI) m/z: Calcd for $C_{17}H_{22}F_2O_2Na$ [M+Na]$^+$: 319.1480; Found 319.1486.

benzyl (*E*)-3-fluoro-2-(4-fluorophenyl)non-2-enoate (3q)

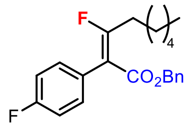

Following the general procedure (III), reaction was run using *gem*-difluoroalkene **1g** (58.4 mg, 0.2 mmol), hexyltrimethoxysilane **SR5** (123.8 mg, 0.6 mmol), Pd(dba)$_2$ (5.8 mg, 0.01 mmol) and dppe (4.0 mg, 0.01 mmol) in 1.0 mL toluene for 12 h. The product was purified by flash column chromatography on silica gel (DCM : hexane = 1 : 10) and obtained as a colorless oil (59.1 mg, 78% yield), R_f = 0.29 (DCM : hexane = 1 : 4). **^{1}H NMR** (500 MHz, CDCl$_3$): δ (ppm) 7.34 (td, J = 5.2, 2.3 Hz, 3H), 7.31 - 7.19 (m, 4H), 7.09 - 6.99 (m, 2H), 5.21 (s, 2H), 2.78 (dd, J = 26.1, 7.7 Hz, 2H), 1.80 - 1.51 (m, 2H), 1.44 - 1.19 (m, 6H), 0.91 (t, J = 6.7 Hz, 3H). **^{13}C NMR** (126 MHz, CDCl$_3$): δ (ppm) 171.2 (d, J = 273.1 Hz), 166.8 (d, J = 17.2 Hz), 162.3 (d, J = 246.7 Hz), 135.8, 131.7 (dd, J = 8.1, 2.5 Hz), 128.7, 128.4, 128.1, 115.1 (d, J = 21.5 Hz), 66.8, 31.6, 31.2 (d, J = 24.3 Hz), 29.0, 26.6, 22.6, 14.2. **^{19}F NMR** (471 MHz, CDCl$_3$): δ (ppm) -78.67 (t, J = 26.1 Hz, 1F), -107.38 - -118.82 (m,1F). **HRMS** (ESI) m/z: Calcd for $C_{22}H_{24}F_2O_2Na$ [M+Na]$^+$: 381.1645; Found 381.1641.

isopropyl (*E*)-3-fluoro-2-(4-fluorophenyl)non-2-enoate (3r)

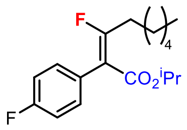

Following the general procedure (III), reaction was run using *gem*-difluoroalkene **1p** (28.8 mg, 0.2 mmol), hexyltrimethoxysilane **SR5** (123.8 mg, 0.6 mmol), $Pd(dba)_2$ (5.8 mg, 0.01 mmol) and dppe (4.0 mg, 0.01 mmol) in 1.0 mL toluene for 12 h. The product was purified by flash column chromatography on silica gel (DCM : hexane = 1 : 10) and obtained as a colorless oil (49.1 mg, 80% yield), R_f = 0.41 (DCM : hexane = 1 : 4). ^{1}**H NMR** (500 MHz, $CDCl_3$): δ (ppm) 7.27 - 7.21 (m, 2H), 7.14 - 6.98 (m, 2H), 5.12 (h, J = 6.3 Hz, 1H), 2.79 (dt, J = 26.1, 7.6 Hz, 2H), 1.73 - 1.63 (m, 2H), 1.47 - 1.39 (m, 2H), 1.35 (ddd, J = 7.2, 5.0, 2.8 Hz, 4H), 1.26 (d, J = 6.3 Hz, 6H), 0.99 - 0.85 (m, 3H). ^{13}**C NMR** (126 MHz, $CDCl_3$): δ (ppm) 169.8 (d, J = 272.0 Hz), 166.3 (d, J = 246.7 Hz), 162.2 (d, J = 246.2 Hz), 131.5 (dd, J = 8.1, 2.7 Hz), 115.0 (d, J = 21.6 Hz), 68.8, 31.7, 31.2 (d, J = 24.8 Hz), 29.0, 26.6, 22.7, 21.9, 14.2. ^{19}**F NMR** (471 MHz, $CDCl_3$): δ (ppm) -82.22 (t, J = 26.0 Hz, 1F), -114.77 (m, 1F). **HRMS** Calcd for $C_{18}H_{24}F_2O_2Na$ (ESI) m/z: $[M+Na]^+$: 333.1543; Found 333.1542.

ethyl (*E*)-3-fluoro-2-(thiophen-3-yl)non-2-enoate (3s)

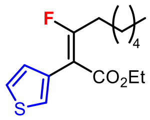

Following the general procedure (III), reaction was run using *gem*-difluoroalkene **1q** (43.6 mg, 0.2 mmol), hexyltrimethoxysilane **SR5** (123.8 mg, 0.6 mmol), $Pd(dba)_2$ (5.8 mg, 0.01 mmol) and dppe (4.0 mg, 0.01 mmol) in 1.0 mL toluene for 12 h. The product was purified by flash column chromatography on silica gel (DCM : hexane = 1 : 10) and obtained as a colorless oil (41.5 mg, 73% yield), R_f = 0.39 (DCM : hexane = 1 : 2). ^{1}**H NMR** (500 MHz, $CDCl_3$): δ (ppm) 7.40 - 7.34 (m, 1H), 7.33 - 7.27 (m, 1H), 7.15 (dt, J = 5.1, 1.4 Hz, 1H), 4.29 (q, J = 7.2 Hz, 2H), 2.71 (dt, J = 25.7, 7.6 Hz, 2H), 1.68 (p, J = 7.5 Hz, 2H), 1.41 (ddt, J = 13.7, 8.4, 4.5 Hz, 2H), 1.38 - 1.23 (m, 7H), 1.01 - 0.85 (m, 3H). ^{13}**C NMR** (126 MHz, $CDCl_3$): δ (ppm) 168.5 (d, J = 260.7 Hz), 167.3 (d, J = 5.1 Hz), 131.8, 128.8 (d, J = 4.2 Hz), 127.0 – 120.9 (m), 111.0 (d, J = 18.8 Hz), 31.6, 31.4 (d, J = 24.8 Hz), 29.0, 26.6, 22.7, 14.2 (d, J = 13.2 Hz). ^{19}**F NMR** (471 MHz, $CDCl_3$): δ (ppm) -84.35 (t, J = 25.6 Hz, 1F). **HRMS** (ESI) m/z: Calcd for $C_{15}H_{21}FO_2SNa$ $[M+Na]^+$: 307.1140; Found 307.1143.

ethyl (*E*)-3-fluoro-2-(11-oxo-6,11-dihydrodibenzo[b,e]oxepin-2-yl)non-2-enoate (7)

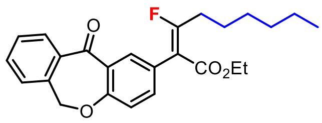

Following the general procedure (III), reaction was run using *gem*-difluoroalkene **6** (68.0 mg, 0.2 mmol), hexyltrimethoxysilane **SR5** (123.8 mg, 0.6 mmol), $Pd(dba)_2$ (5.8 mg, 0.01 mmol) and dppe (4.0 mg, 0.01 mmol) in 1.0 mL toluene for 12 h. The product was purified by flash column chromatography on silica gel (DCM : hexane = 1 : 1) and obtained as a colorless oil (66.5 mg, 81% yield), R_f = 0.35 (DCM : hexane = 1 : 1). ^{1}**H NMR** (500 MHz, $CDCl_3$): δ (ppm) 8.20 (dd, J = 2.4, 0.8 Hz, 1H), 7.89 (dd, J = 7.7, 1.5 Hz, 1H), 7.65 - 7.29 (m, 4H), 7.09 (d, J = 8.5 Hz, 1H), 5.22 (s, 2H), 4.29 (q, J = 7.1 Hz, 2H), 2.71 (dt, J = 25.7, 7.6 Hz, 2H), 1.68 (p, J = 7.5 Hz, 2H), 1.41 (ddt, J = 13.7, 8.4, 4.5 Hz, 2H), 1.38 - 1.23 (m, 7H), 1.31 (t, J = 7.1 Hz, 3H). ^{13}**C NMR** (126 MHz, $CDCl_3$): δ (ppm) 190.5, 166.1 (d, J = 517.3 Hz), 164.0 (d, J = 3.3 Hz), 163.1 (d, J = 218.1 Hz), 161.1, 159.8 (d, J = 14.6 Hz), 140.4, 136.8 (d, J = 2.5 Hz), 135.3, 134.4, 130.0, 127.9, 125.1, 123.0 (d, J = 2.4 Hz), 121.0 (d, J = 3.2 Hz), 91.3 (d, J =11.2 Hz), 60.7, 52.1, 31.6, 30.9 (d, J = 24.7 Hz), 29.0, 28.9, 26.7, 22.7, 14.2. ^{19}**F NMR** (471 MHz, $CDCl_3$): δ (ppm) -80.93 (t, J = 25.5 Hz, 1F). **HRMS** (ESI) m/z: Calcd for $C_{25}H_{27}FO_4Na$ $[M+Na]^+$: 433.0866; Found 433.0863.

(*E*)-3-fluoro-1-morpholino-2-phenylnon-2-en-1-one (9)

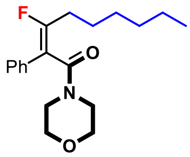

Following the general procedure (III), reaction was run using *gem*-difluoroalkene **8** (50.6 mg, 0.2 mmol), hexyltrimethoxysilane **SR5** (123.8 mg, 0.6 mmol), $Pd(dba)_2$ (5.8 mg, 0.01 mmol) and dppe (4.0 mg, 0.01 mmol) in 1.0 mL toluene for 12 h. The product was purified by flash column chromatography on silica gel (DCM : hexane = 1 : 1) and obtained as a colorless oil (40.3 mg, 63% yield), R_f = 0.40 (DCM : hexane = 1 : 1). **^{1}H NMR** (500 MHz, $CDCl_3$): δ (ppm) 7.88 - 6.86 (m, 5H), 3.72 (dd, *J* = 20.1, 5.2 Hz, 4H), 3.40 (dd, *J* = 16.3, 5.0 Hz, 4H), 2.42 (dt, *J* = 22.6, 7.6 Hz, 2H), 1.65 (m, 4H), 1.52 – 1.31 (m, 4H), 0.92 (t, *J* = 6.8 Hz, 3H). **^{13}C NMR** (126 MHz, $CDCl_3$): δ (ppm) 167.1 (d, *J* = 13.7 Hz), 160.3 (d, *J* = 270.3 Hz), 132.1, 128.8, 128.1 (d, *J* = 5.8 Hz), 127.9, 115.7 (d, *J* = 12.8 Hz), 66.8 (d, *J* = 6.8 Hz), 31.7, 31.4 (d, *J* = 26.3 Hz), 29.8, 29.1, 26.4, 22.7, 14.2. **^{19}F NMR** (471 MHz, $CDCl_3$): δ (ppm) -104.49 (t, *J* = 25.4 Hz, 1F). **HRMS** (ESI) m/z: Calcd for $C_{19}H_{26}FNO_2Na$ $[M+Na]^+$: 342.1846; Found 342.1845.

benzyl (*E*)-3-fluoronon-2-enoate (11)

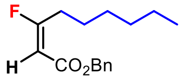

Following the general procedure (III), reaction was run using *gem*-difluoroalkene **10** (39.6 mg, 0.2 mmol), hexyltrimethoxysilane **SR5** (123.8 mg, 0.6 mmol), $Pd(dba)_2$ (5.8 mg, 0.01 mmol) and dppe (4.0 mg, 0.01 mmol) in 1.0 mL toluene for 12 h. The product was purified by flash column chromatography on silica gel (DCM : hexane = 1 : 1) and obtained as a colorless oil (40.3 mg, 74% yield), R_f = 0.36 (DCM : hexane = 1 : 5). **^{1}H NMR** (500 MHz, $CDCl_3$): δ (ppm) 7.56 - 7.31 (m, 5H), 5.64 (d, *J* = 19.5 Hz, 1H), 5.18 (s, 2H), 2.83 (dt, *J* = 25.8, 7.6 Hz, 2H), 1.77 - 1.54 (m, 2H), 1.43 - 1.19 (m, 6H), 1.03 - 0.84 (m, 3H). **^{13}C NMR** (126 MHz, $CDCl_3$): δ (ppm) 178.0 (d, *J* = 275.2 Hz), 166.2 (d, *J* = 27.0 Hz), 136.0, 128.7, 128.4, 128.3, 100.8 (d, *J* = 29.9 Hz), 66.2, 31.6, 29.9 (d, *J* = 22.6 Hz), 28.8, 26.1, 22.6, 14.2. **^{19}F NMR** (471 MHz, $CDCl_3$): δ (ppm) -74.4 (td, *J* = 45.8, 25.3 Hz, 1F). **HRMS** (ESI) m/z: Calcd for $C_{16}H_{21}FO_2Na$ $[M+Na]^+$: 287.1425; Found 287.1423.

VII. References.

1. Hu, M.; Ni, C.; Li, L.; Han, Y.; Hu, J. *gem*-Difluoroolefination of Diazo Compounds with $TMSCF_3$ or $TMSCF_2Br$: Transition-Metal-Free Cross-Coupling of Two Carbene Precursors. *J. Am. Chem. Soc.* **2015**, *137*, 14496-14501.
2. Ma, Q.; Wang, Y.; Tsui, G. C. Stereoselective Palladium-Catalyzed C-F Bond Alkynylation of Tetrasubstituted *gem*-Difluoroalkenes. *Angew. Chem., Int. Ed.* **2020**, *59*, 11293-11297.
3. Bauer, J. O.; Strohmann, C. Stereocontrol in Nucleophilic Substitution Reactions at Silicon: The Role of Permutation in Generating Silicon-Centered Chirality. *J. Am. Chem. Soc.* **2015**, *137*, 4304−4307.
4. Wang, Y.; Qi, X.; Ma, Q.; Liu, P.; Tsui, G. C. Stereoselective Palladium-Catalyzed Base-Free Suzuki-Miyaura Cross-Coupling of Tetrasubstituted *gem*-Difluoroalkenes: An Experimental and Computational Study. *ACS Catal.* **2021**, *11*, 4799-4809.
5. Li, M.; Wang, Y.; Tsui, G. C. Palladium-Catalyzed Stereoselective C−F Bond Vinylation and Allylation of Tetrasubstituted *gem*-Difluoroalkenes via Stille Coupling: Synthesis of Monofluorinated 1,3- and 1,4-Dienes. *Org. Lett.* **2021**, *23*, 8072-8076.
6. Wang, Y.; Tang, Y.; Zong, Y.; Tsui, G. C. Highly Selective C-F Bond Functionalization of Tetrasubstituted *gem*-Difluoroalkenes and Trisubstituted Monofluoroalkenes Using Grignard Reagents. *Org. Lett.* **2022**, *24*, 4087-4092.

VIII. Spectra

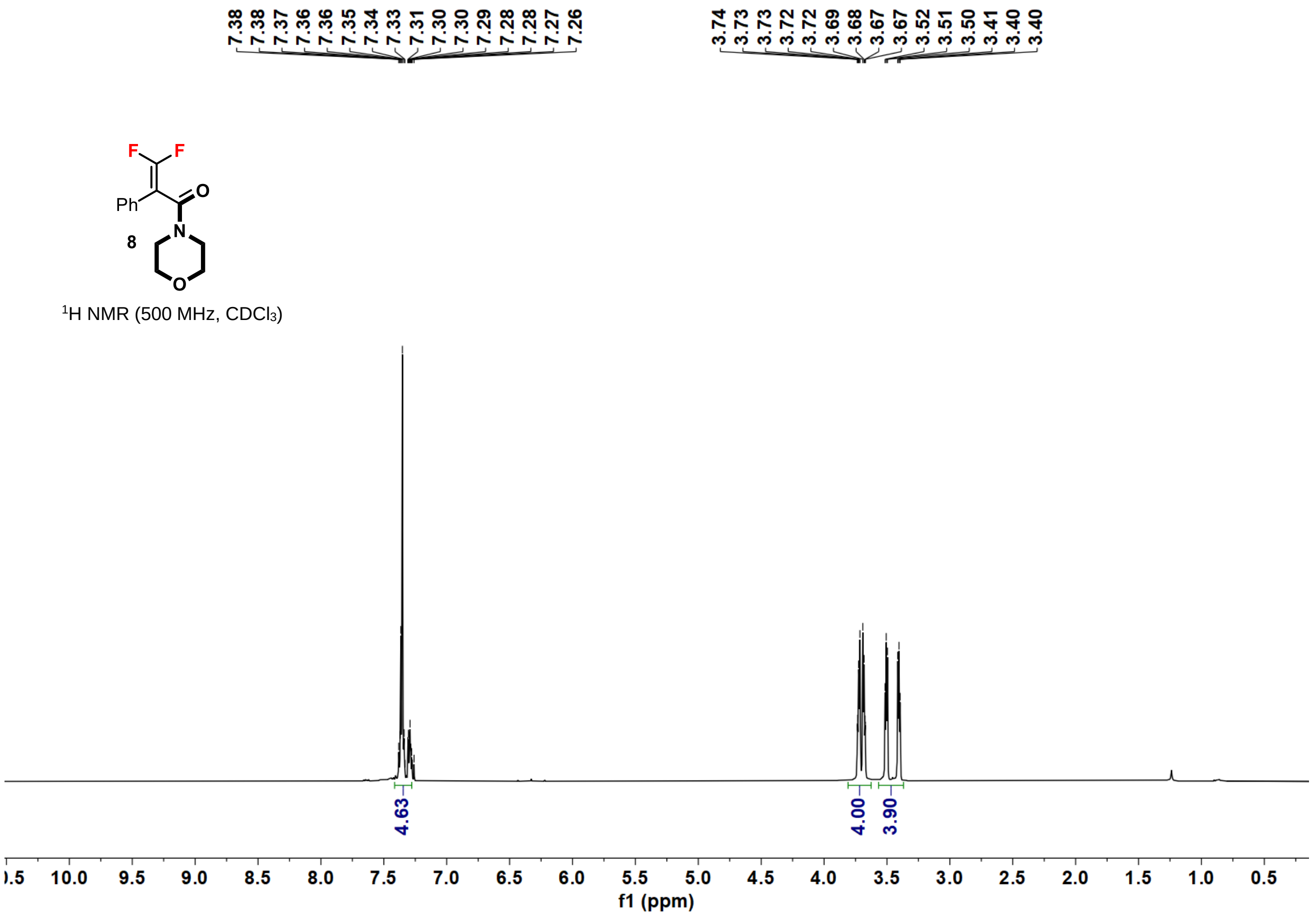

¹H NMR (500 MHz, CDCl₃)

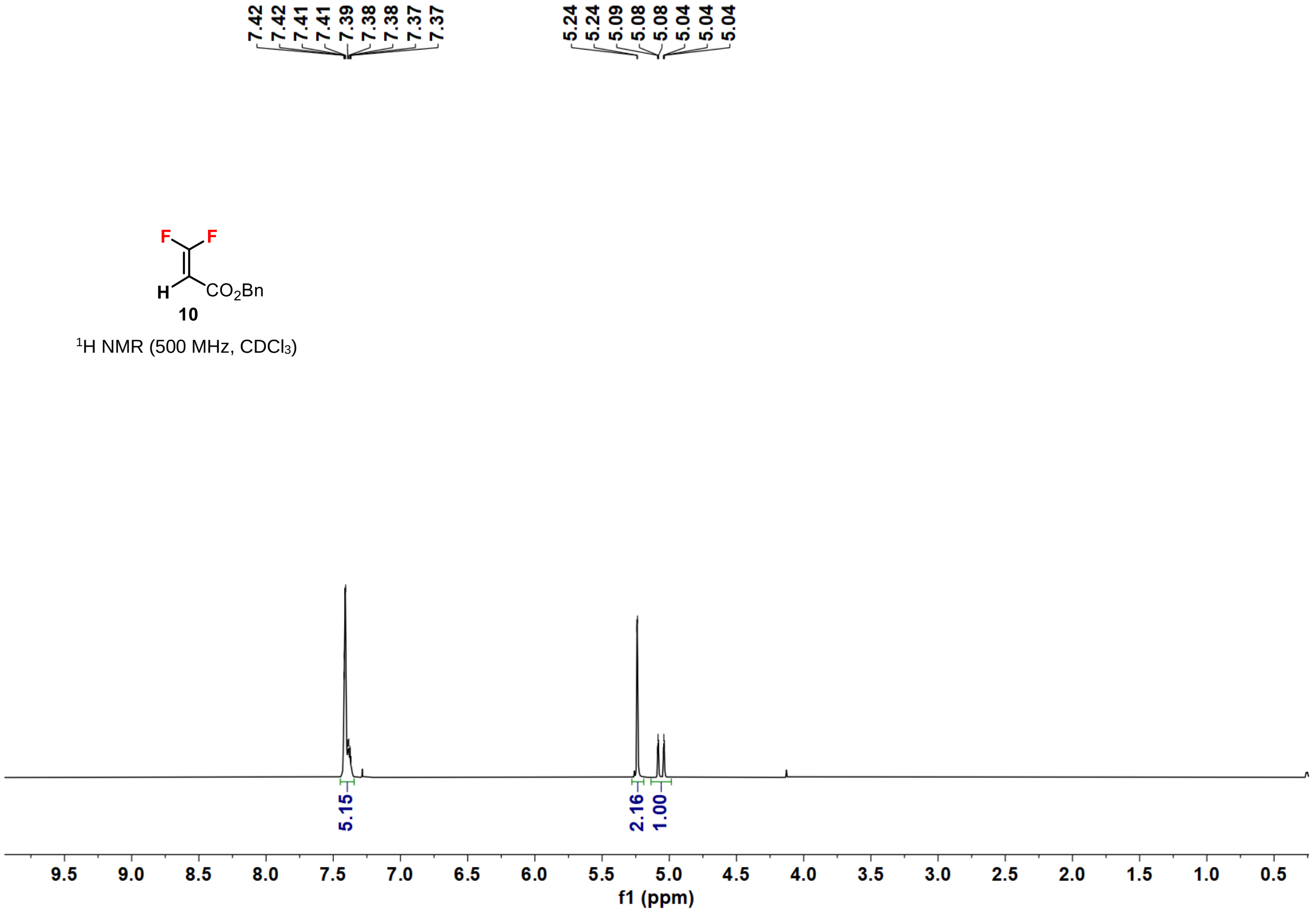

¹H NMR (500 MHz, CDCl₃)

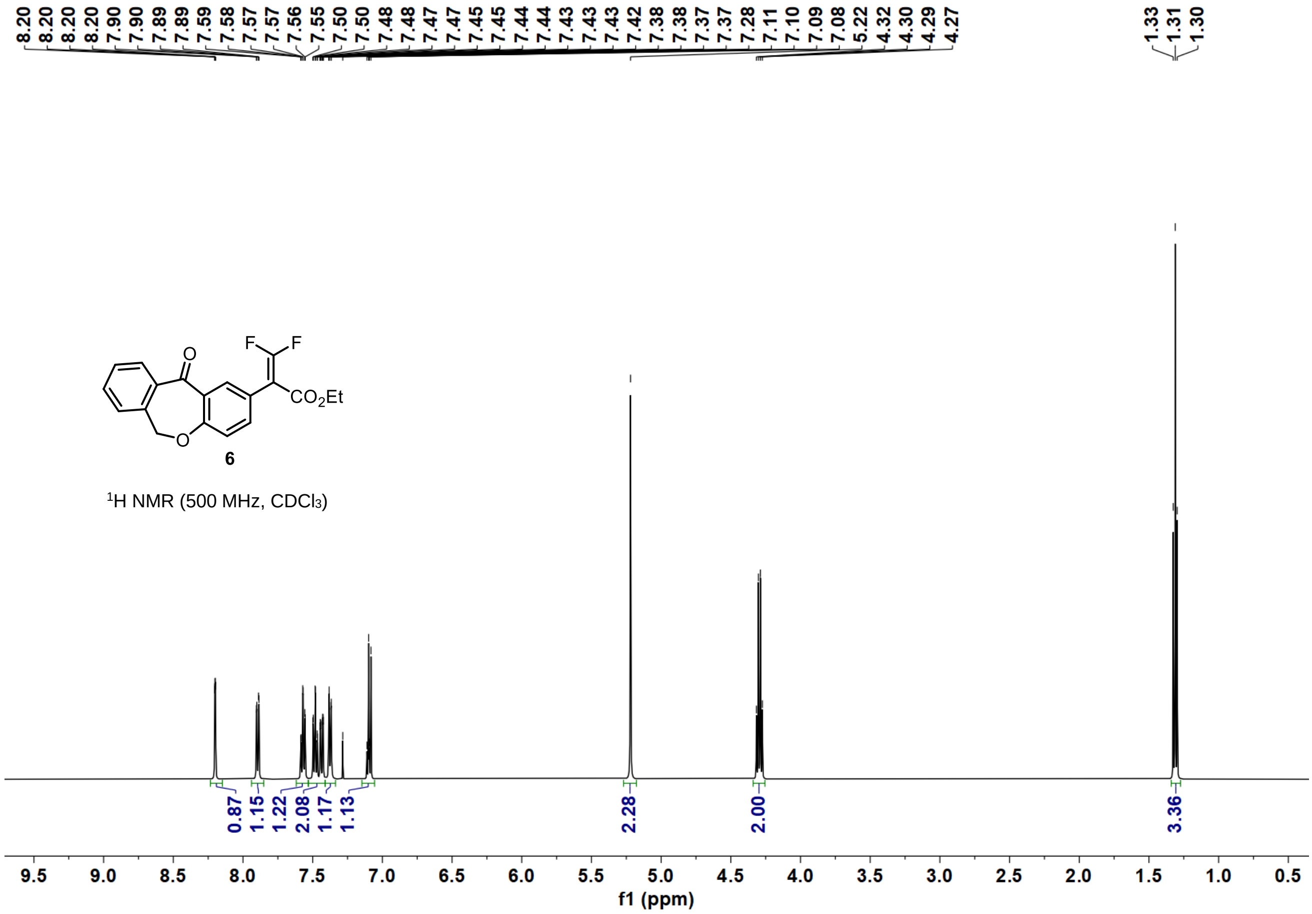
8.20 8.20 8.20 8.20 7.90 7.90 7.89 7.89 7.59 7.58 7.57 7.57 7.56 7.55 7.50 7.50 7.48 7.48 7.47 7.47 7.45 7.45 7.44 7.44 7.43 7.43 7.43 7.43 7.42 7.38 7.38 7.37 7.37 7.28 7.11 7.10 7.09 7.08 5.22 4.32 4.30 4.29 4.27
1.33 1.31 1.30
F
F
O
CO2Et
O
6
1H NMR (500 MHz, CDCl3)
0.87 1.15 1.22 2.08 1.17 1.13
2.28
2.00
3.36
9.5 9.0 8.5 8.0 7.5 7.0 6.5 6.0 5.5 5.0 4.5 4.0 3.5 3.0 2.5 2.0 1.5 1.0 0.5
f1 (ppm)

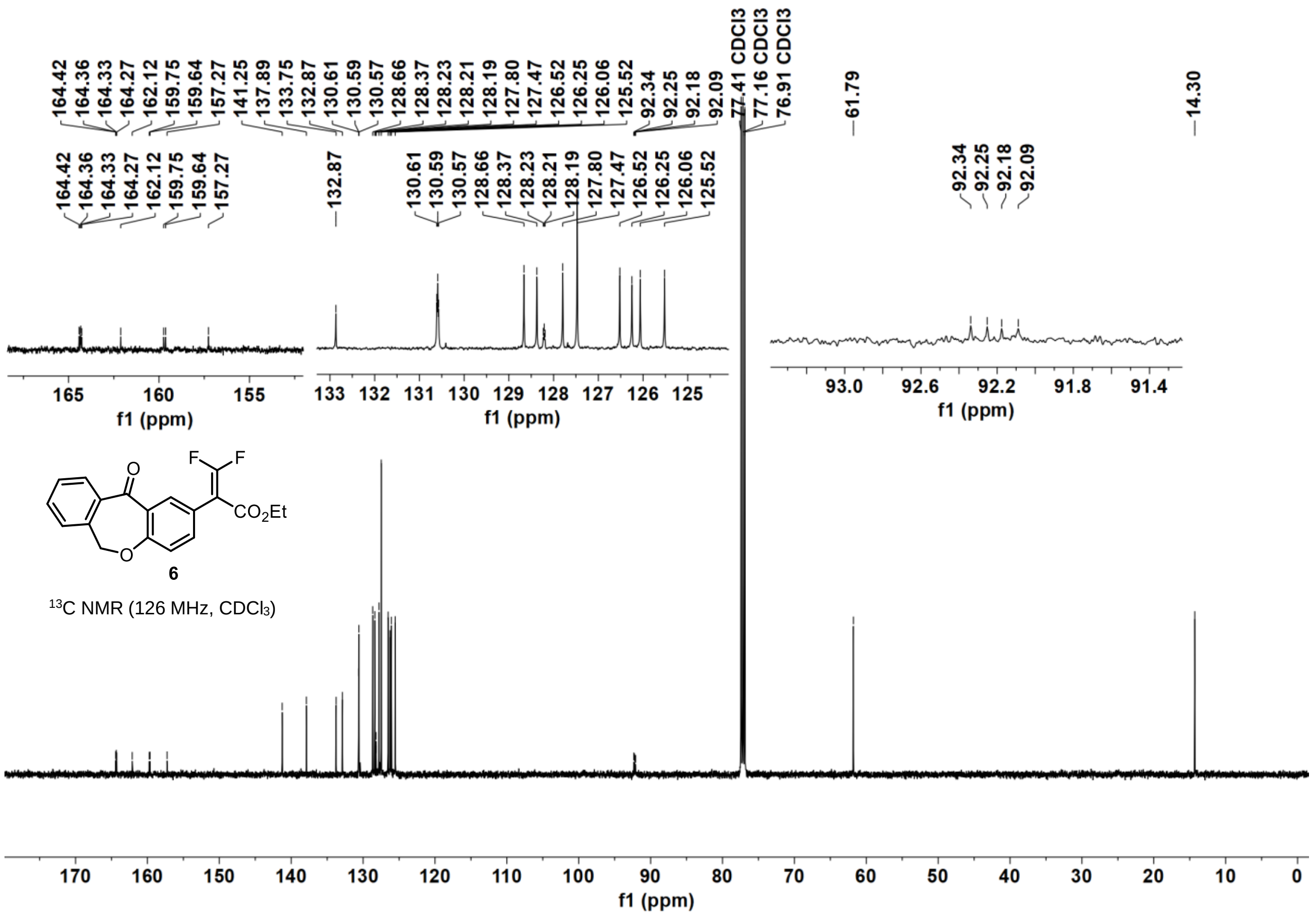
164.42 164.36 164.33 164.27 162.12 159.75 159.64 157.27 141.25 137.89 133.75 132.87 130.61 130.59 130.57 128.66 128.37 128.23 128.21 128.19 127.80 127.47 126.52 126.25 126.06 125.52 92.34 92.25 92.18 92.09
77.41 CDCl3
77.16 CDCl3
76.91 CDCl3
61.79
14.30
164.42 164.36 164.33 164.27 162.12 159.75 159.64 157.27
132.87
130.61 130.59 130.57 128.66 128.37 128.23 128.21 128.19 127.80 127.47 126.52 126.25 126.06 125.52
92.34 92.25 92.18 92.09
165 160 155
f1 (ppm)
133 132 131 130 129 128 127 126 125
f1 (ppm)
93.0 92.6 92.2 91.8 91.4
f1 (ppm)
F
F
O
CO2Et
O
6
13C NMR (126 MHz, CDCl3)
170 160 150 140 130 120 110 100 90 80 70 60 50 40 30 20 10 0
f1 (ppm)

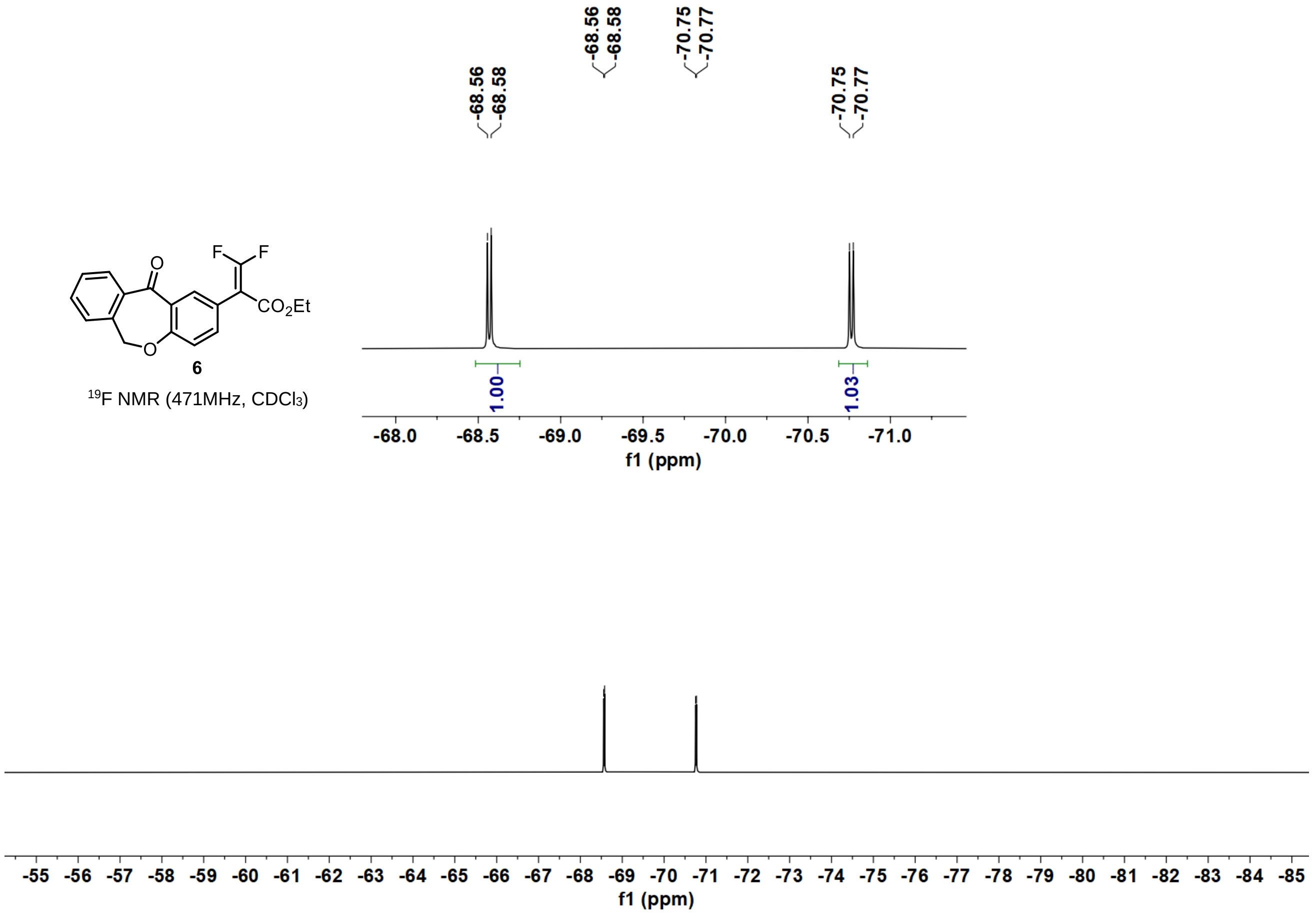
CO2Et
6
19F NMR (471MHz, CDCl3)
f1 (ppm)
f1 (ppm)

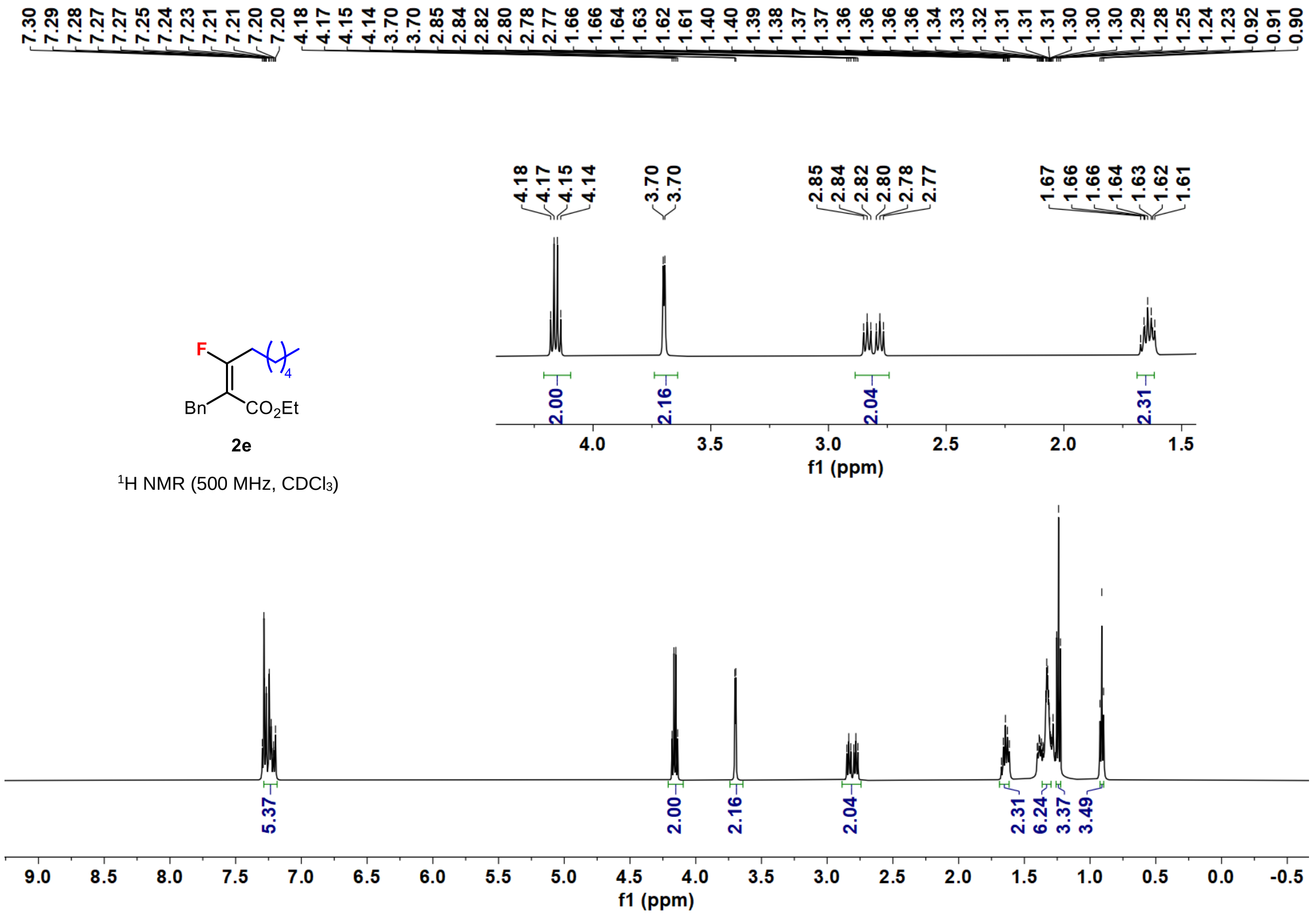
F
Bn
CO2Et
2e
1H NMR (500 MHz, CDCl3)
f1 (ppm)
f1 (ppm)

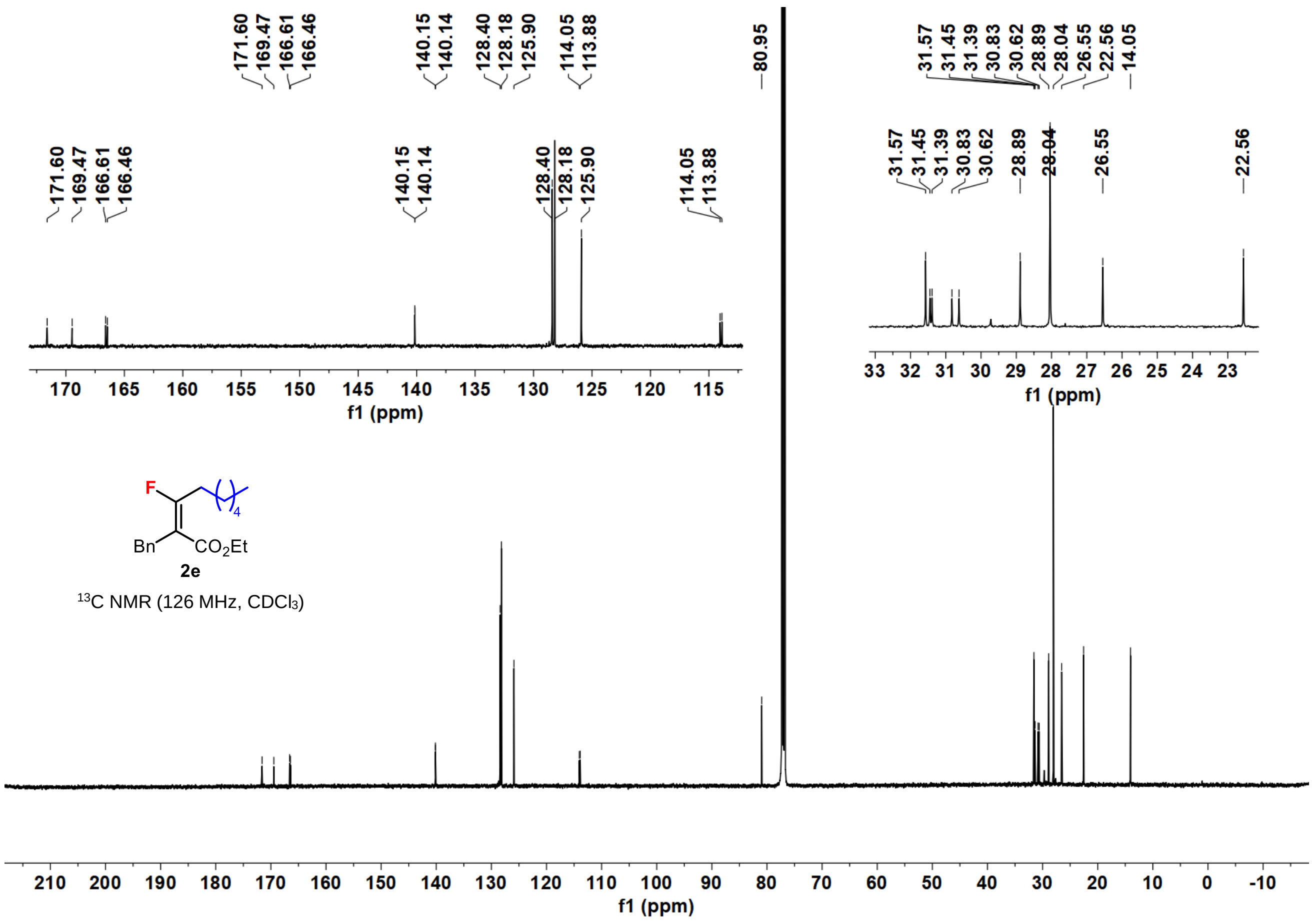
171.60
169.47
166.61
166.46
140.15
140.14
128.40
128.18
125.90
114.05
113.88
80.95
31.57
31.45
31.39
30.83
30.62
28.89
28.04
26.55
22.56
14.05
F
Bn
CO_2Et
4
2e
^{13}C NMR (126 MHz, $CDCl_3$)
f1 (ppm)

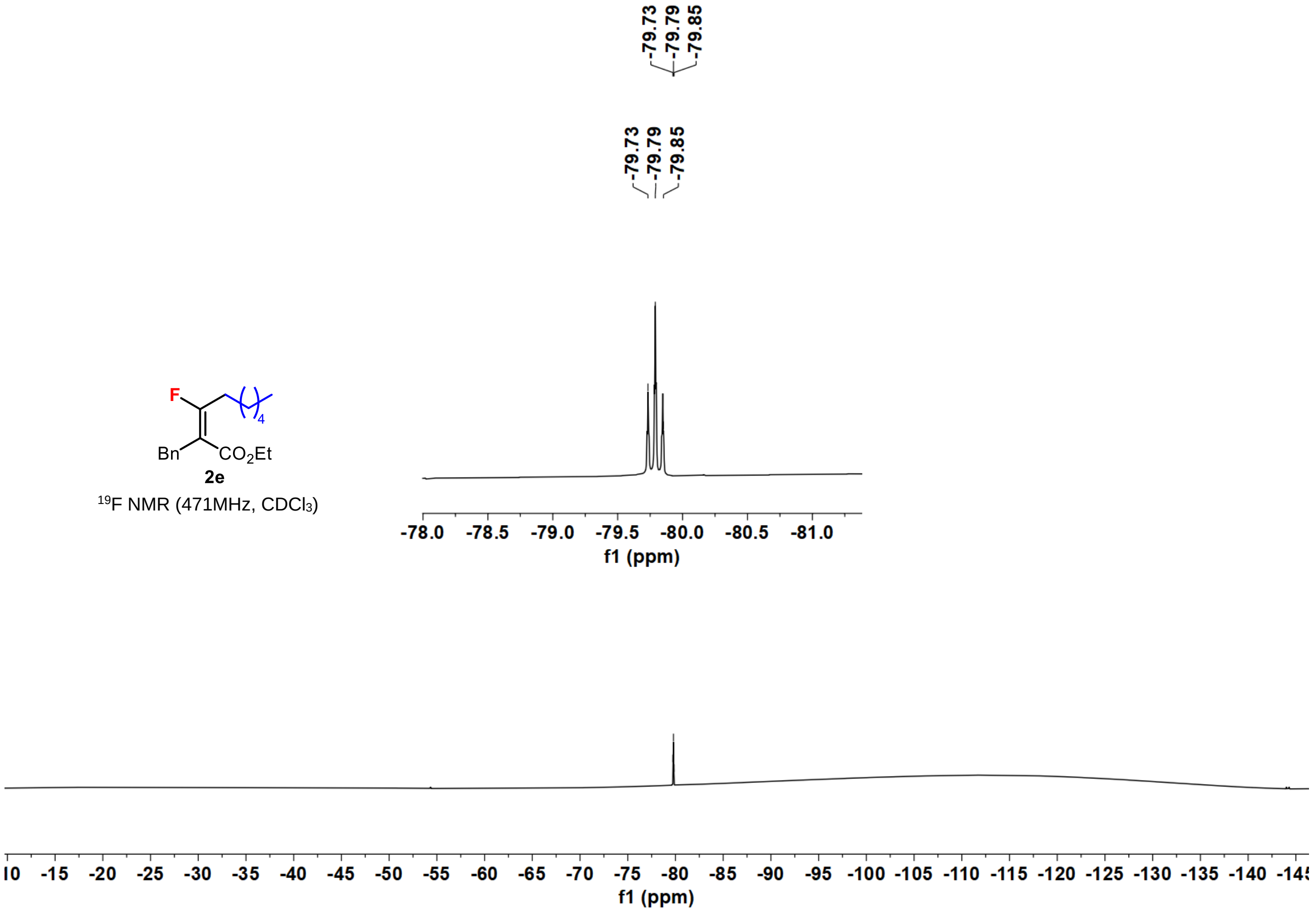
-79.73
-79.79
-79.85
F
Bn
CO_2Et
4
2e
^{19}F NMR (471MHz, $CDCl_3$)
f1 (ppm)

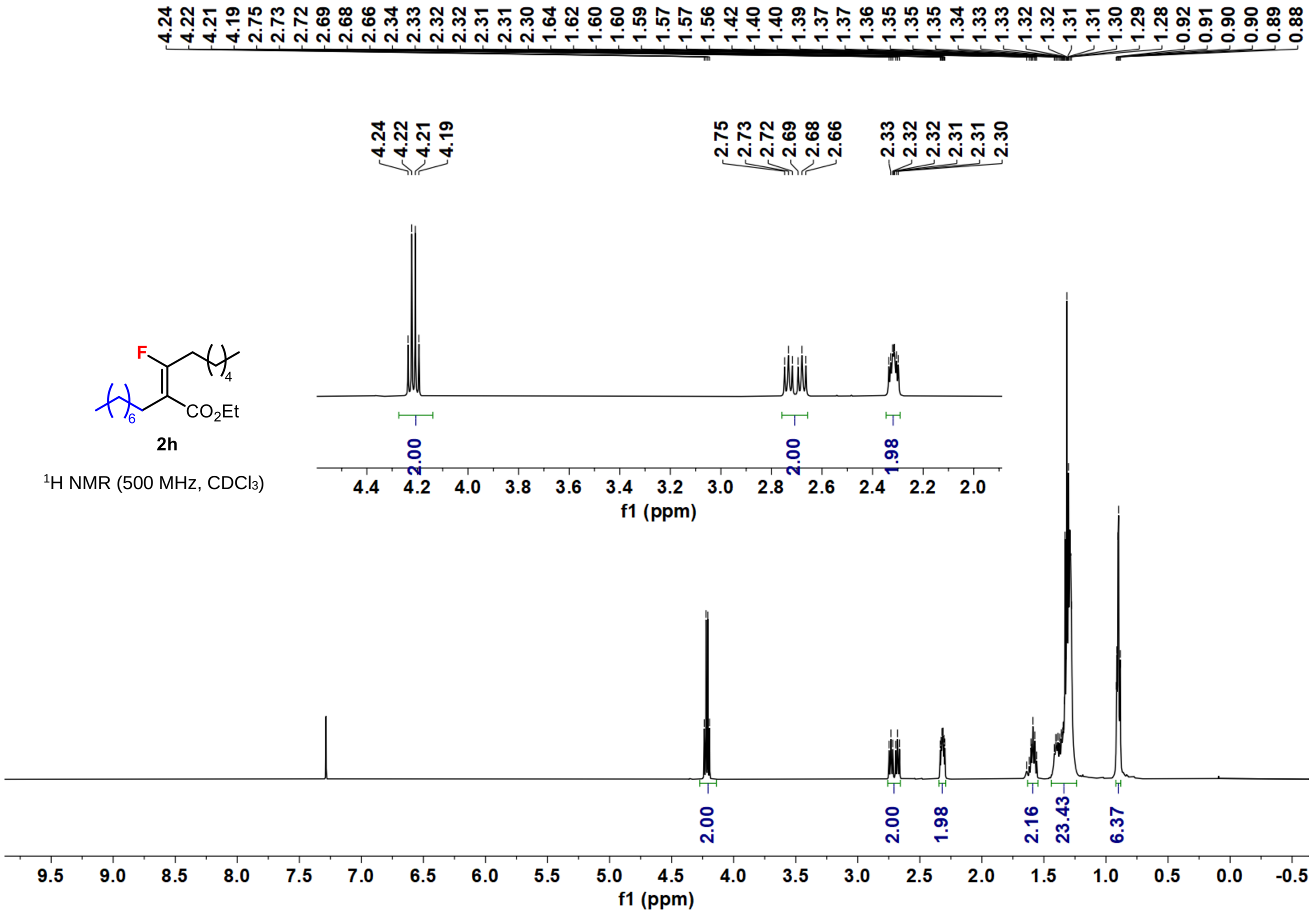

2h

^{1}H NMR (500 MHz, $CDCl_3$)

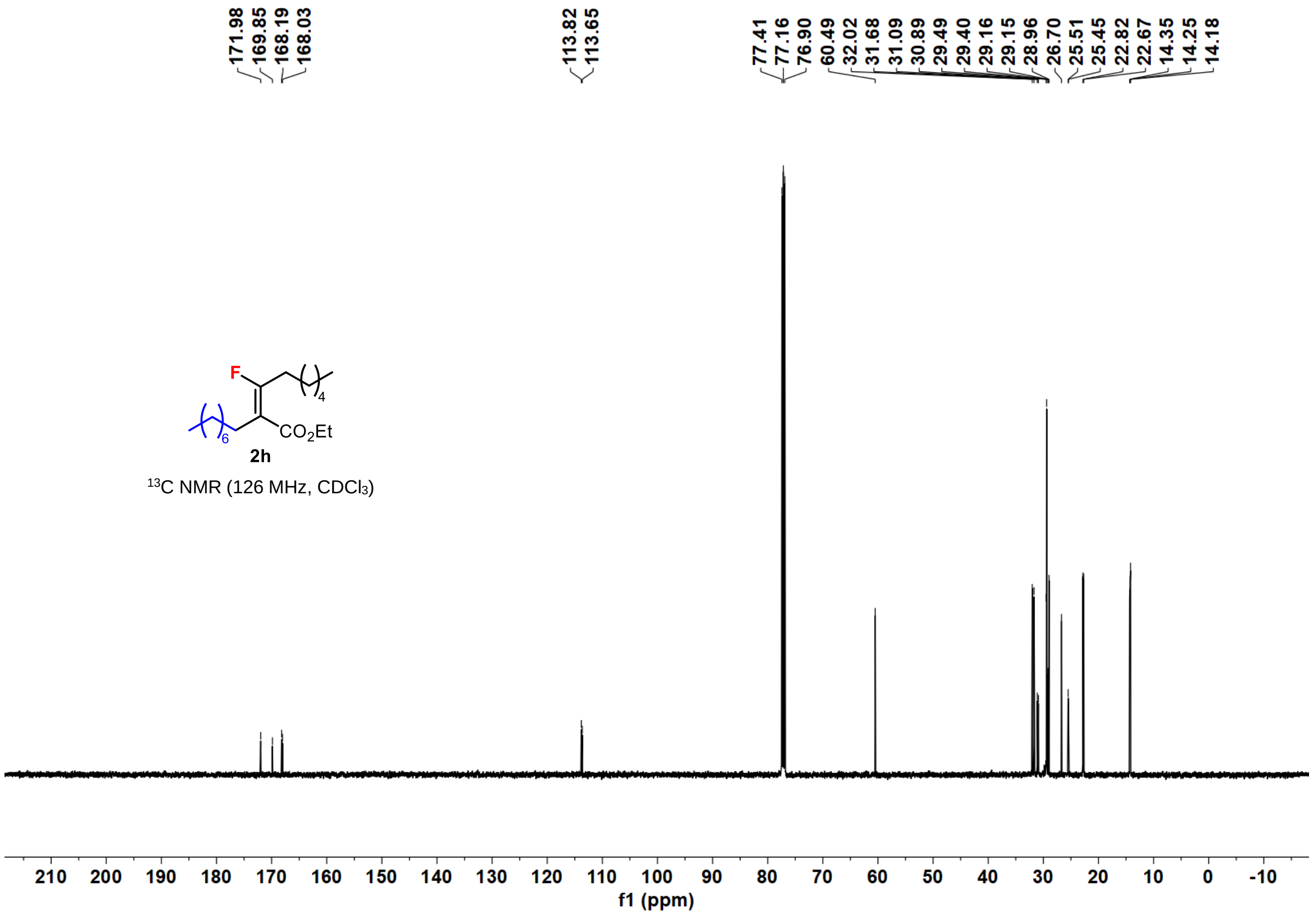

2h

^{13}C NMR (126 MHz, $CDCl_3$)

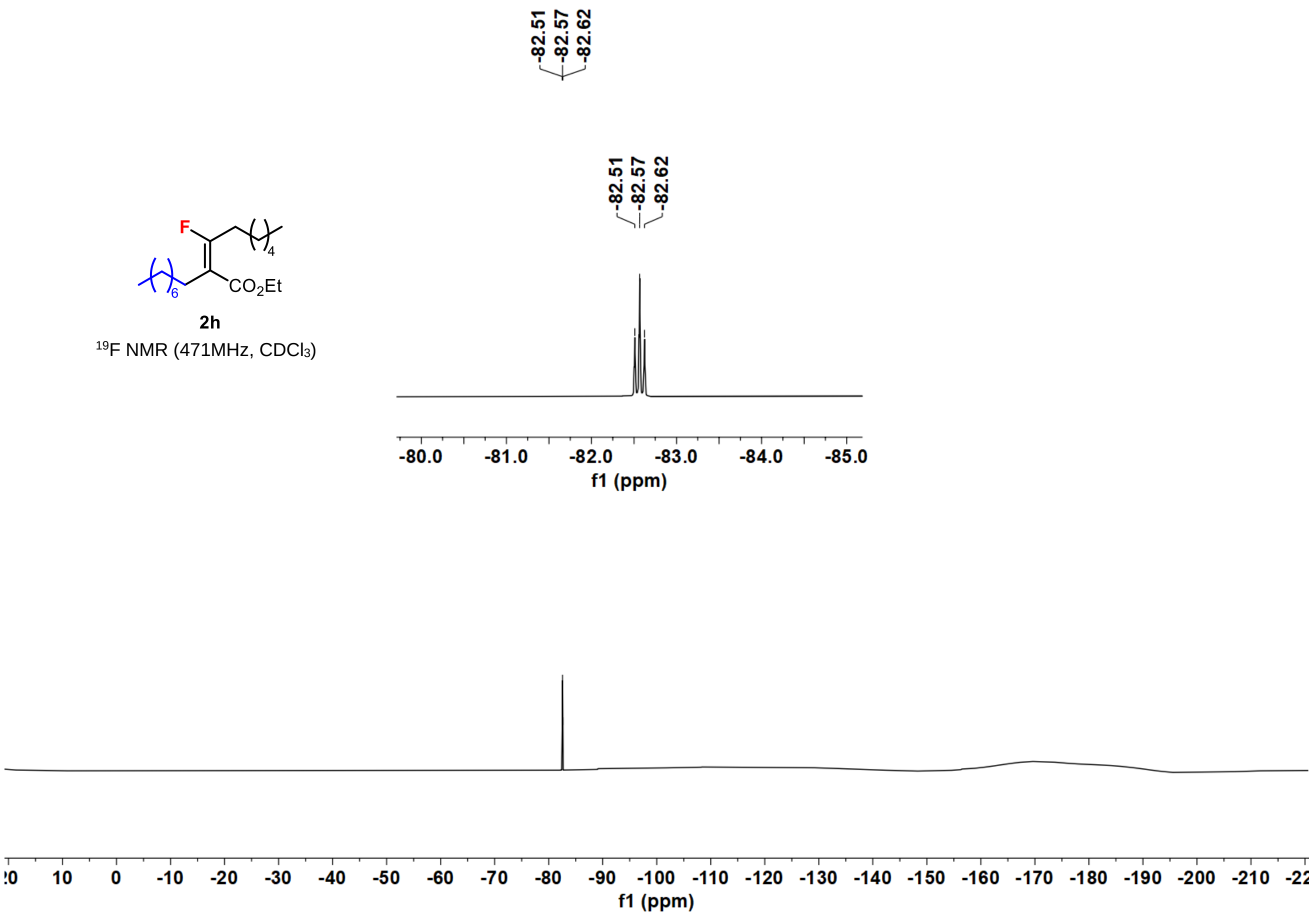
-82.51
-82.57
-82.62
F
4
6
CO_2Et
2h
^{19}F NMR (471MHz, $CDCl_3$)
-80.0 -81.0 -82.0 -83.0 -84.0 -85.0
f1 (ppm)
10 0 -10 -20 -30 -40 -50 -60 -70 -80 -90 -100 -110 -120 -130 -140 -150 -160 -170 -180 -190 -200 -210
f1 (ppm)

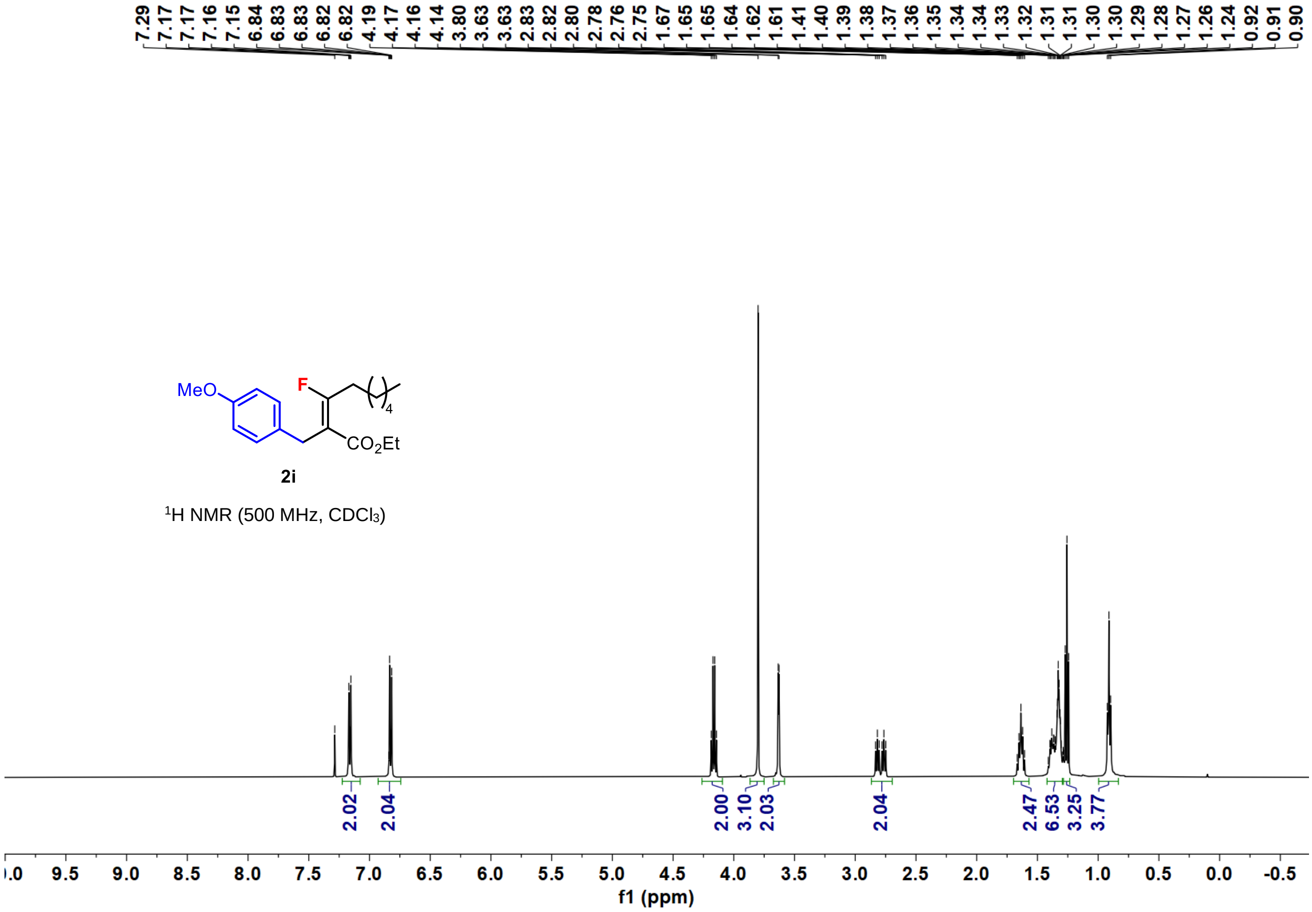
7.29 7.17 7.17 7.16 7.15 6.84 6.83 6.83 6.82 6.82 4.19 4.17 4.16 4.14 3.80 3.63 3.63 2.83 2.82 2.80 2.78 2.76 2.75 1.67 1.65 1.64 1.62 1.61 1.41 1.40 1.39 1.38 1.37 1.36 1.35 1.34 1.34 1.33 1.32 1.31 1.31 1.30 1.30 1.29 1.28 1.27 1.26 1.24 0.92 0.91 0.90
MeO
F
4
CO_2Et
2i
^1H NMR (500 MHz, $CDCl_3$)
2.02 2.04 2.00 3.10 2.03 2.04 2.47 6.53 3.25 3.77
9.5 9.0 8.5 8.0 7.5 7.0 6.5 6.0 5.5 5.0 4.5 4.0 3.5 3.0 2.5 2.0 1.5 1.0 0.5 0.0 -0.5
f1 (ppm)

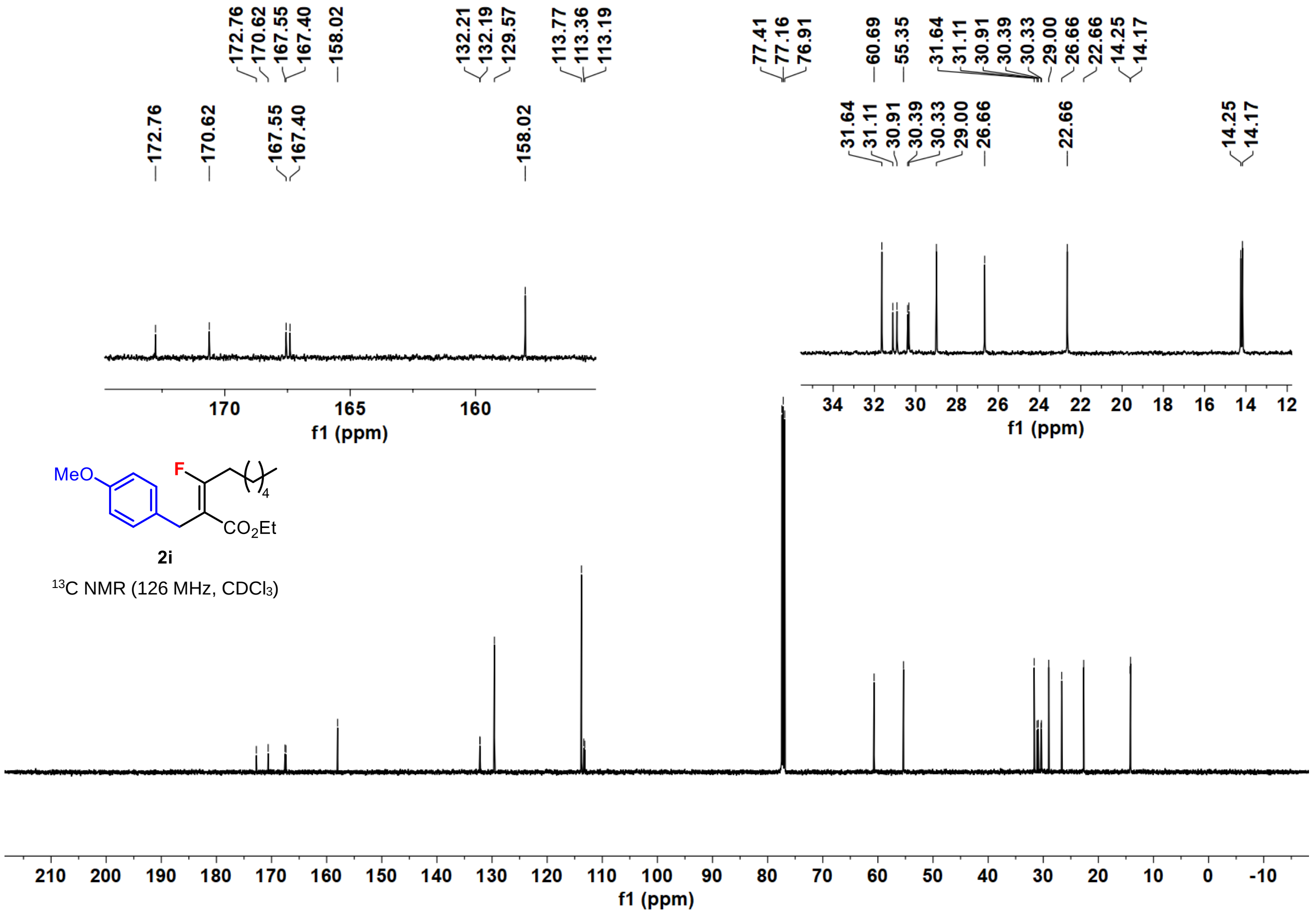
172.76
170.62
167.55
167.40
158.02
132.21
132.19
129.57
113.77
113.36
113.19
77.41
77.16
76.91
60.69
55.35
31.64
31.11
30.91
30.39
30.33
29.00
26.66
22.66
14.25
14.17
MeO
F
4
CO_2Et
2i
^{13}C NMR (126 MHz, $CDCl_3$)
f1 (ppm)

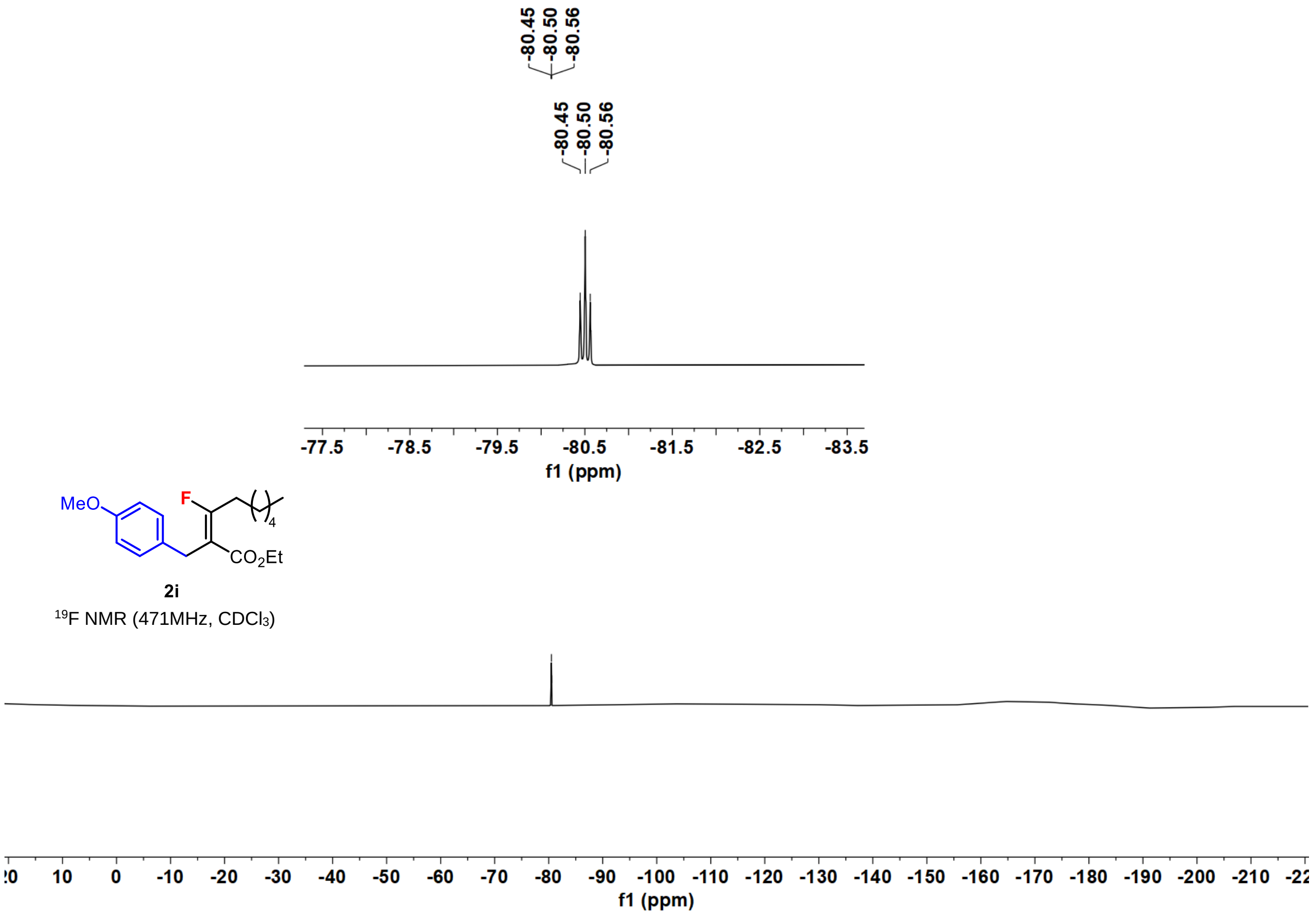
-80.45
-80.50
-80.56
MeO
F
4
CO_2Et
2i
^{19}F NMR (471MHz, $CDCl_3$)
f1 (ppm)

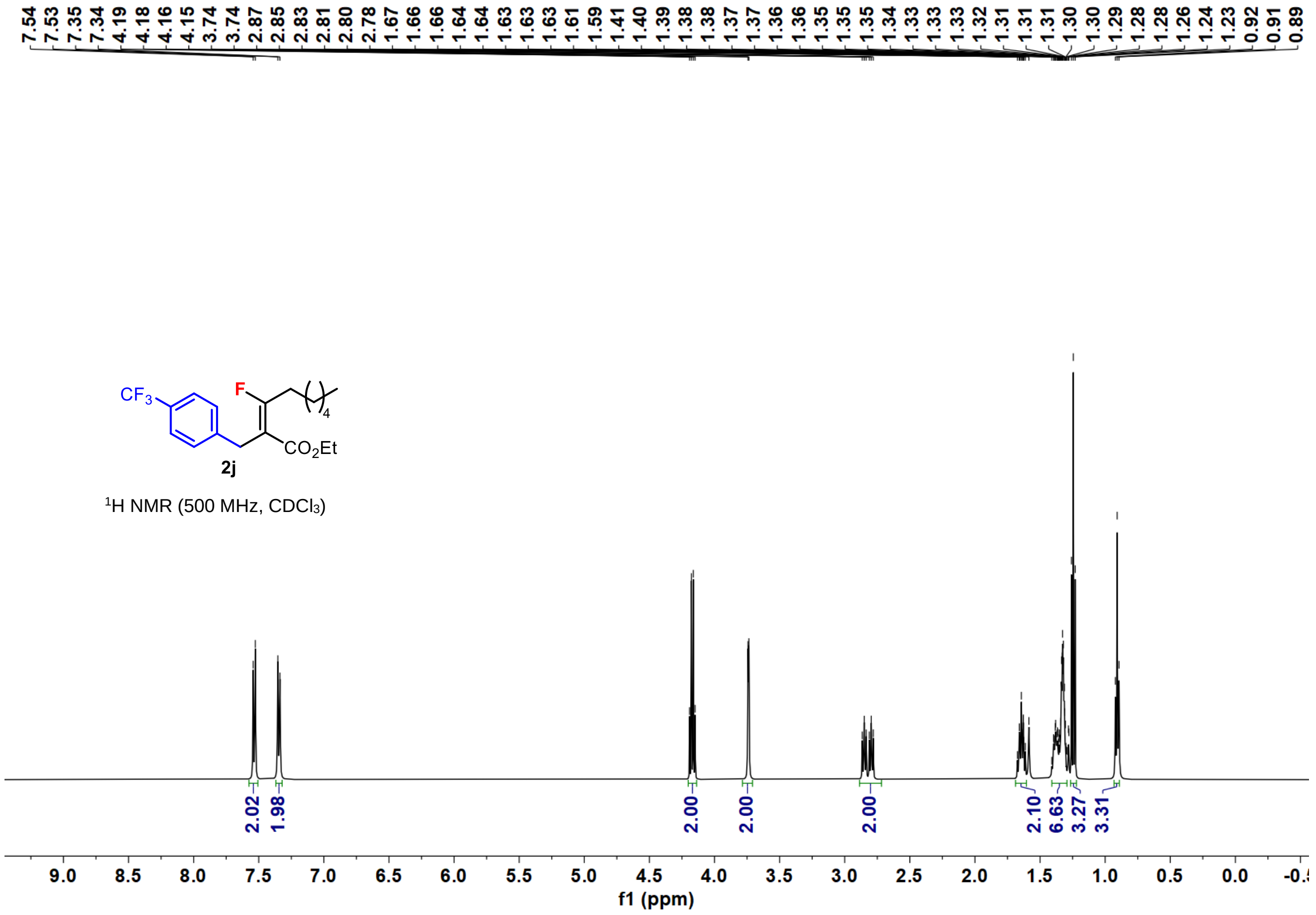
7.54 7.53 7.35 7.34 4.19 4.18 4.16 4.15 3.74 3.74 2.87 2.85 2.83 2.81 2.80 2.78 1.67 1.66 1.66 1.64 1.64 1.63 1.63 1.63 1.61 1.59 1.41 1.40 1.39 1.38 1.38 1.37 1.37 1.36 1.36 1.35 1.35 1.35 1.34 1.33 1.33 1.33 1.32 1.31 1.31 1.31 1.30 1.30 1.29 1.28 1.28 1.26 1.24 1.23 0.92 0.91 0.89
CF3
F
4
CO2Et
2j
1H NMR (500 MHz, CDCl3)
2.02 1.98 2.00 2.00 2.00 2.10 6.63 3.27 3.31
f1 (ppm)

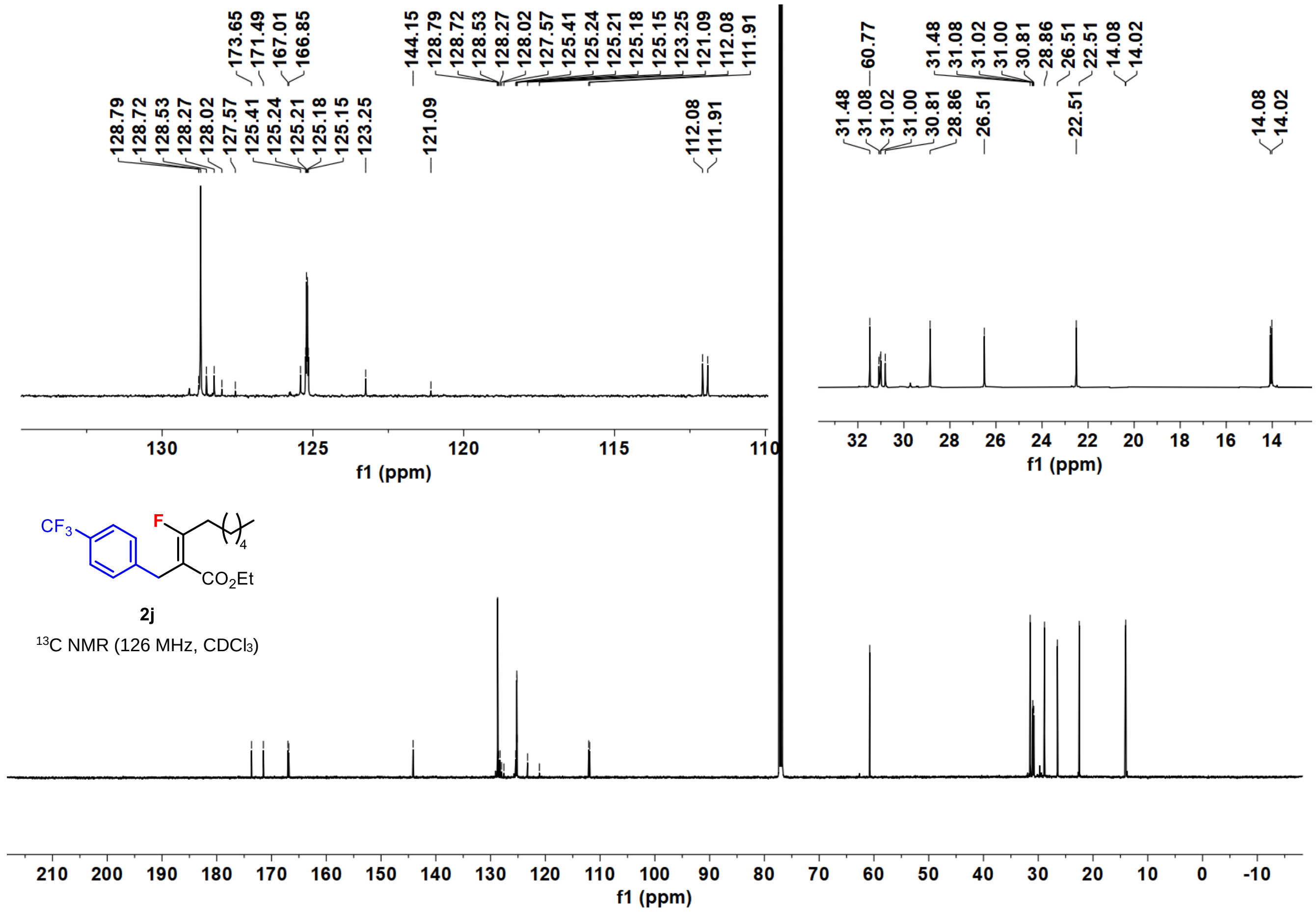
173.65 171.49 167.01 166.85 144.15 128.79 128.72 128.53 128.27 128.02 127.57 125.41 125.24 125.21 125.18 125.15 123.25 121.09 112.08 111.91
60.77 31.48 31.08 31.02 31.00 30.81 28.86 26.51 22.51 14.08 14.02
f1 (ppm)
CF3
F
4
CO2Et
2j
13C NMR (126 MHz, CDCl3)

2j

^{19}F NMR (471MHz, $CDCl_3$)

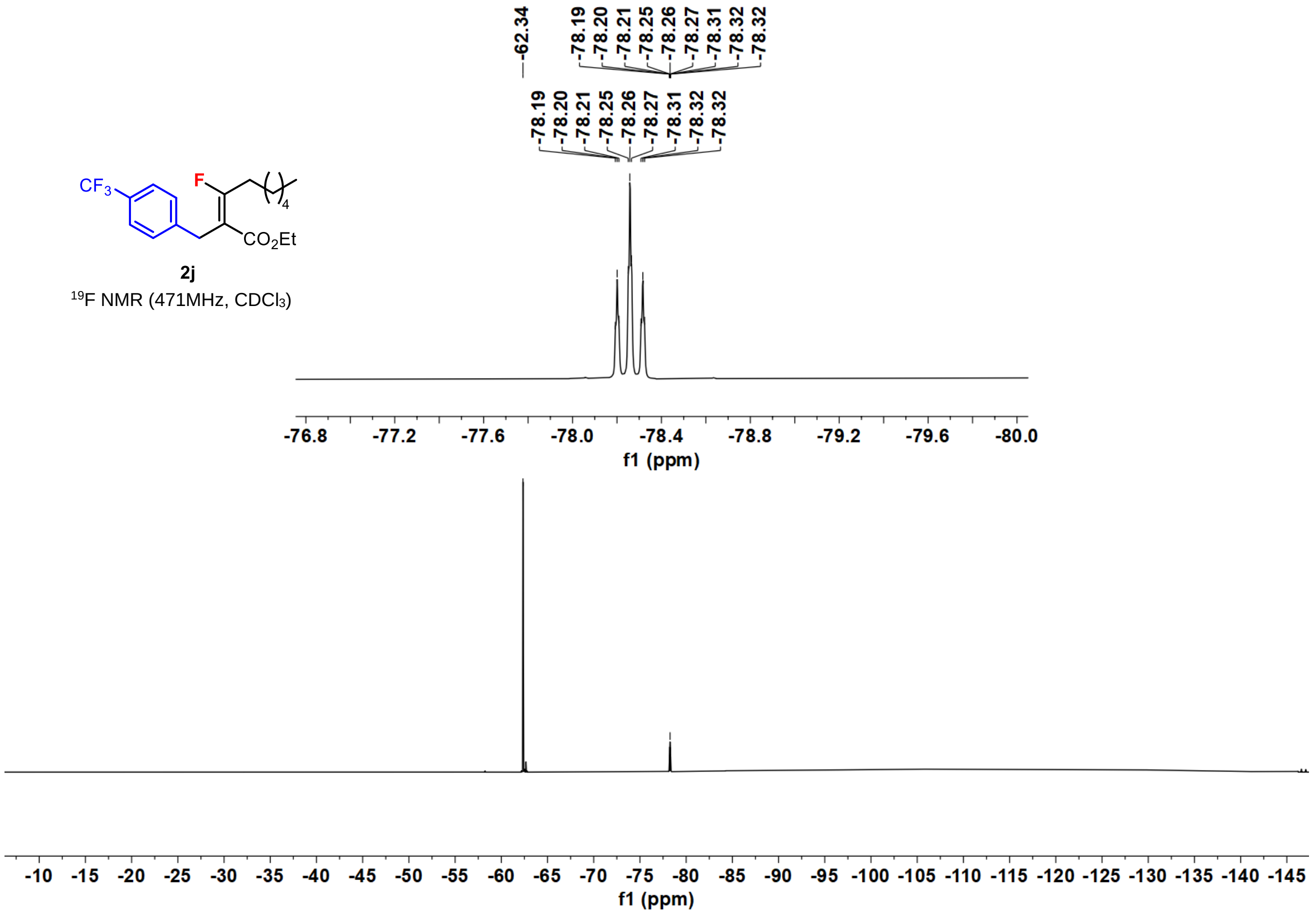

2k

^{1}H NMR (500 MHz, $CDCl_3$)

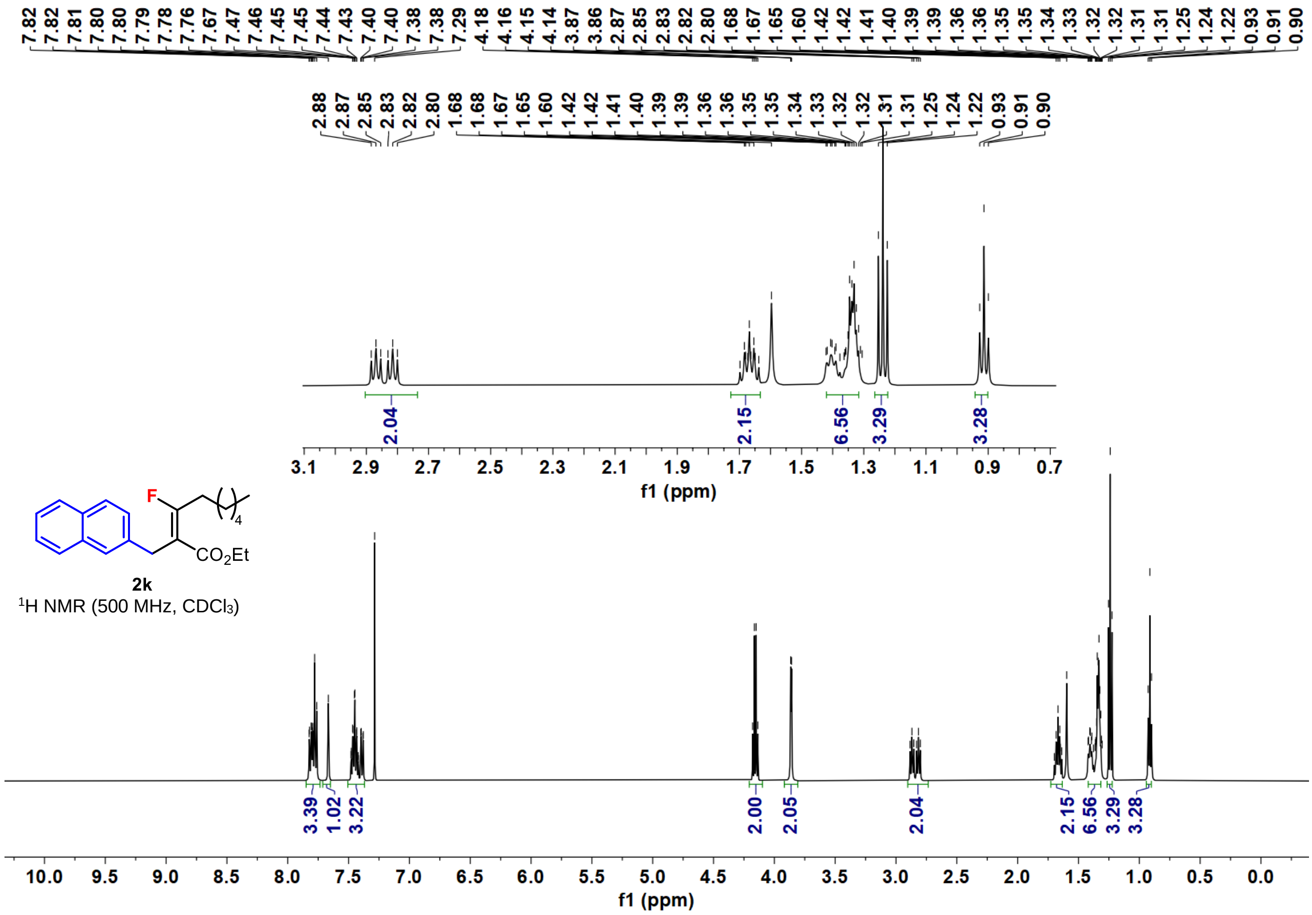

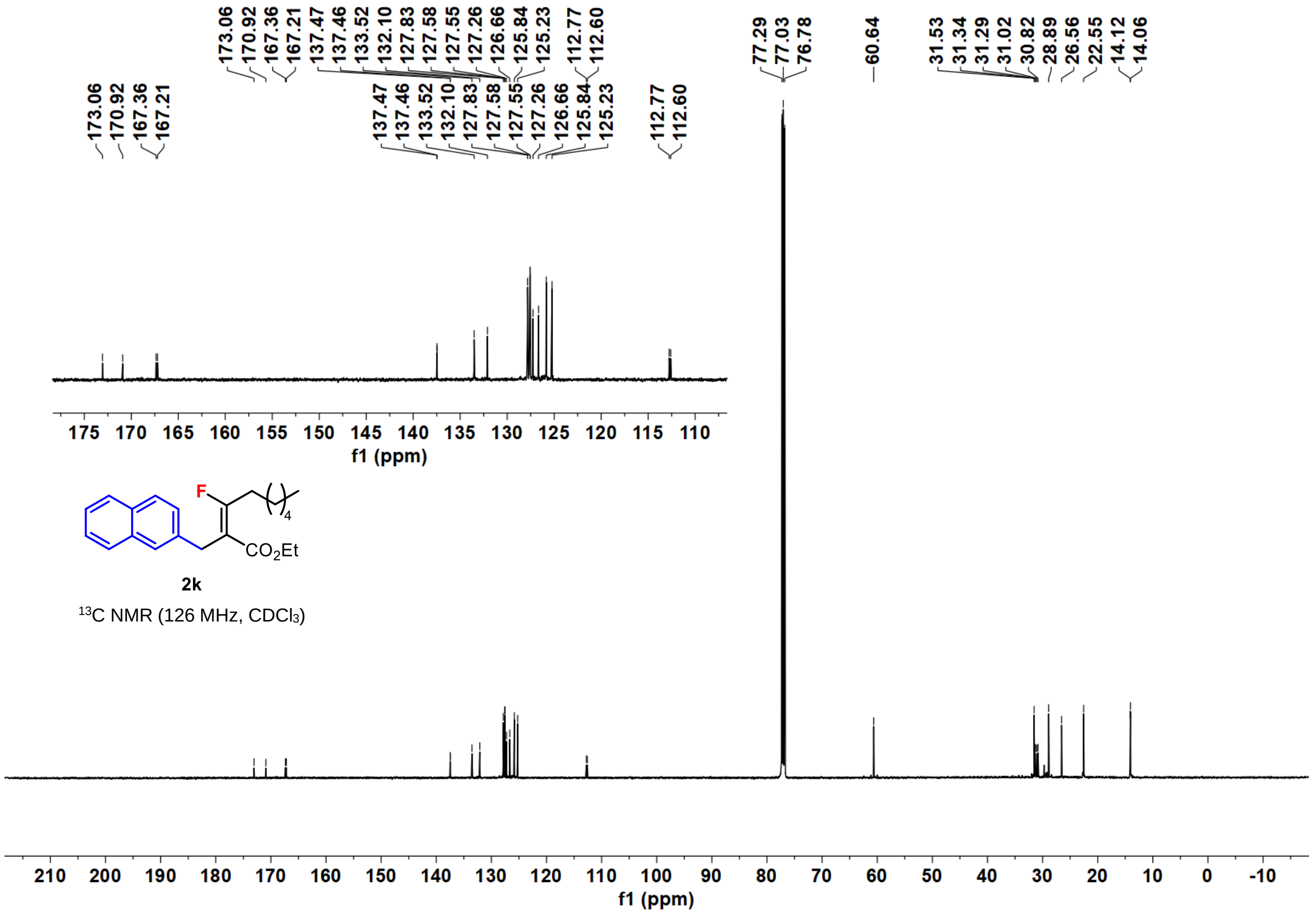
173.06
170.92
167.36
167.21
137.47
137.46
133.52
132.10
127.83
127.58
127.55
127.26
126.66
125.84
125.23
112.77
112.60
77.29
77.03
76.78
60.64
31.53
31.34
31.29
31.02
30.82
28.89
26.56
22.55
14.12
14.06
F
4
CO_2Et
2k
^{13}C NMR (126 MHz, $CDCl_3$)
f1 (ppm)
f1 (ppm)

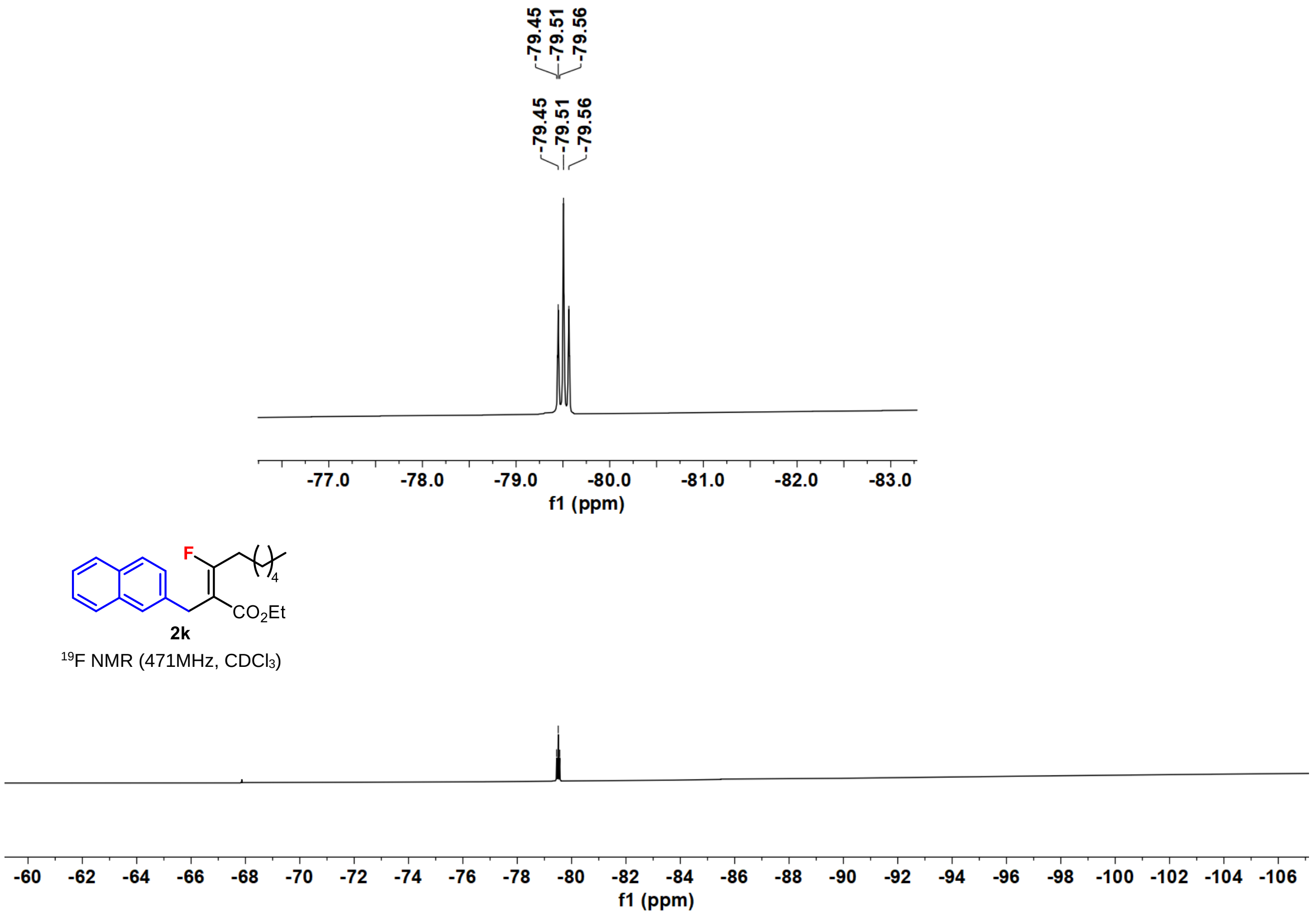
-79.45
-79.51
-79.56
f1 (ppm)
F
4
CO_2Et
2k
^{19}F NMR (471MHz, $CDCl_3$)
f1 (ppm)

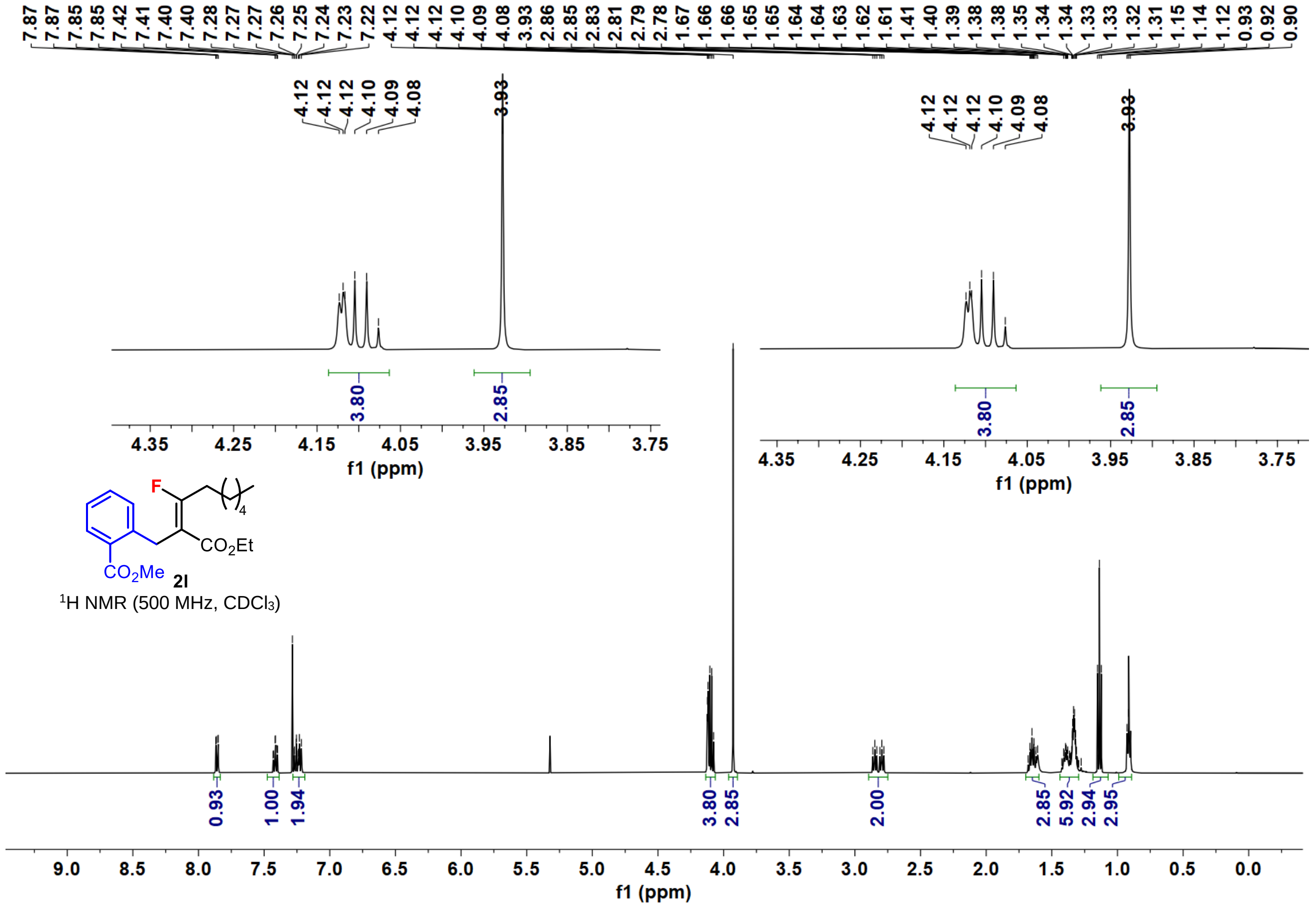
7.87 7.87 7.85 7.85 7.42 7.41 7.40 7.40 7.28 7.27 7.27 7.26 7.25 7.24 7.23 7.22 4.12 4.12 4.12 4.10 4.09 4.08 3.93 2.86 2.85 2.83 2.81 2.79 2.78 1.67 1.66 1.66 1.65 1.65 1.64 1.64 1.63 1.62 1.61 1.41 1.40 1.39 1.38 1.38 1.35 1.34 1.34 1.33 1.33 1.32 1.31 1.15 1.14 1.12 0.93 0.92 0.90
F
4
CO2Et
CO2Me
2l
1H NMR (500 MHz, CDCl3)
0.93 1.00 1.94 3.80 2.85 2.00 2.85 5.92 2.94 2.95
f1 (ppm)

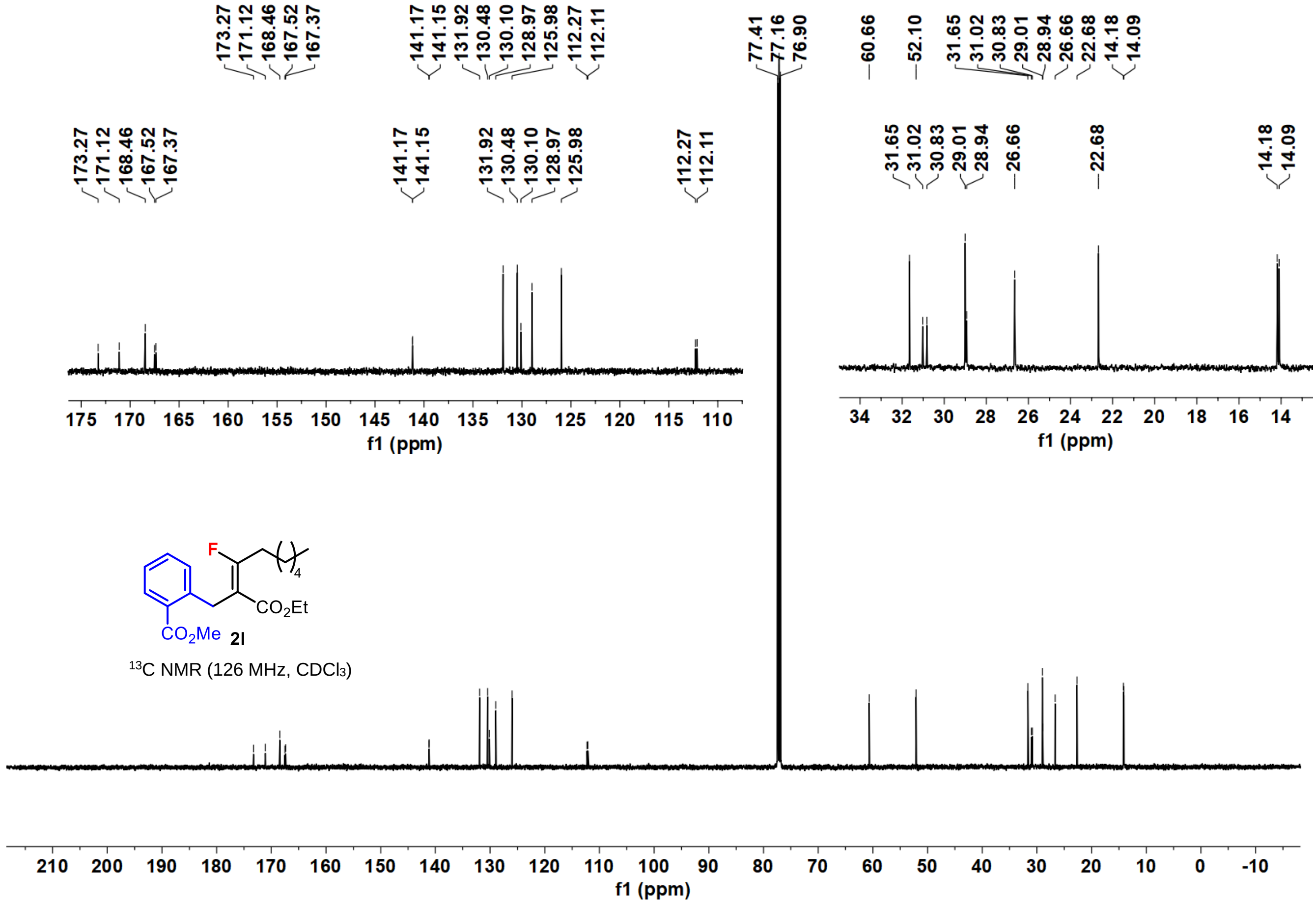
173.27 171.12 168.46 167.52 167.37 141.17 141.15 131.92 130.48 130.10 128.97 125.98 112.27 112.11 77.41 77.16 76.90 60.66 52.10 31.65 31.02 30.83 29.01 28.94 26.66 22.68 14.18 14.09
F
4
CO2Et
CO2Me
2l
13C NMR (126 MHz, CDCl3)
f1 (ppm)

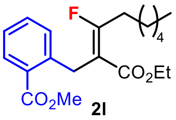

2l

^{19}F NMR (471MHz, $CDCl_3$)

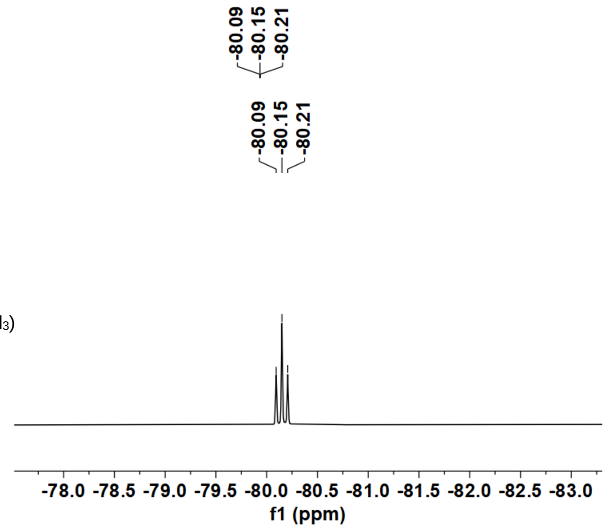

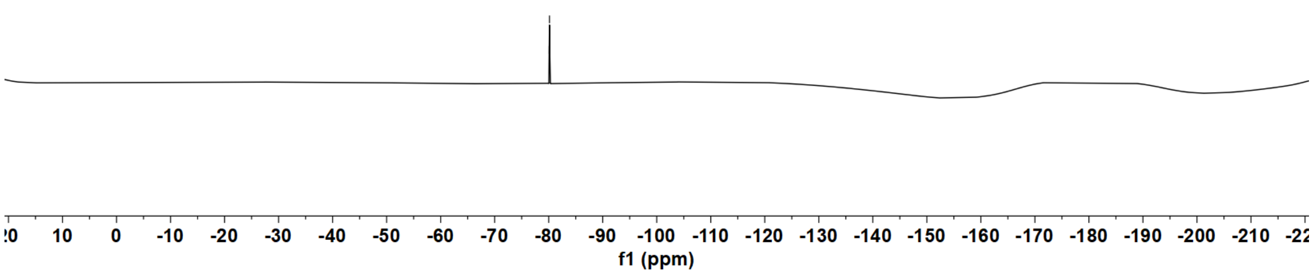

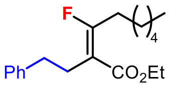

2m

^{1}H NMR (500 MHz, $CDCl_3$)

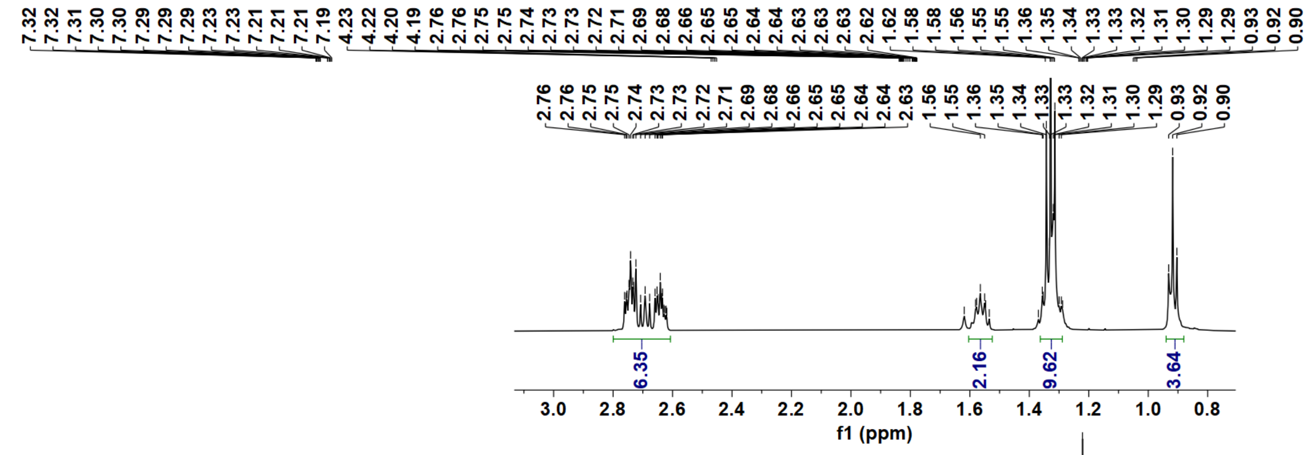

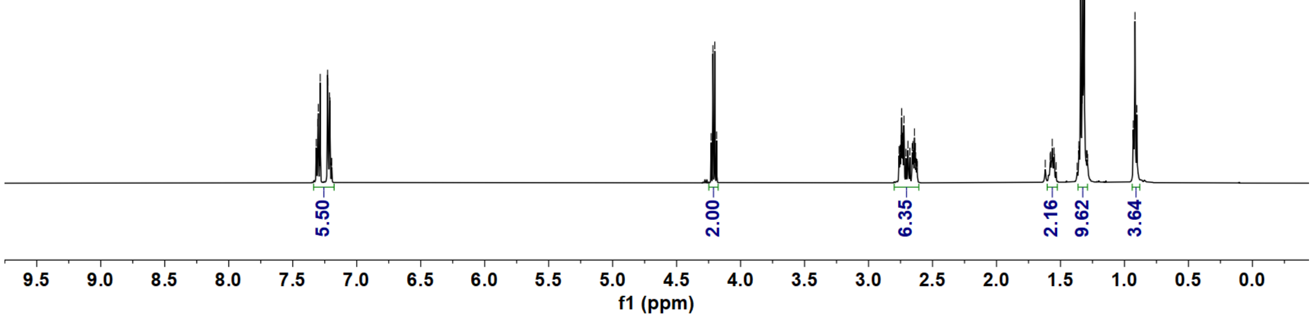

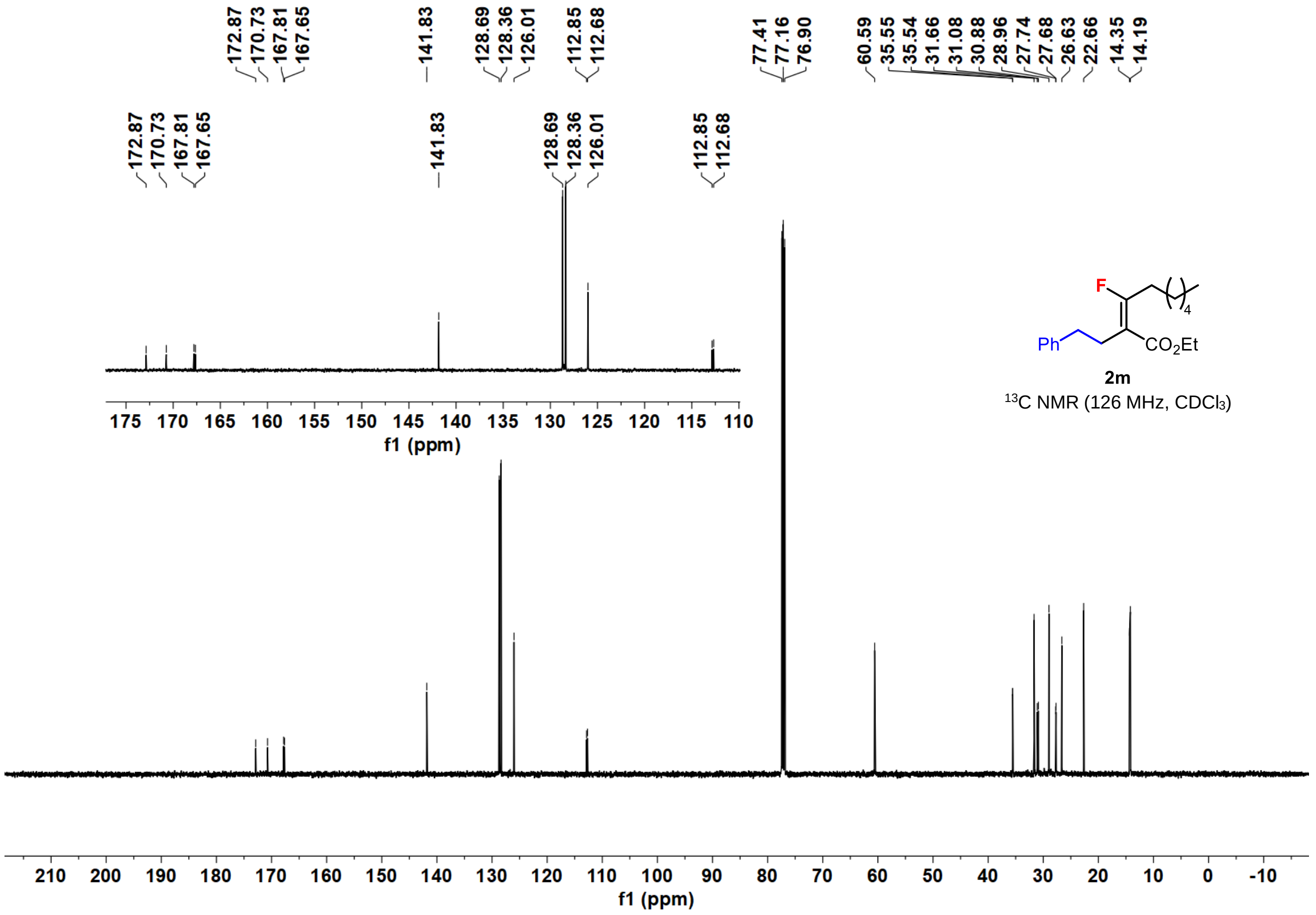
172.87
170.73
167.81
167.65
141.83
128.69
128.36
126.01
112.85
112.68
77.41
77.16
76.90
60.59
35.55
35.54
31.66
31.08
30.88
28.96
27.74
27.68
26.63
22.66
14.35
14.19
F
Ph
CO2Et
4
2m
13C NMR (126 MHz, CDCl3)
f1 (ppm)

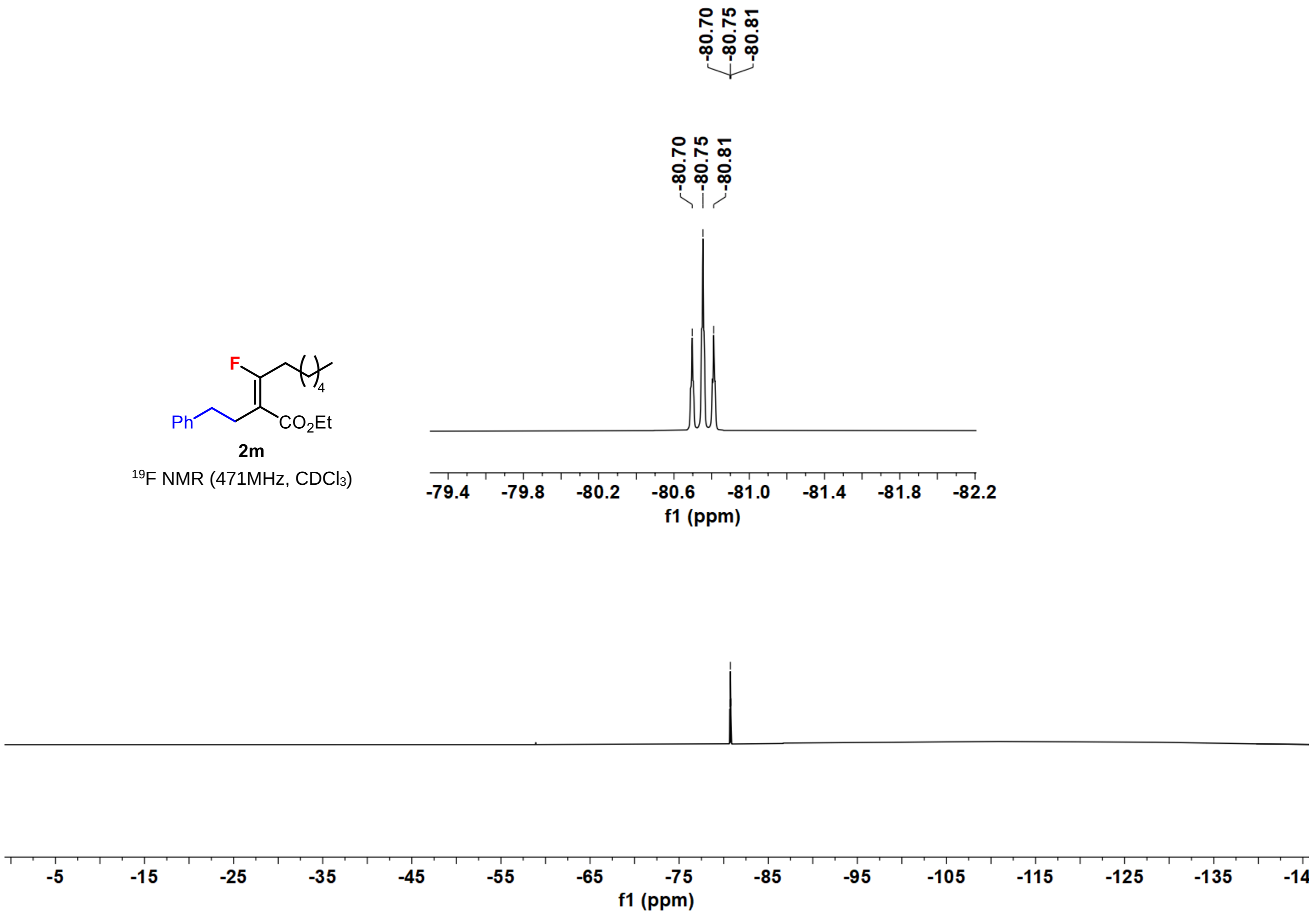
-80.70
-80.75
-80.81
F
Ph
CO2Et
4
2m
19F NMR (471MHz, CDCl3)
f1 (ppm)

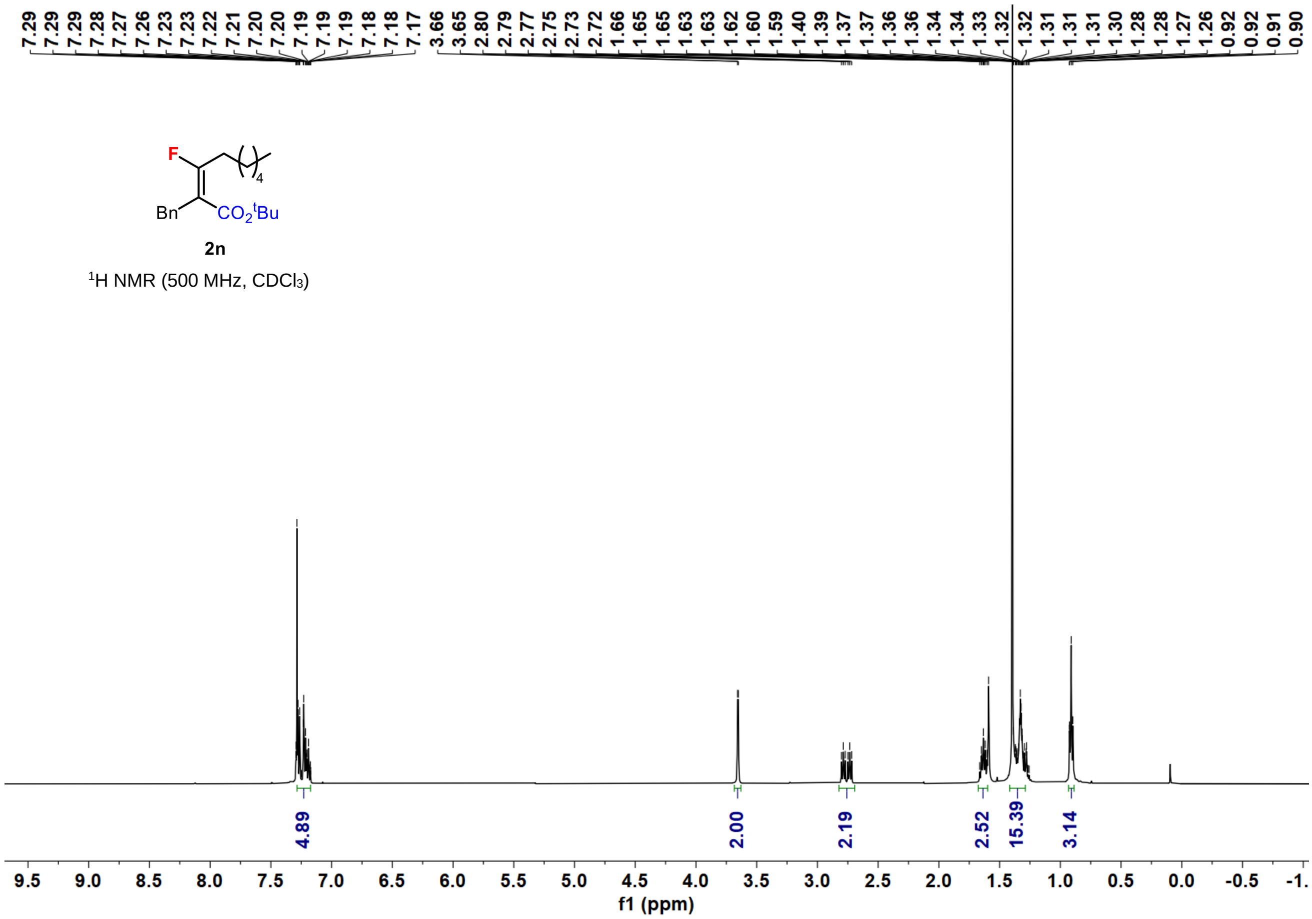

2n

^{1}H NMR (500 MHz, CDCl$_3$)

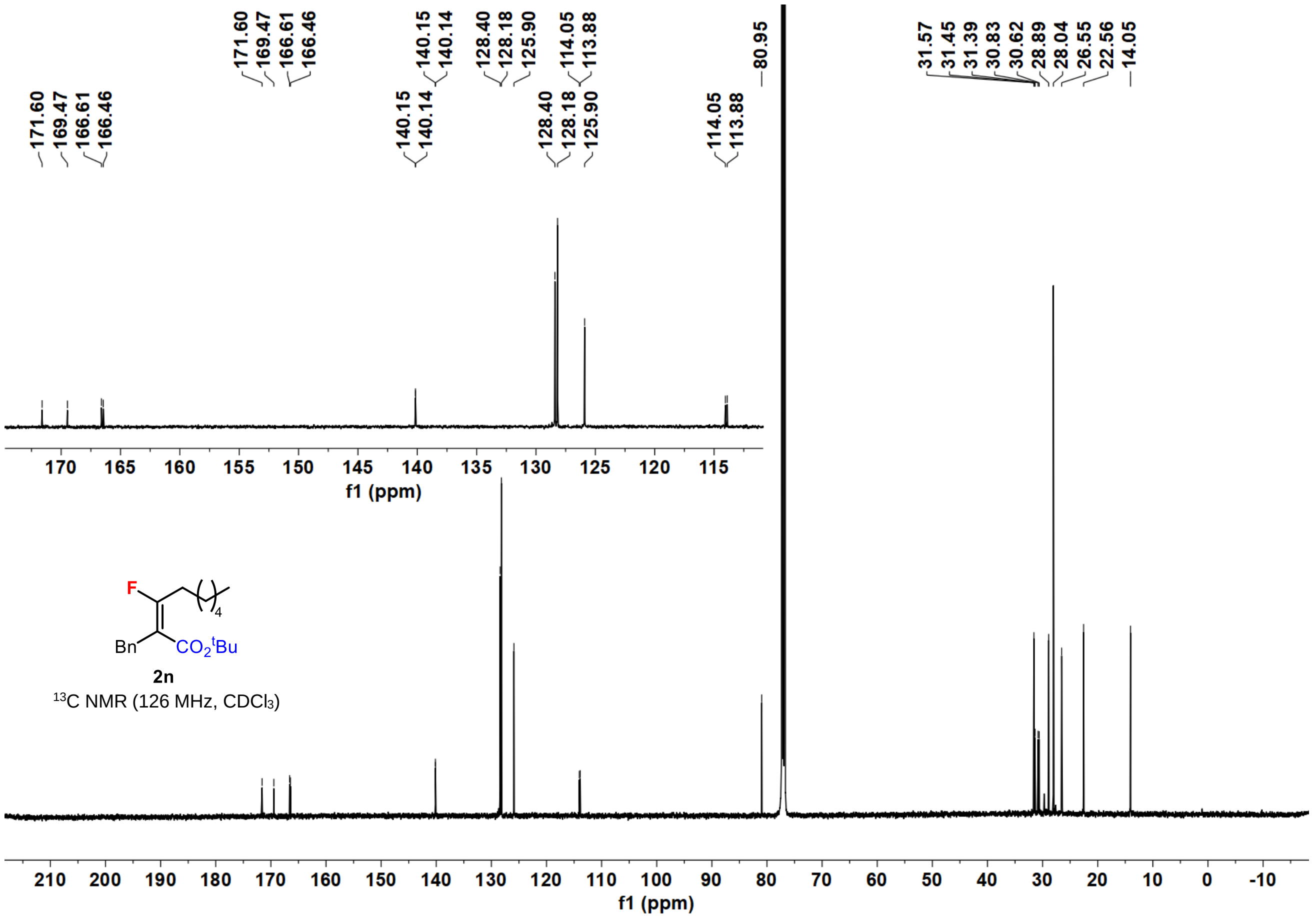

2n

^{13}C NMR (126 MHz, CDCl$_3$)

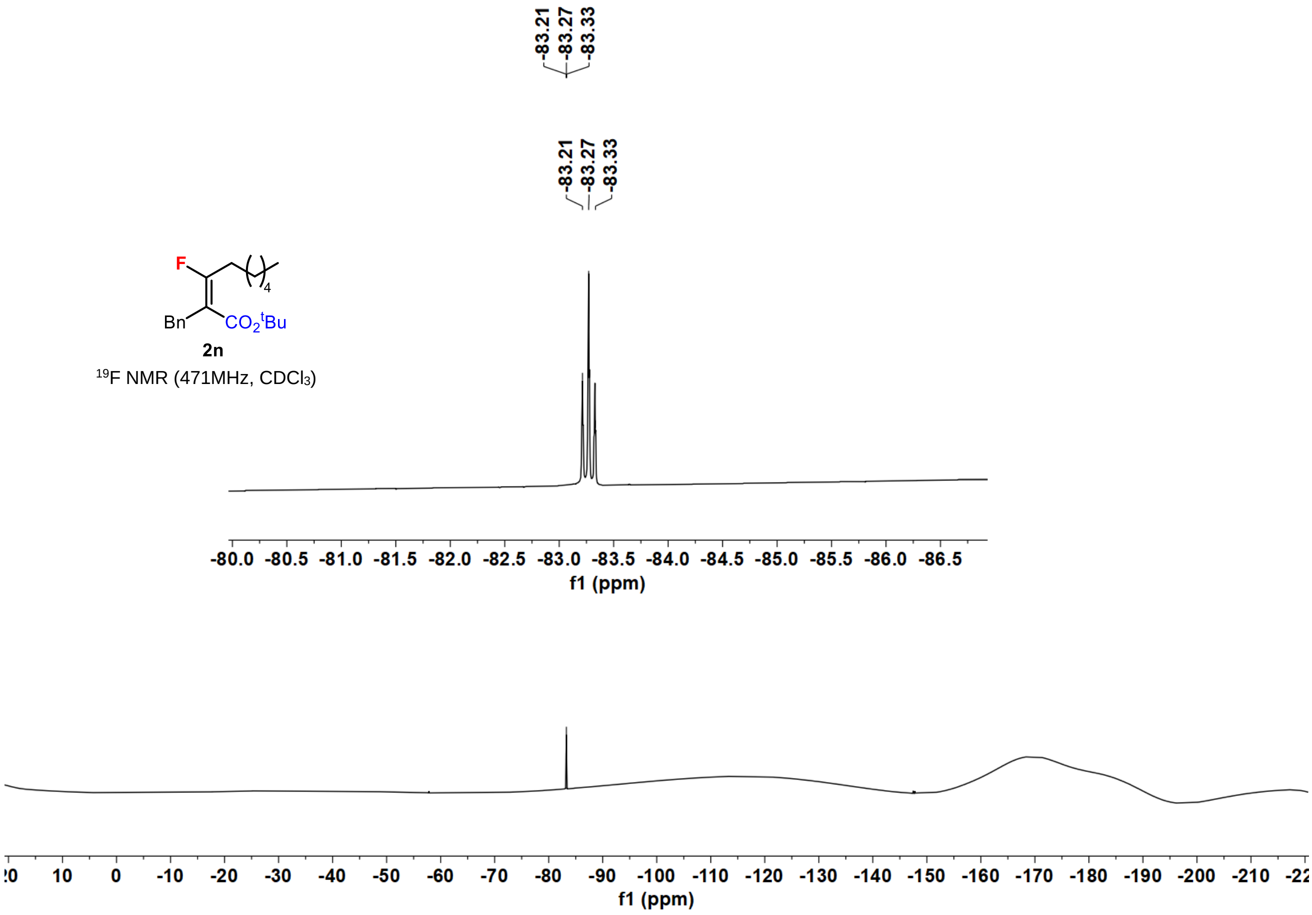

2n

^{19}F NMR (471MHz, $CDCl_3$)

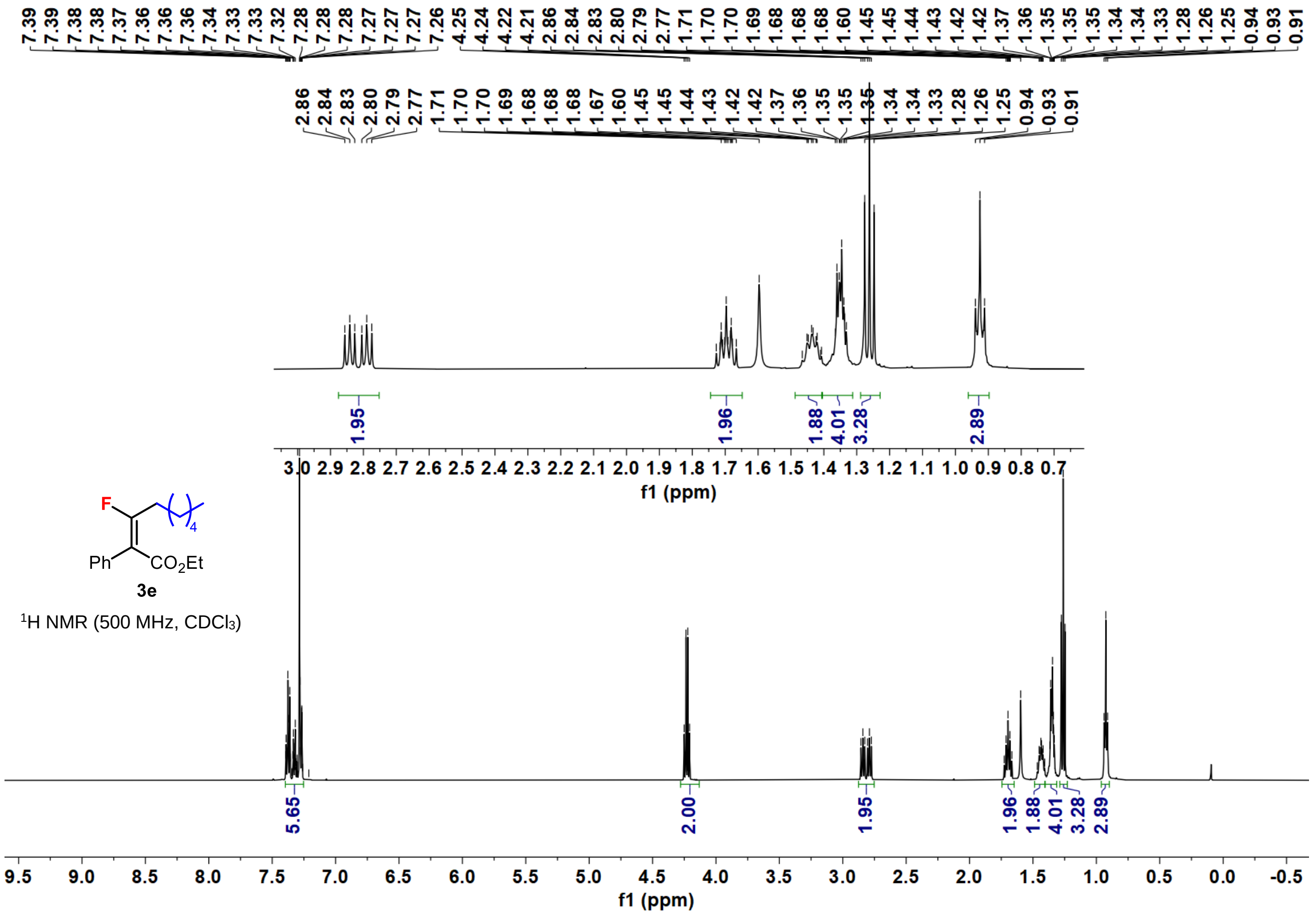

3e

^1H NMR (500 MHz, $CDCl_3$)

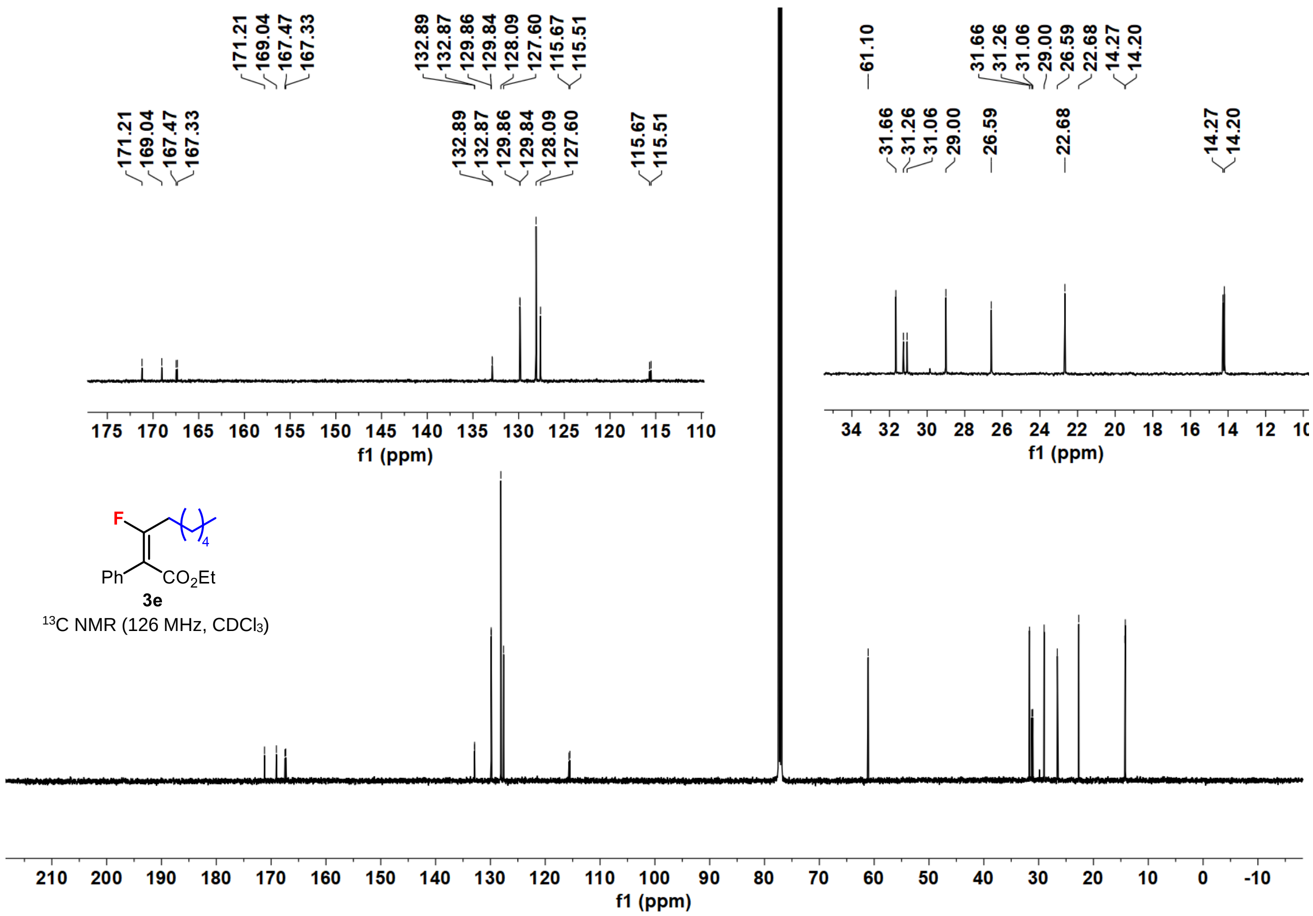
171.21
169.04
167.47
167.33
132.89
132.87
129.86
129.84
128.09
127.60
115.67
115.51
61.10
31.66
31.26
31.06
29.00
26.59
22.68
14.27
14.20
f1 (ppm)
F
4
Ph
CO_2Et
3e
^{13}C NMR (126 MHz, CDCl3)

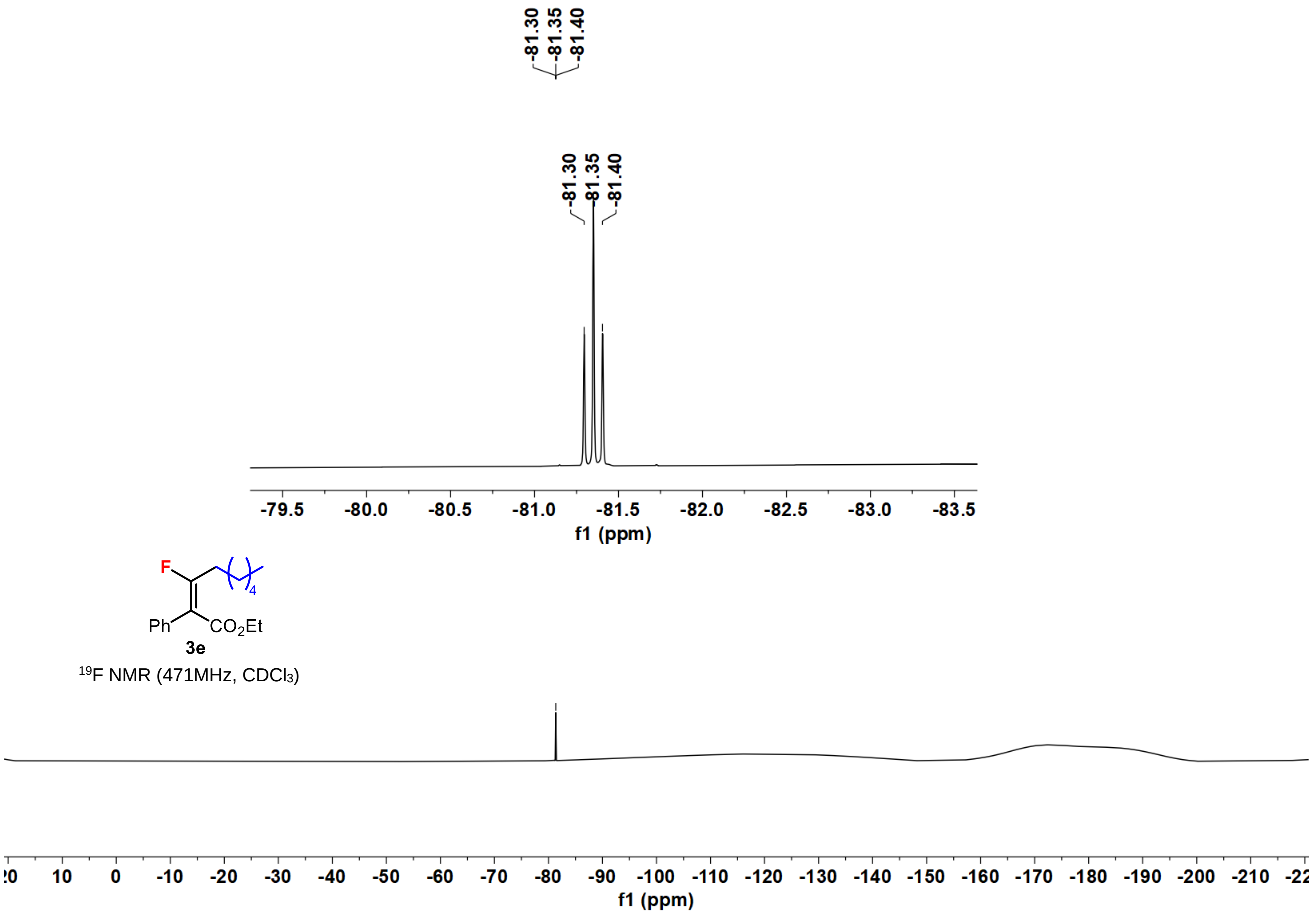
-81.30
-81.35
-81.40
f1 (ppm)
F
4
Ph
CO_2Et
3e
^{19}F NMR (471MHz, CDCl3)

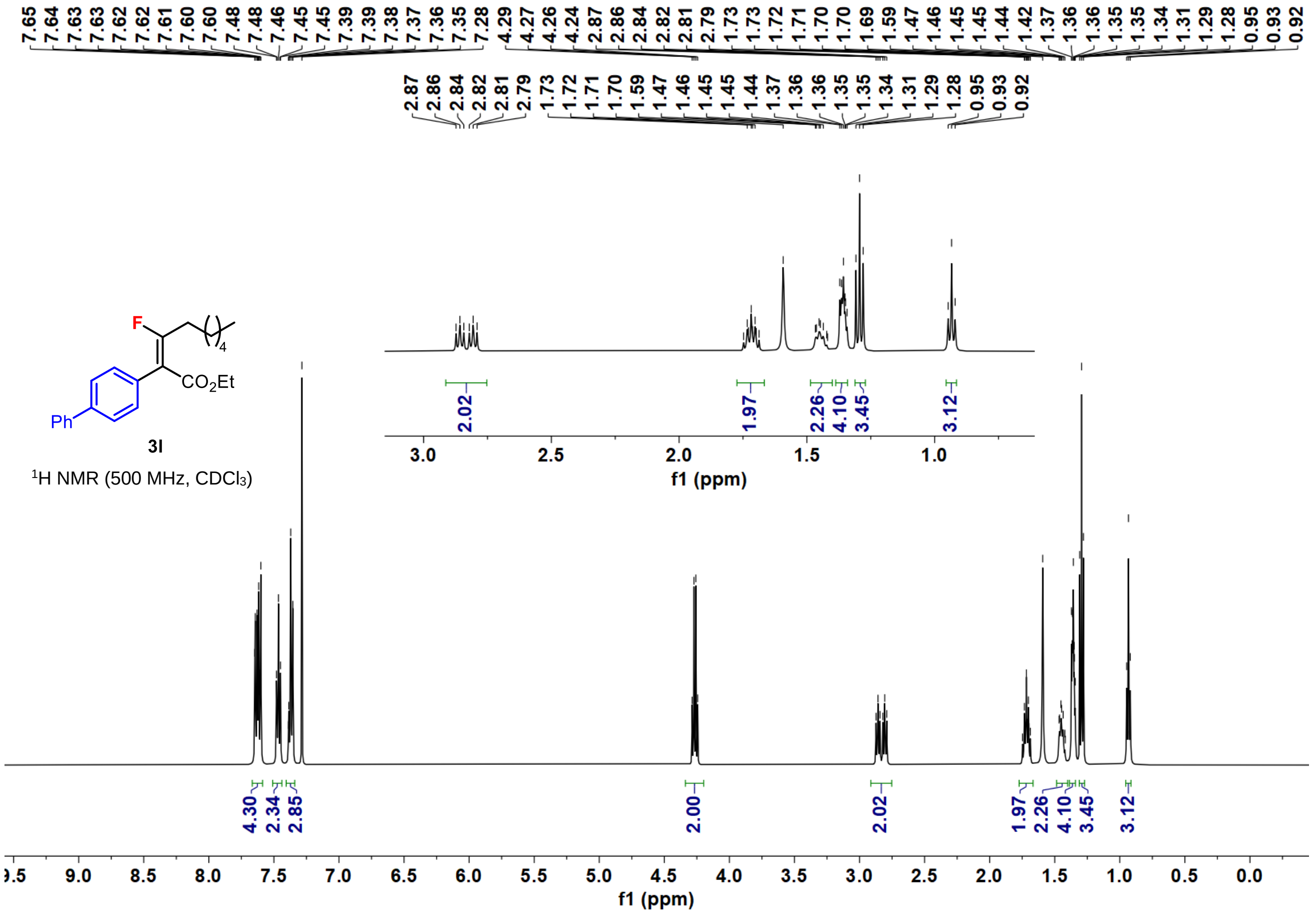
3l
1H NMR (500 MHz, CDCl3)

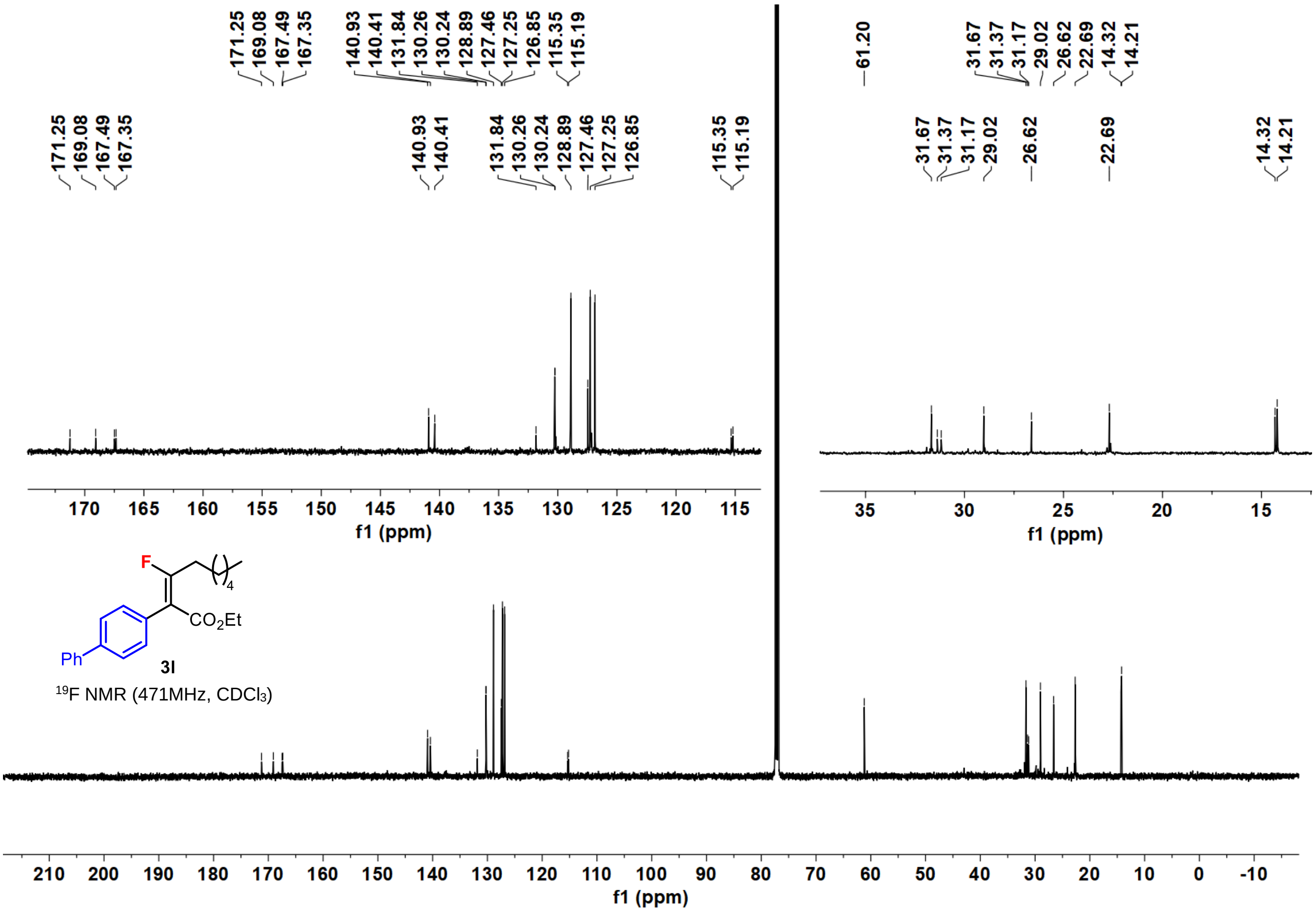
3l
19F NMR (471MHz, CDCl3)

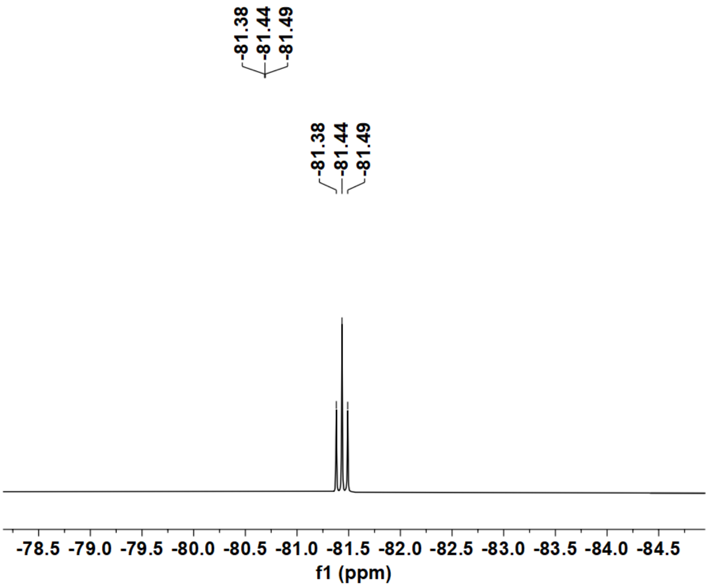

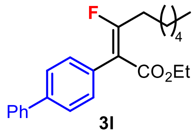

3l

^{13}C NMR (126 MHz, CDCl$_3$)

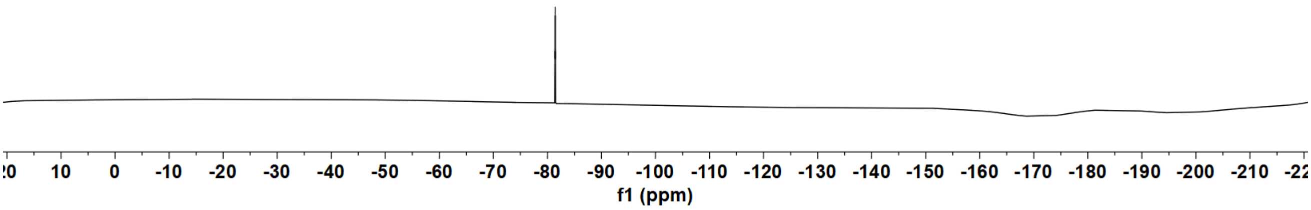

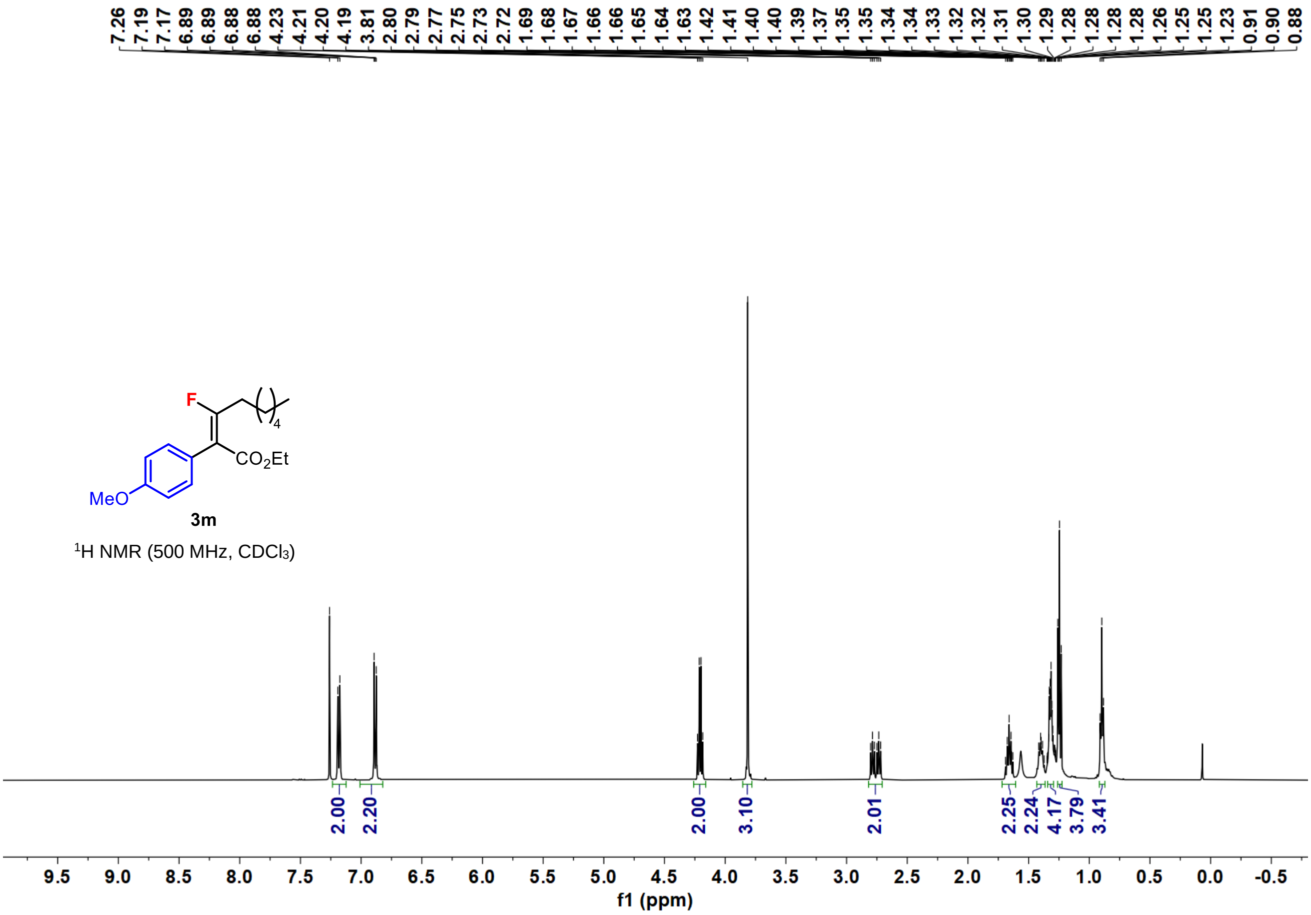

3m

^{1}H NMR (500 MHz, CDCl$_3$)

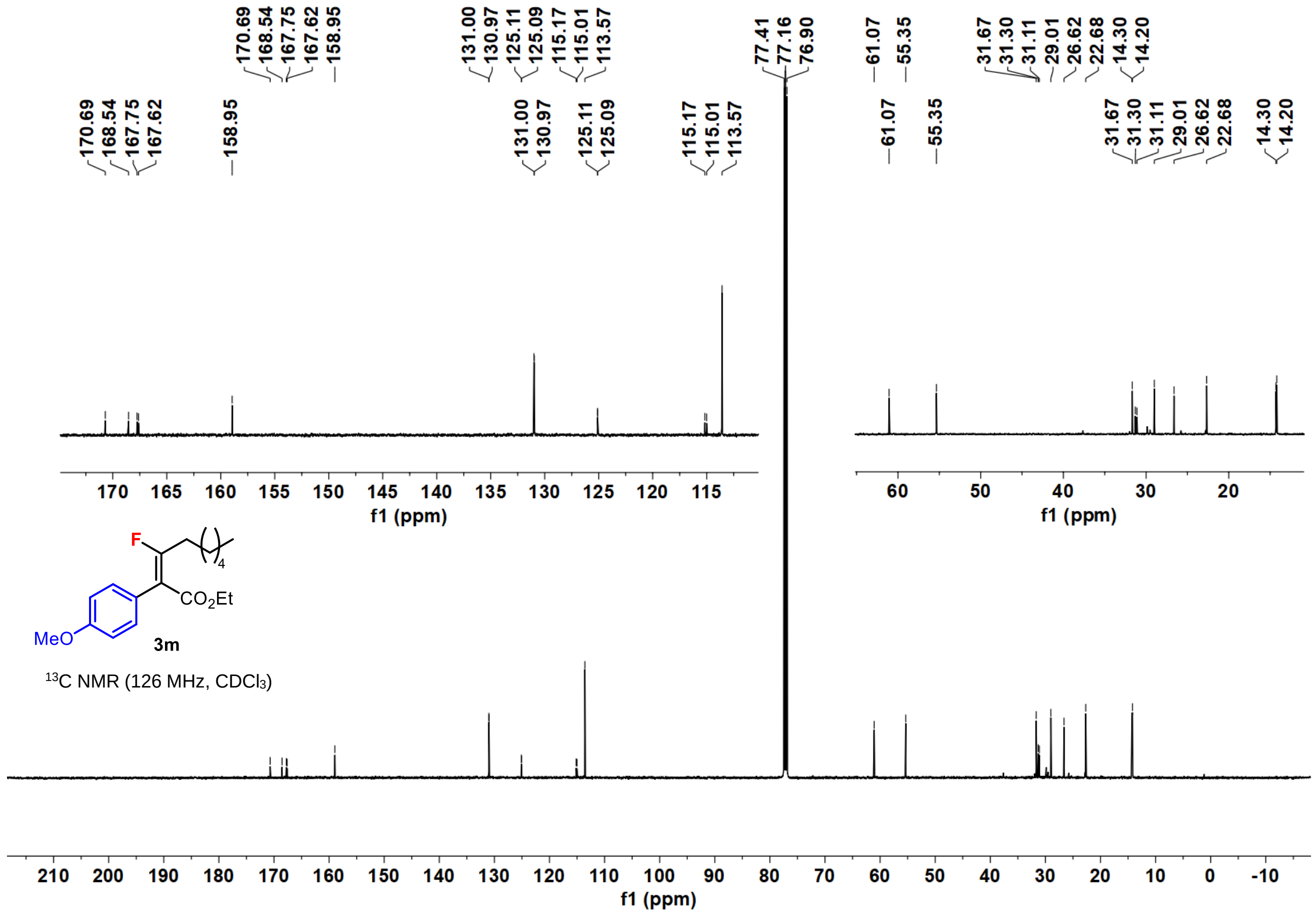
F
CO_2Et
MeO
3m
^{13}C NMR (126 MHz, $CDCl_3$)
f1 (ppm)

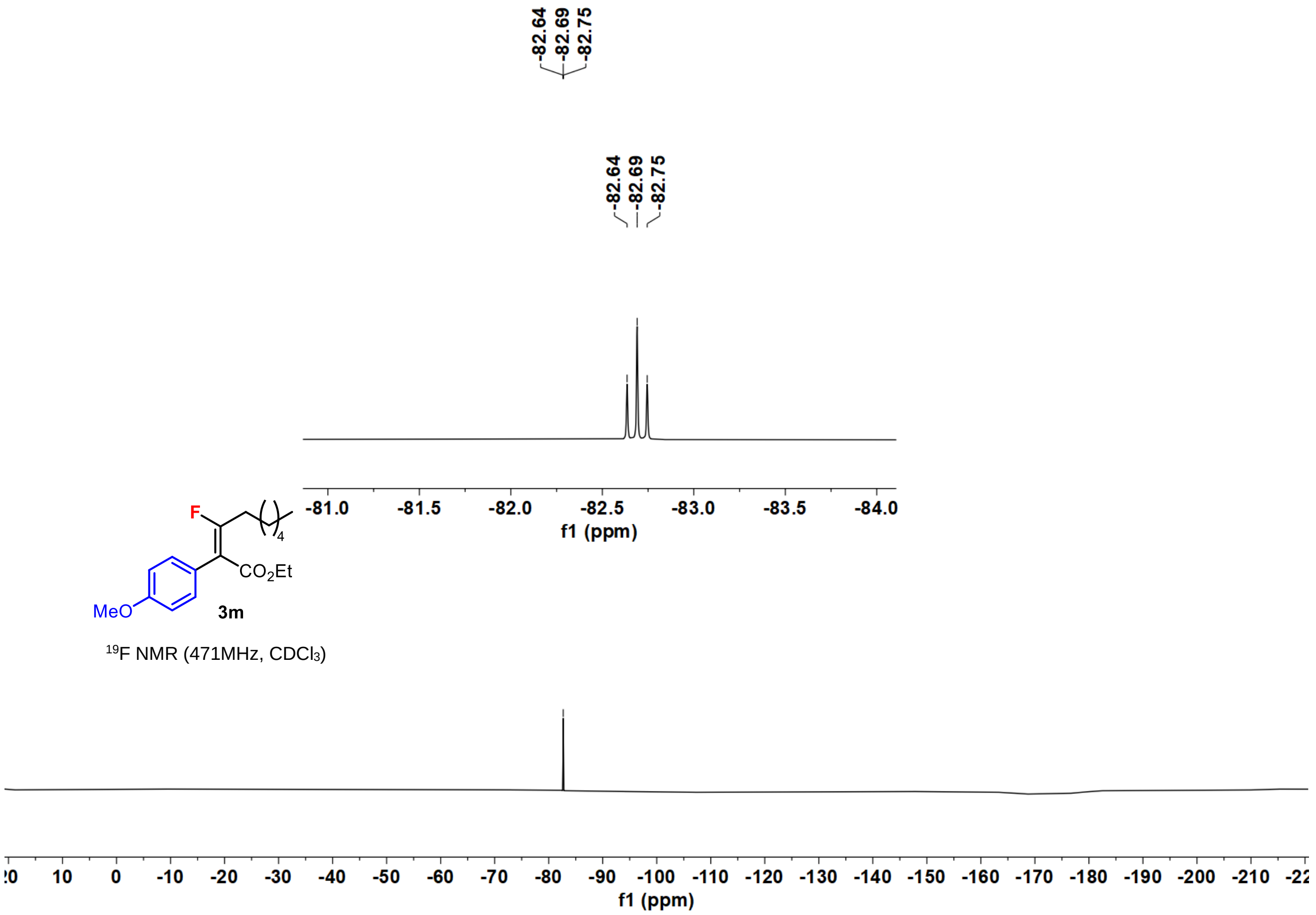
F
CO_2Et
MeO
3m
^{19}F NMR (471MHz, $CDCl_3$)
f1 (ppm)

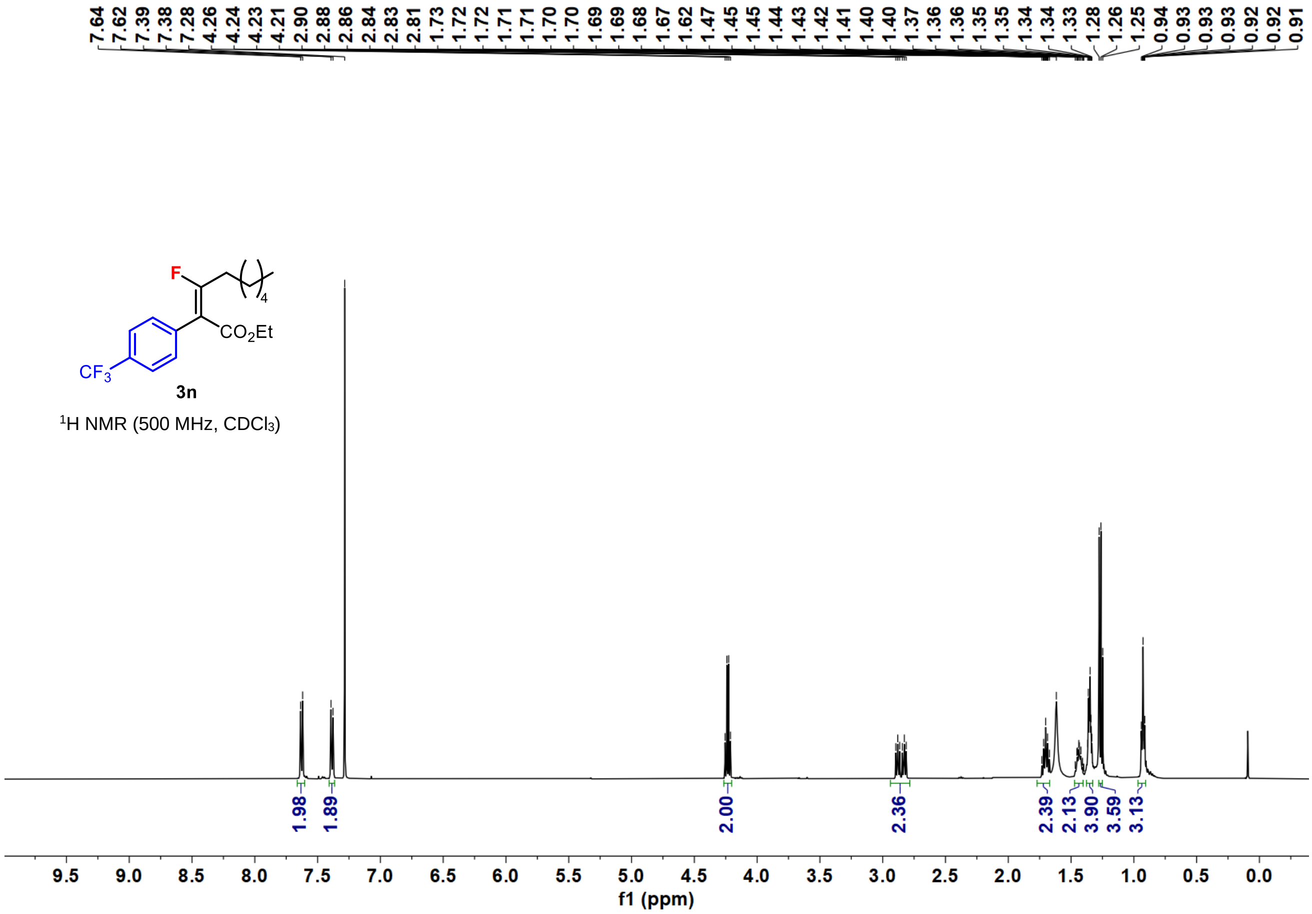
7.64 7.62 7.39 7.38 7.28 4.26 4.24 4.23 4.21 2.90 2.88 2.86 2.84 2.83 2.81 1.73 1.72 1.72 1.71 1.71 1.70 1.70 1.69 1.69 1.68 1.67 1.62 1.47 1.45 1.45 1.44 1.43 1.42 1.41 1.40 1.40 1.37 1.36 1.36 1.35 1.35 1.34 1.34 1.33 1.28 1.26 1.25 0.94 0.93 0.93 0.93 0.92 0.92 0.91
F
CO2Et
CF3
4
3n
1H NMR (500 MHz, CDCl3)
1.98 1.89 2.00 2.36 2.39 2.13 3.90 3.59 3.13
9.5 9.0 8.5 8.0 7.5 7.0 6.5 6.0 5.5 5.0 4.5 4.0 3.5 3.0 2.5 2.0 1.5 1.0 0.5 0.0
f1 (ppm)

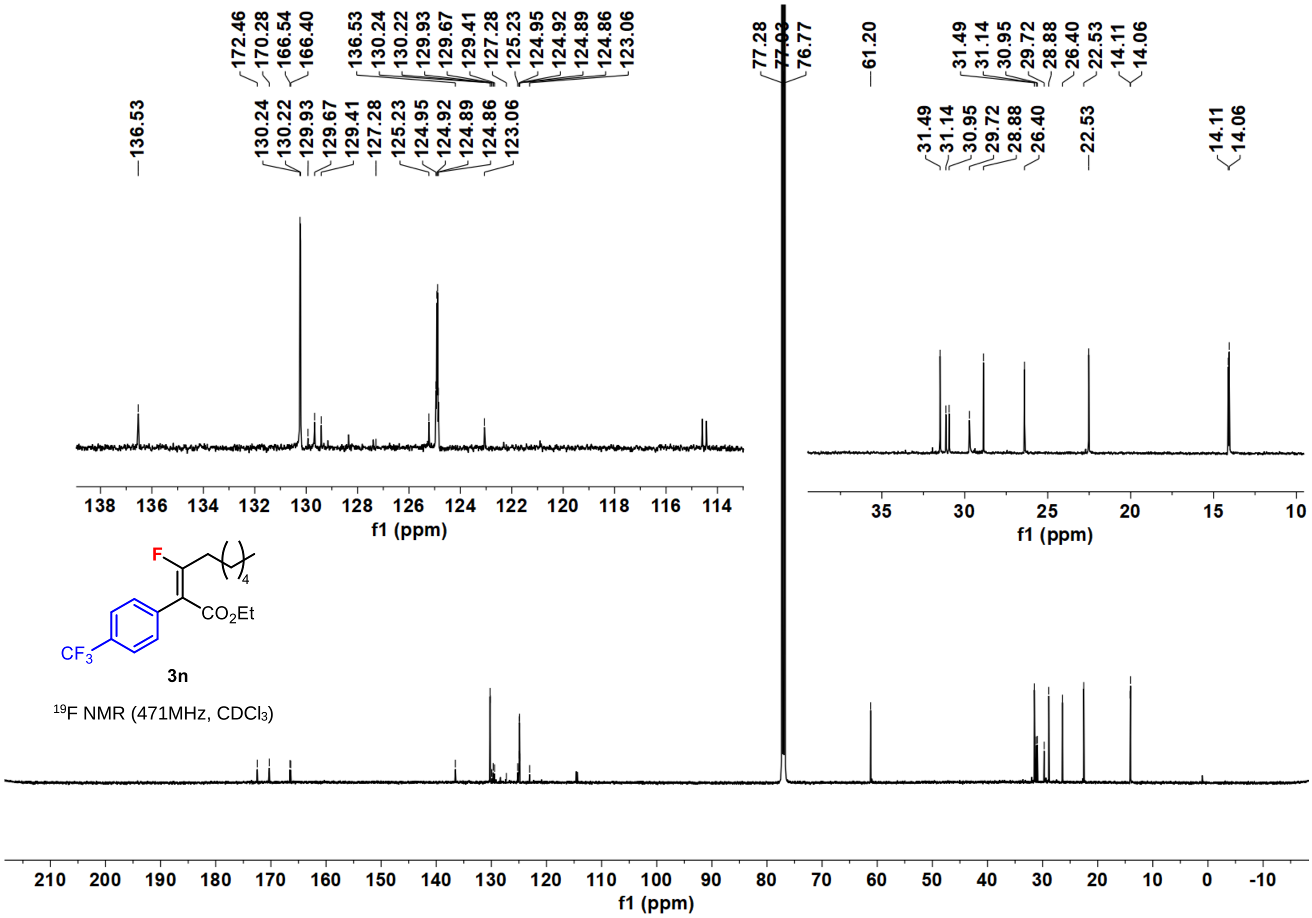
172.46 170.28 166.54 166.40 136.53 130.24 130.22 129.93 129.67 129.41 127.28 125.23 124.95 124.92 124.89 124.86 123.06
77.28 77.03 76.77
61.20
31.49 31.14 30.95 29.72 28.88 26.40 22.53 14.11 14.06
136.53
130.24 130.22 129.93 129.67 129.41 127.28 125.23 124.95 124.92 124.89 124.86 123.06
31.49 31.14 30.95 29.72 28.88 26.40
22.53
14.11 14.06
138 136 134 132 130 128 126 124 122 120 118 116 114
f1 (ppm)
35 30 25 20 15 10
f1 (ppm)
F
CO2Et
CF3
4
3n
19F NMR (471MHz, CDCl3)
210 200 190 180 170 160 150 140 130 120 110 100 90 80 70 60 50 40 30 20 10 0 -10
f1 (ppm)

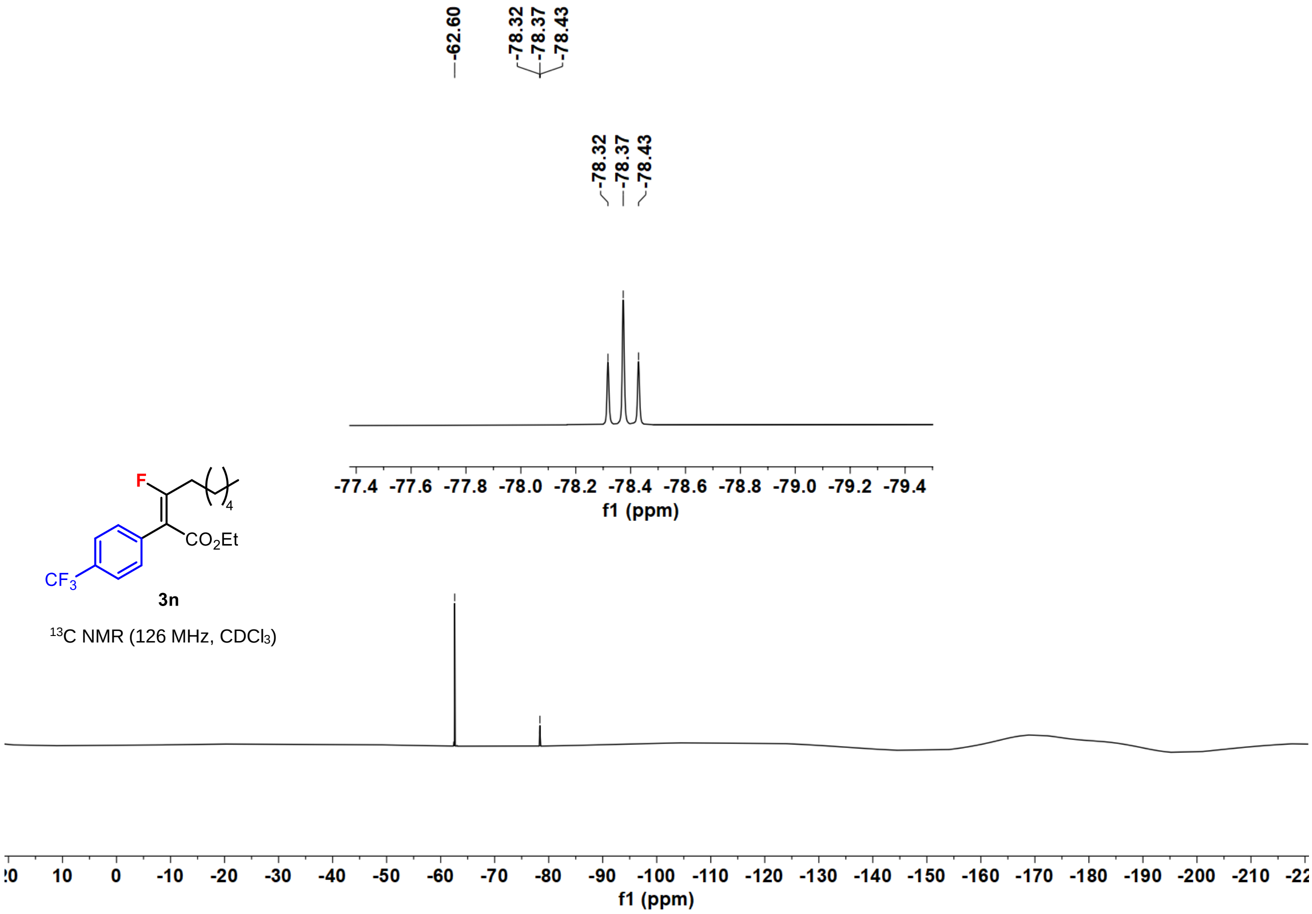
-62.60
-78.32
-78.37
-78.43
F
CO2Et
CF3
3n
13C NMR (126 MHz, CDCl3)
-77.4 -77.6 -77.8 -78.0 -78.2 -78.4 -78.6 -78.8 -79.0 -79.2 -79.4
f1 (ppm)
10 0 -10 -20 -30 -40 -50 -60 -70 -80 -90 -100 -110 -120 -130 -140 -150 -160 -170 -180 -190 -200 -210
f1 (ppm)

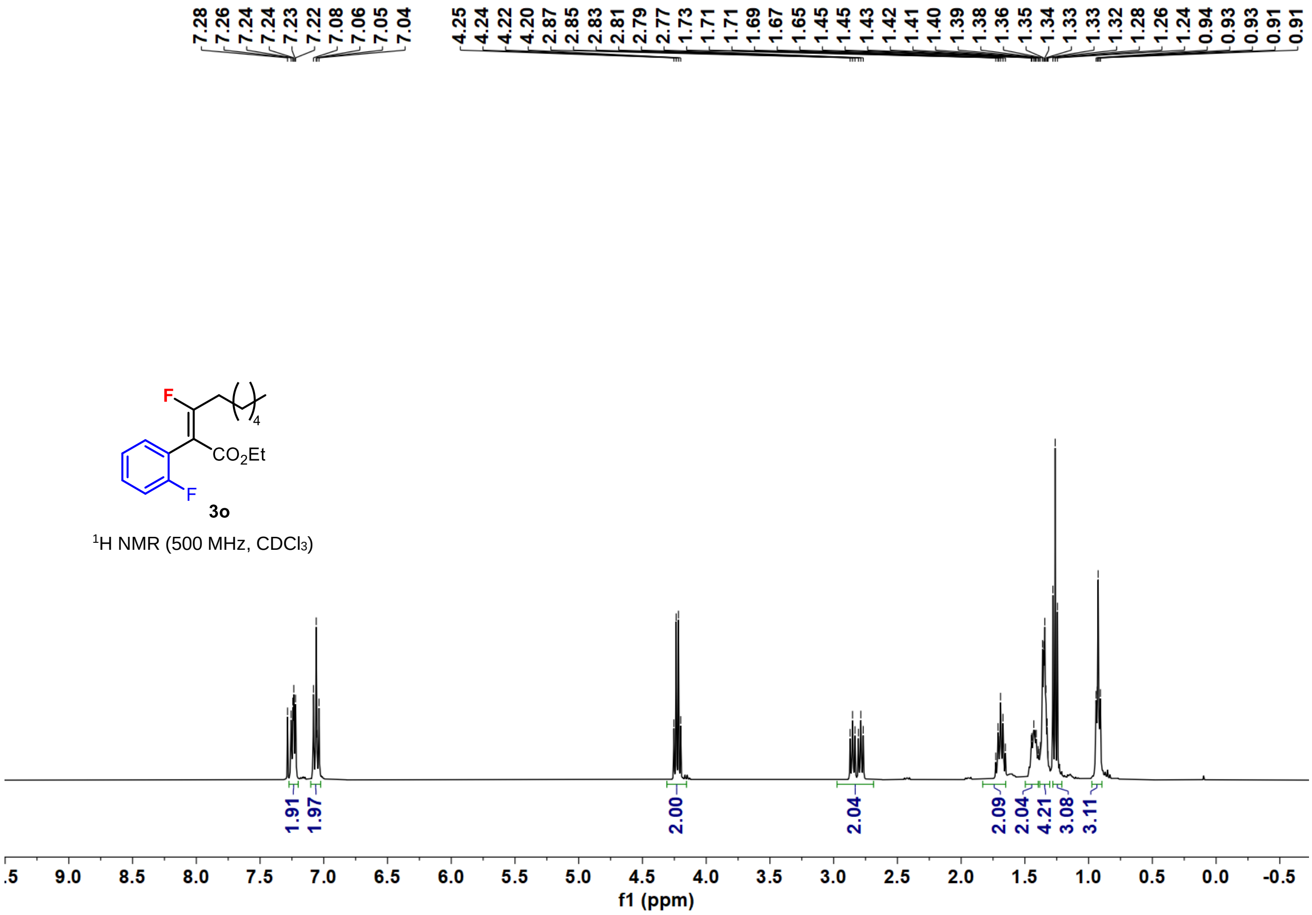
7.28 7.26 7.24 7.24 7.23 7.22 7.08 7.06 7.05 7.04
4.25 4.24 4.22 4.20 2.87 2.85 2.83 2.81 2.79 2.77 1.73 1.71 1.71 1.69 1.67 1.65 1.45 1.45 1.43 1.42 1.41 1.40 1.39 1.38 1.36 1.35 1.34 1.33 1.33 1.32 1.28 1.26 1.24 0.94 0.93 0.93 0.91 0.91
F
CO2Et
F
3o
1H NMR (500 MHz, CDCl3)
1.91 1.97 2.00 2.04 2.09 2.04 4.21 3.08 3.11
9.0 8.5 8.0 7.5 7.0 6.5 6.0 5.5 5.0 4.5 4.0 3.5 3.0 2.5 2.0 1.5 1.0 0.5 0.0 -0.5
f1 (ppm)

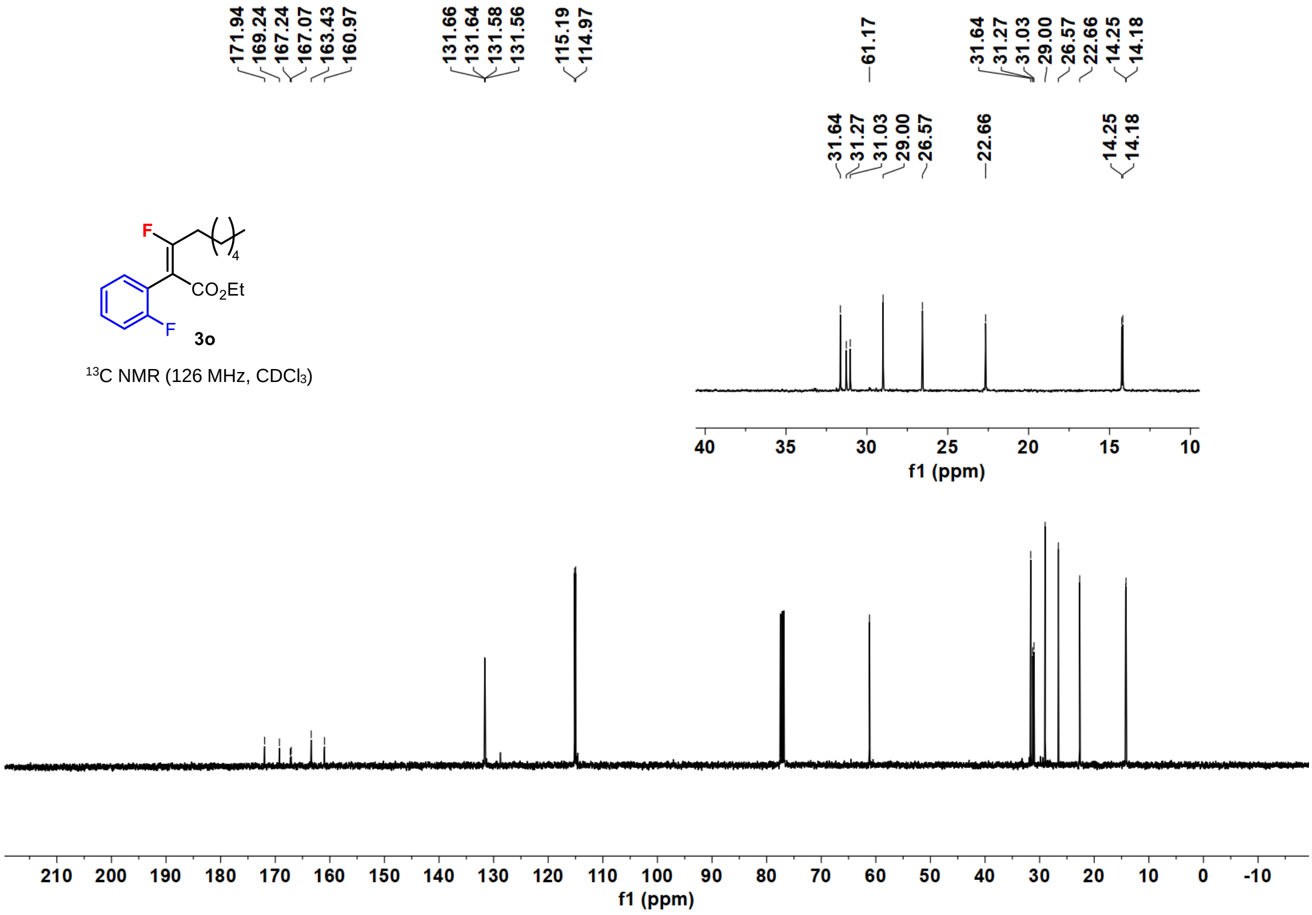
171.94
169.24
167.24
167.07
163.43
160.97
131.66
131.64
131.58
131.56
115.19
114.97
61.17
31.64
31.27
31.03
29.00
26.57
22.66
14.25
14.18
F
CO_2Et
4
F
3o
^{13}C NMR (126 MHz, $CDCl_3$)
f1 (ppm)
f1 (ppm)

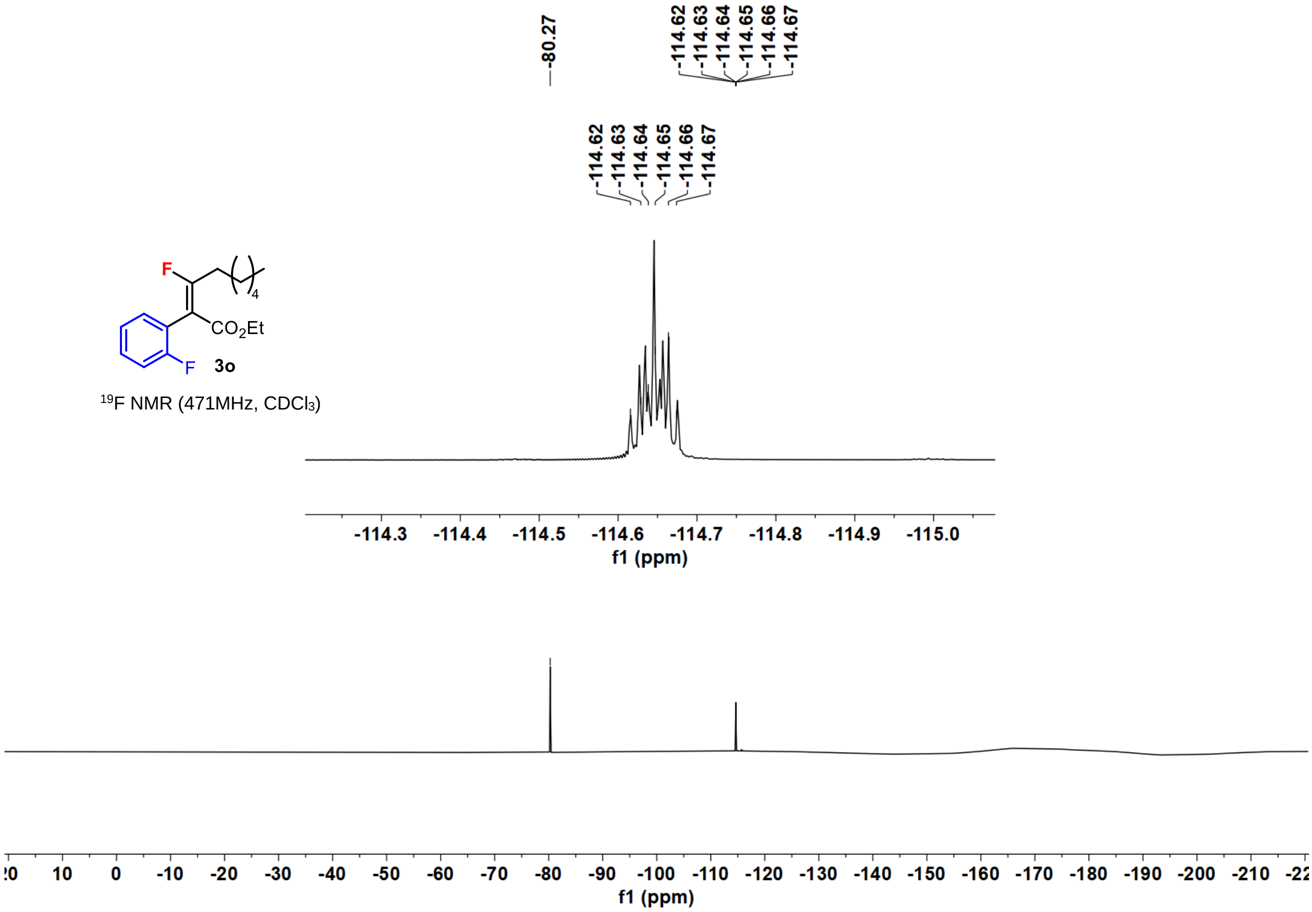
-80.27
-114.62
-114.63
-114.64
-114.65
-114.66
-114.67
F
CO_2Et
4
F
3o
^{19}F NMR (471MHz, $CDCl_3$)
f1 (ppm)
f1 (ppm)

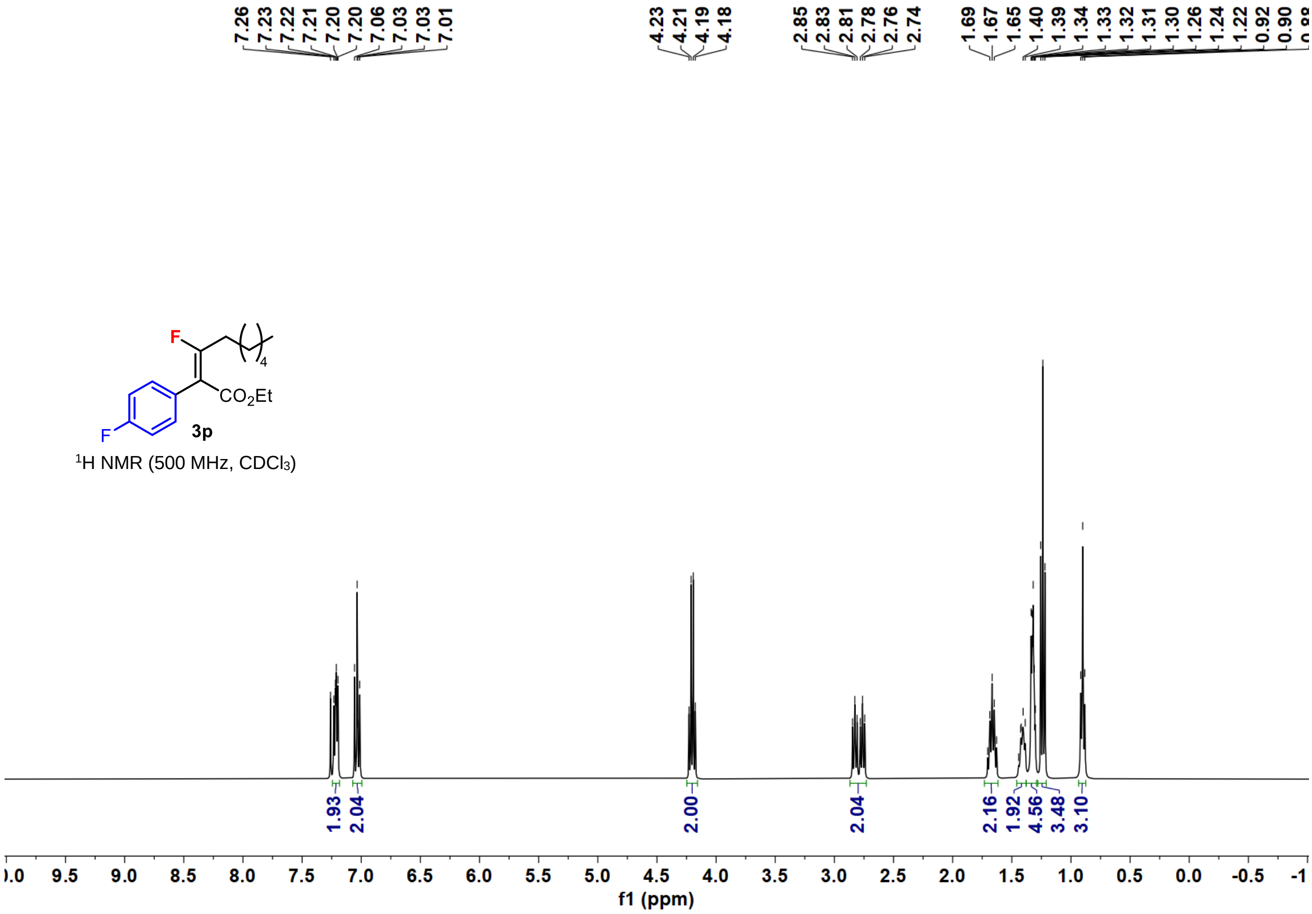

3p

^1H NMR (500 MHz, $CDCl_3$)

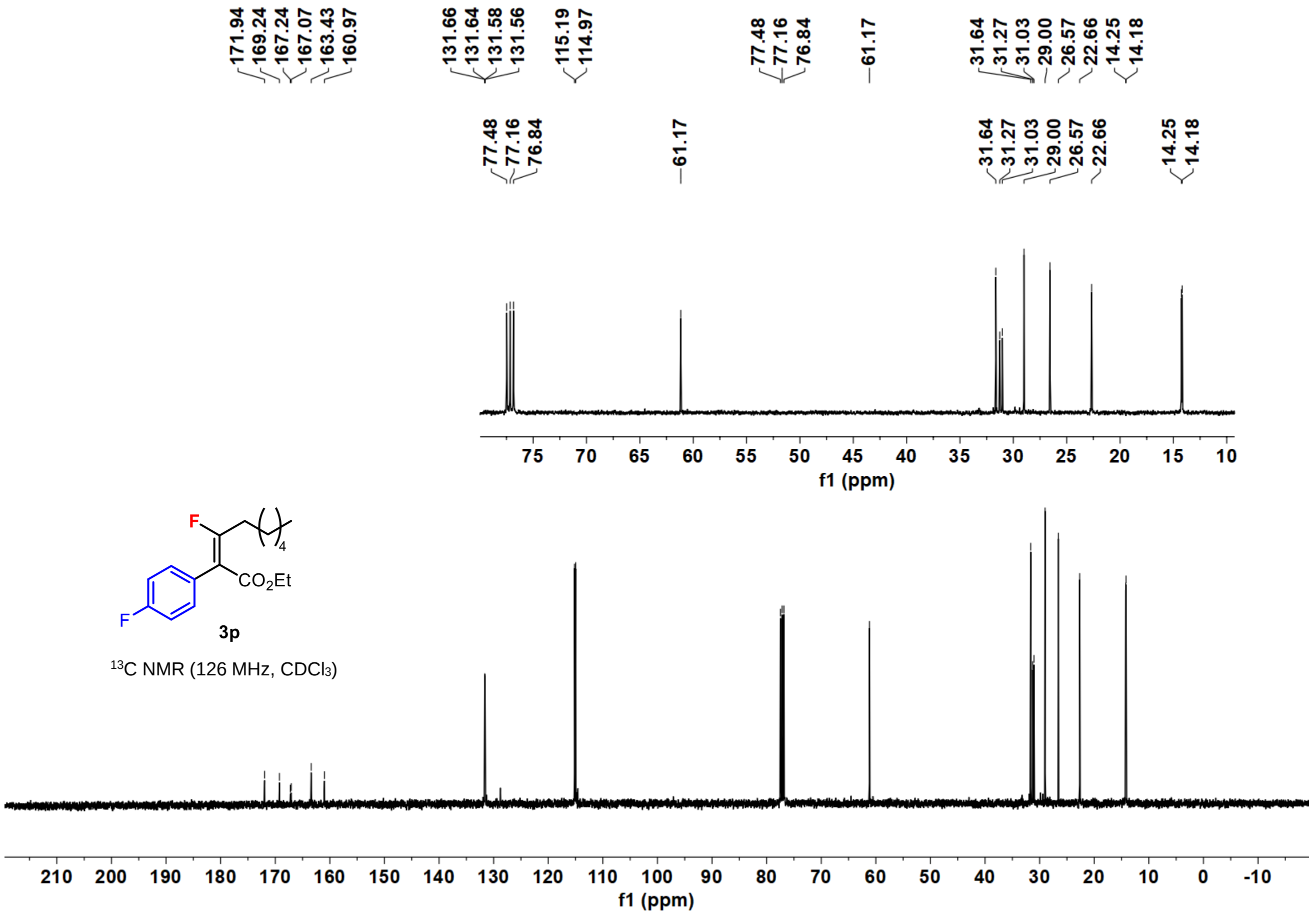

3p

^{13}C NMR (126 MHz, $CDCl_3$)

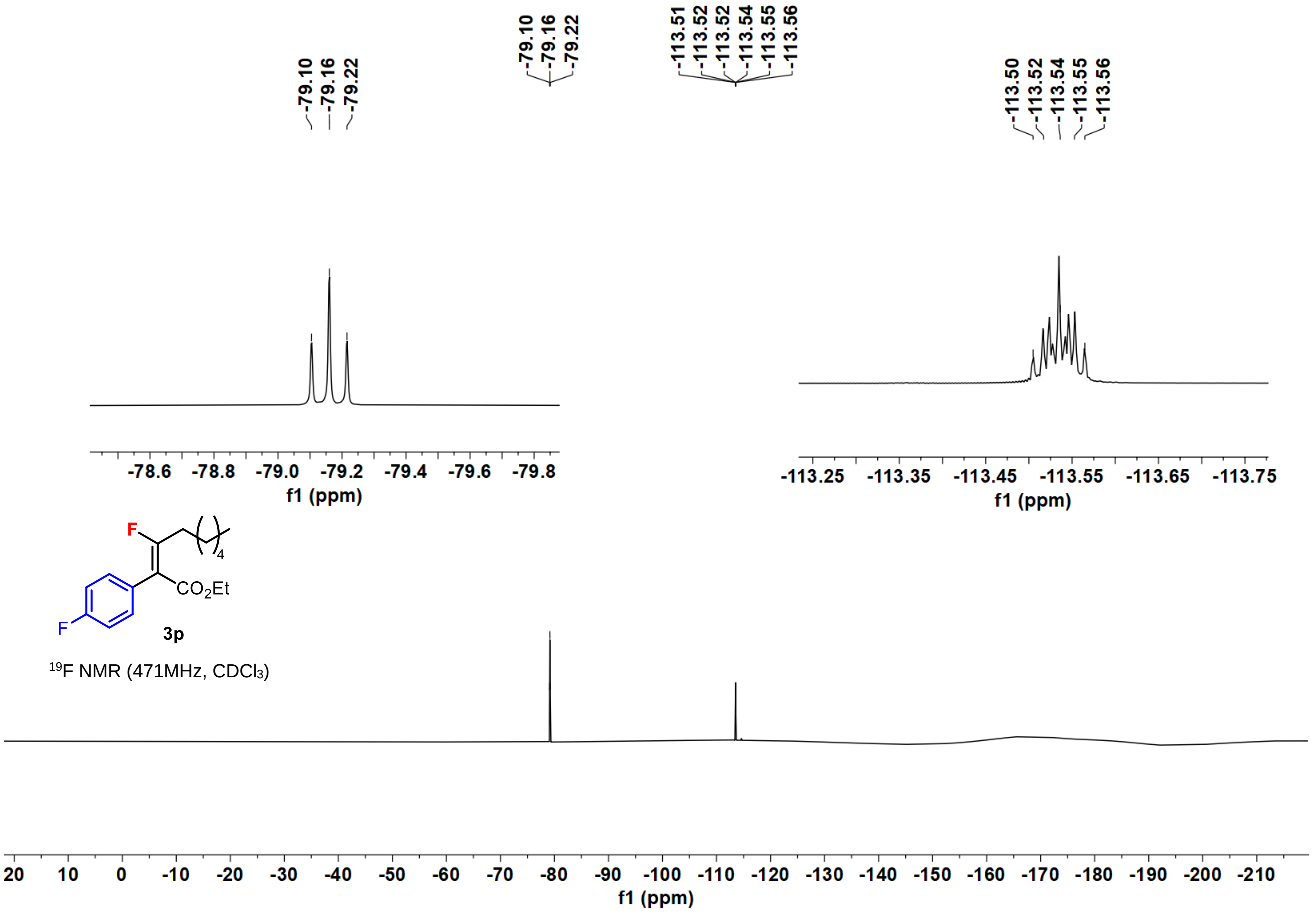
-79.10
-79.16
-79.22
-79.10
-79.16
-79.22
-113.51
-113.52
-113.52
-113.54
-113.55
-113.56
-113.50
-113.52
-113.54
-113.55
-113.56
-78.6 -78.8 -79.0 -79.2 -79.4 -79.6 -79.8
f1 (ppm)
-113.25 -113.35 -113.45 -113.55 -113.65 -113.75
f1 (ppm)
F
4
CO2Et
F
3p
19F NMR (471MHz, CDCl3)
20 10 0 -10 -20 -30 -40 -50 -60 -70 -80 -90 -100 -110 -120 -130 -140 -150 -160 -170 -180 -190 -200 -210
f1 (ppm)

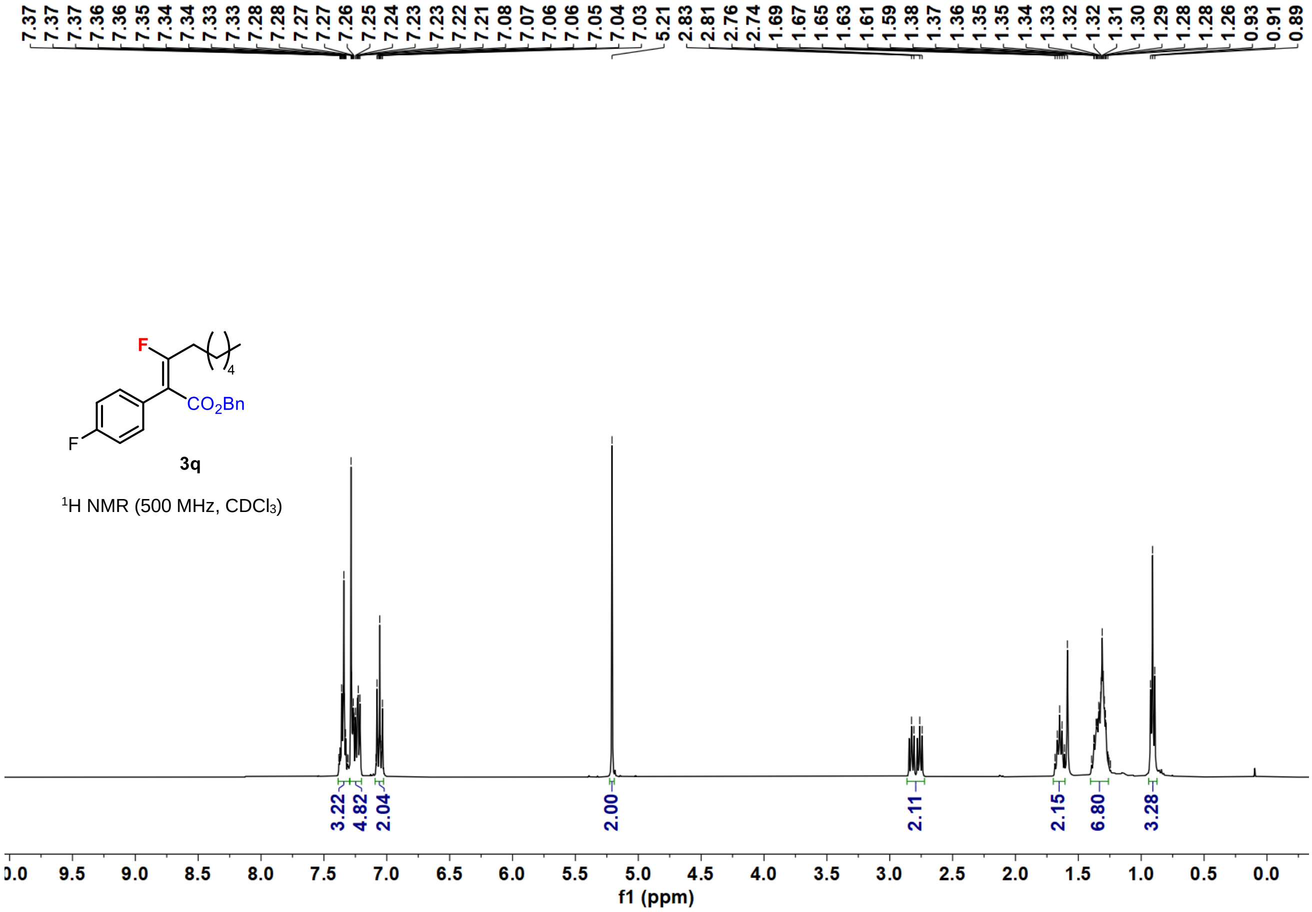
7.37 7.37 7.37 7.36 7.36 7.35 7.34 7.34 7.33 7.33 7.28 7.28 7.27 7.27 7.26 7.25 7.24 7.23 7.23 7.22 7.21 7.08 7.07 7.06 7.06 7.05 7.04 7.03 5.21 2.83 2.81 2.76 2.74 1.69 1.67 1.65 1.63 1.61 1.59 1.38 1.37 1.36 1.35 1.34 1.33 1.32 1.32 1.31 1.30 1.29 1.28 1.28 1.26 0.93 0.91 0.89
F
4
CO2Bn
F
3q
1H NMR (500 MHz, CDCl3)
3.22 4.82 2.04 2.00 2.11 2.15 6.80 3.28
0.0 9.5 9.0 8.5 8.0 7.5 7.0 6.5 6.0 5.5 5.0 4.5 4.0 3.5 3.0 2.5 2.0 1.5 1.0 0.5 0.0
f1 (ppm)

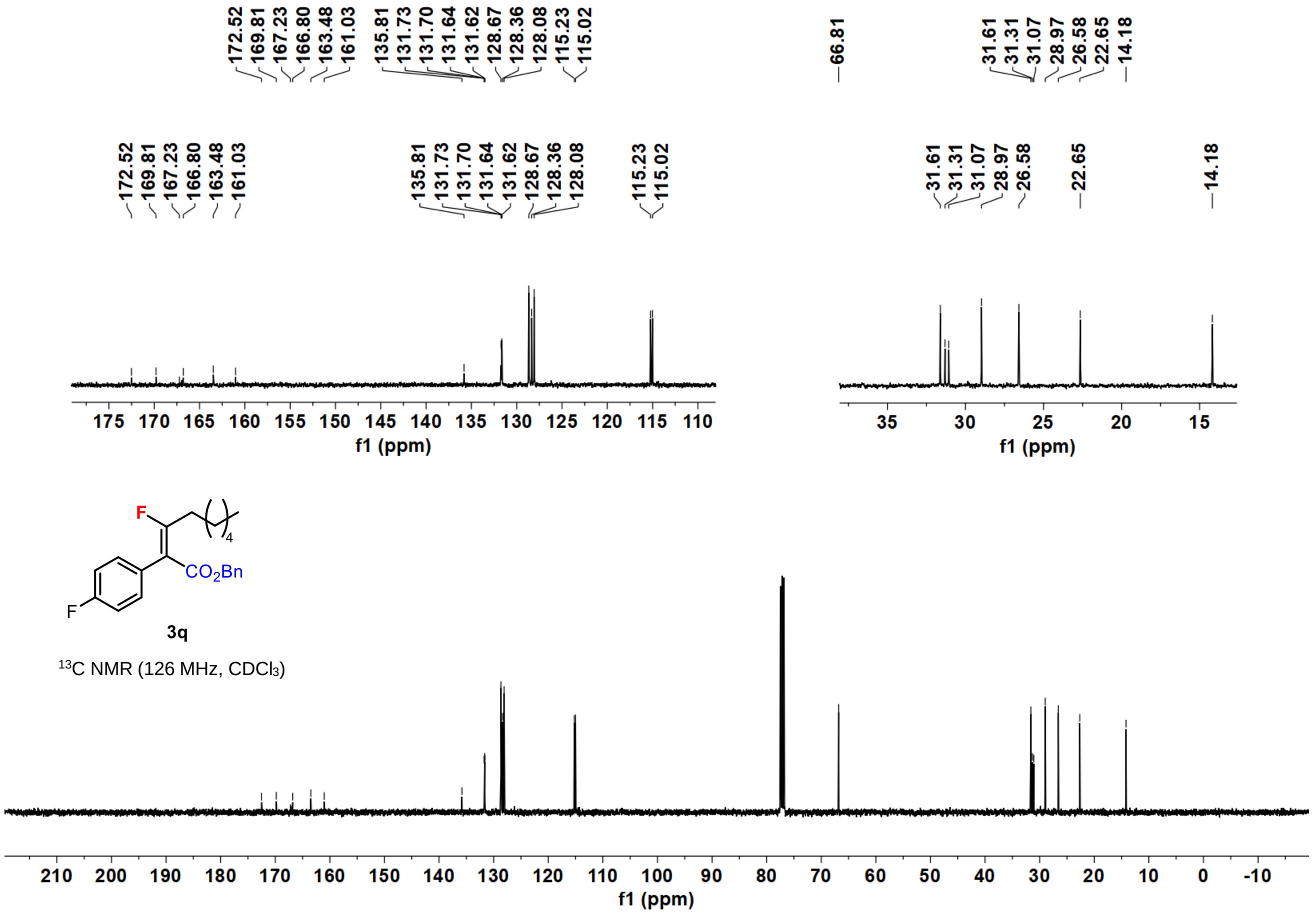
172.52 169.81 167.23 166.80 163.48 161.03 135.81 131.73 131.70 131.64 131.62 128.67 128.36 128.08 115.23 115.02
66.81
31.61 31.31 31.07 28.97 26.58 22.65 14.18
F
4
CO2Bn
F
3q
13C NMR (126 MHz, CDCl3)
f1 (ppm)

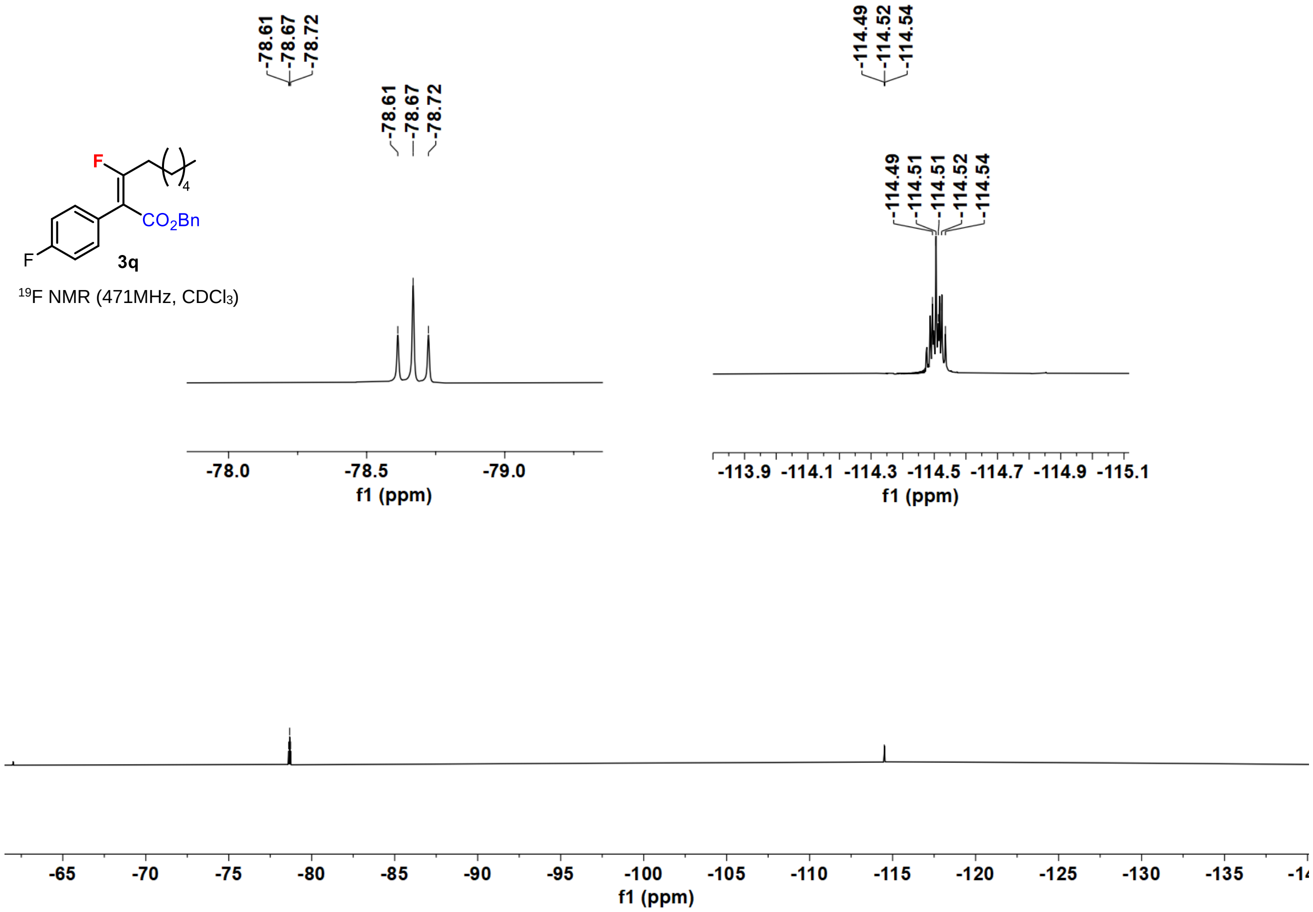
-78.61 -78.67 -78.72
-114.49 -114.52 -114.54
-114.49 -114.51 -114.51 -114.52 -114.54
F
4
CO2Bn
F
3q
19F NMR (471MHz, CDCl3)
f1 (ppm)

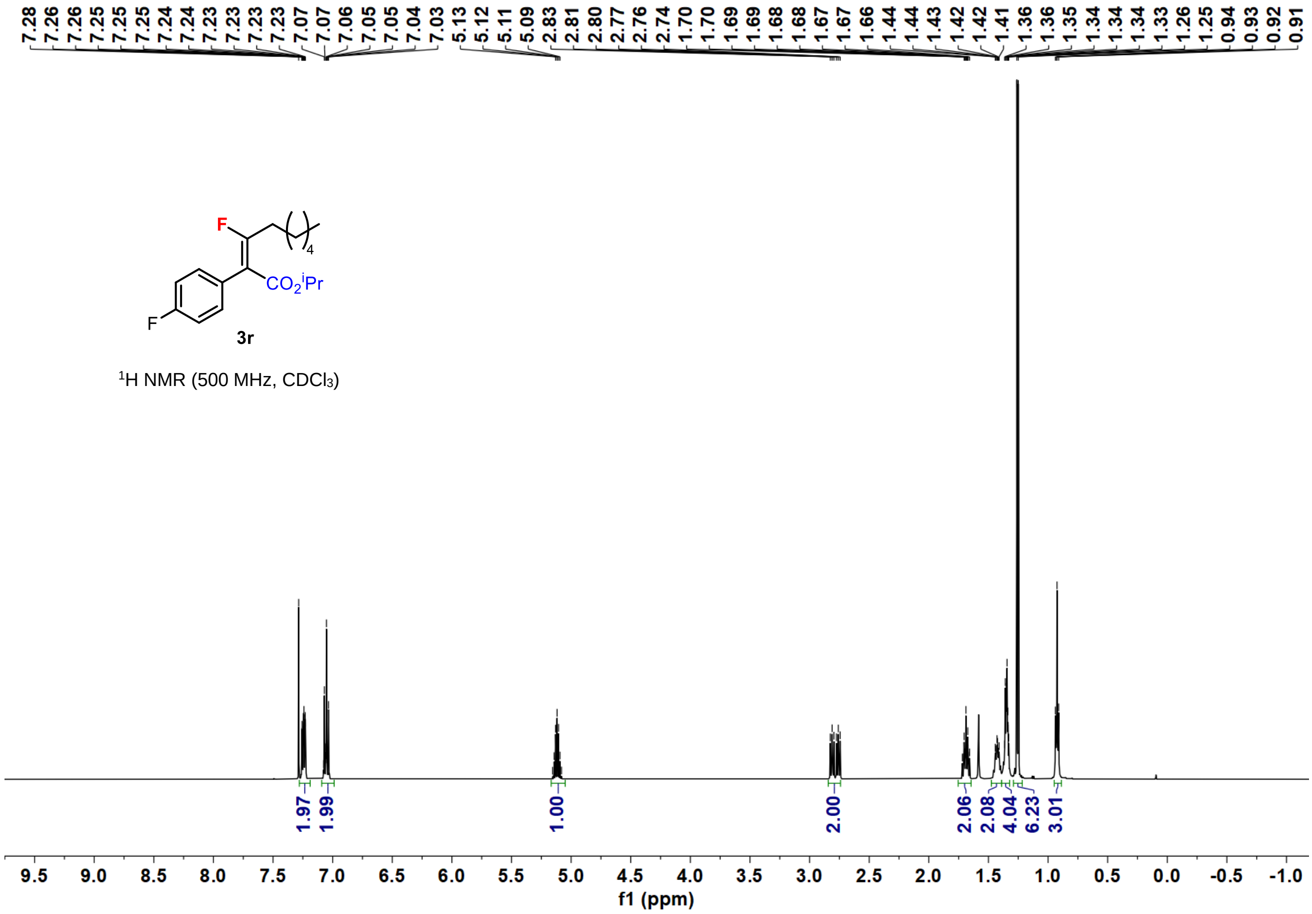

3r

^1H NMR (500 MHz, $CDCl_3$)

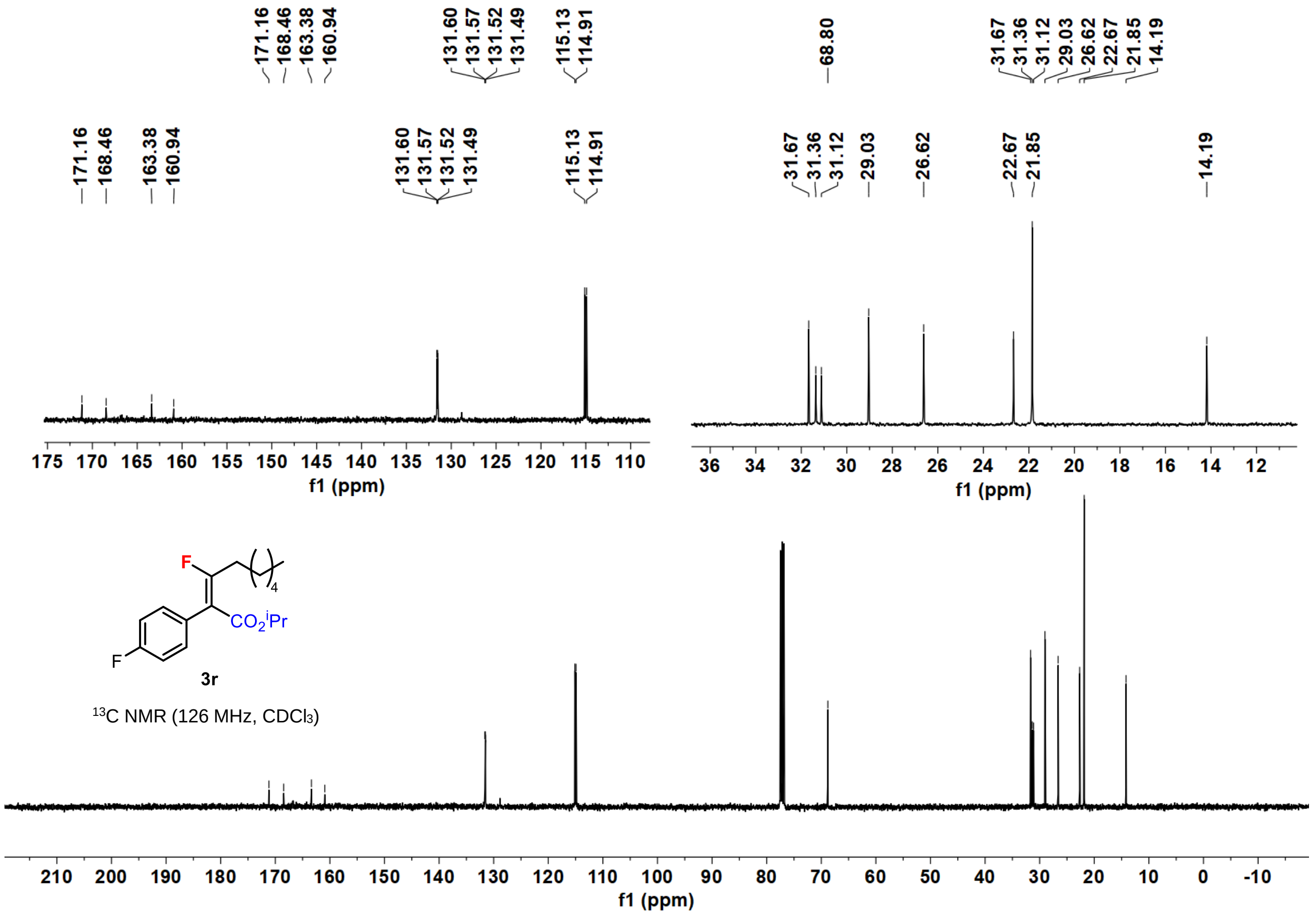

3r

^{13}C NMR (126 MHz, $CDCl_3$)

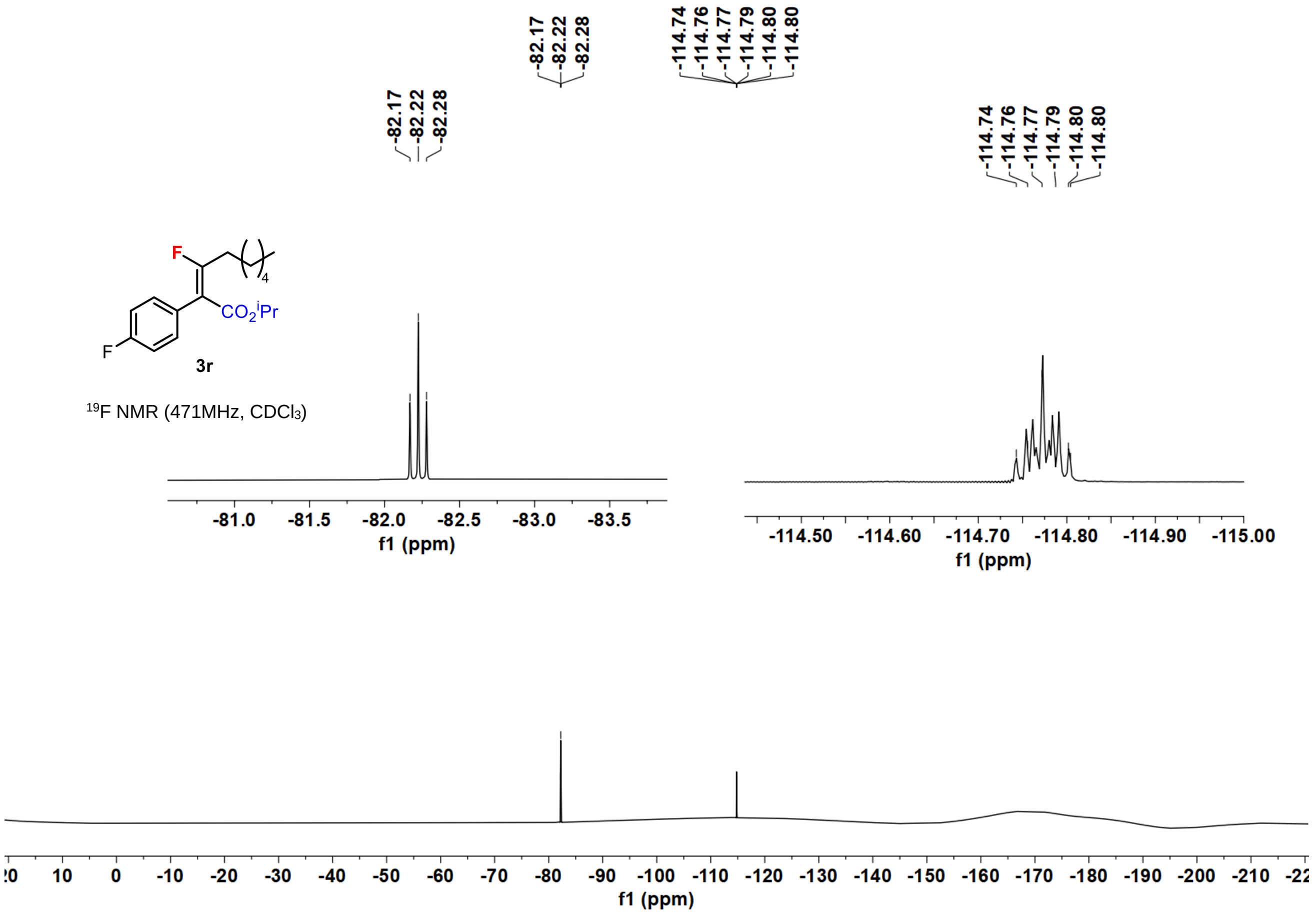
3r
^{19}F NMR (471MHz, CDCl$_3$)
-82.17
-82.22
-82.28
-114.74
-114.76
-114.77
-114.79
-114.80
-114.80
-81.0 -81.5 -82.0 -82.5 -83.0 -83.5
f1 (ppm)
-114.50 -114.60 -114.70 -114.80 -114.90 -115.00
f1 (ppm)
20 10 0 -10 -20 -30 -40 -50 -60 -70 -80 -90 -100 -110 -120 -130 -140 -150 -160 -170 -180 -190 -200 -210 -22
f1 (ppm)

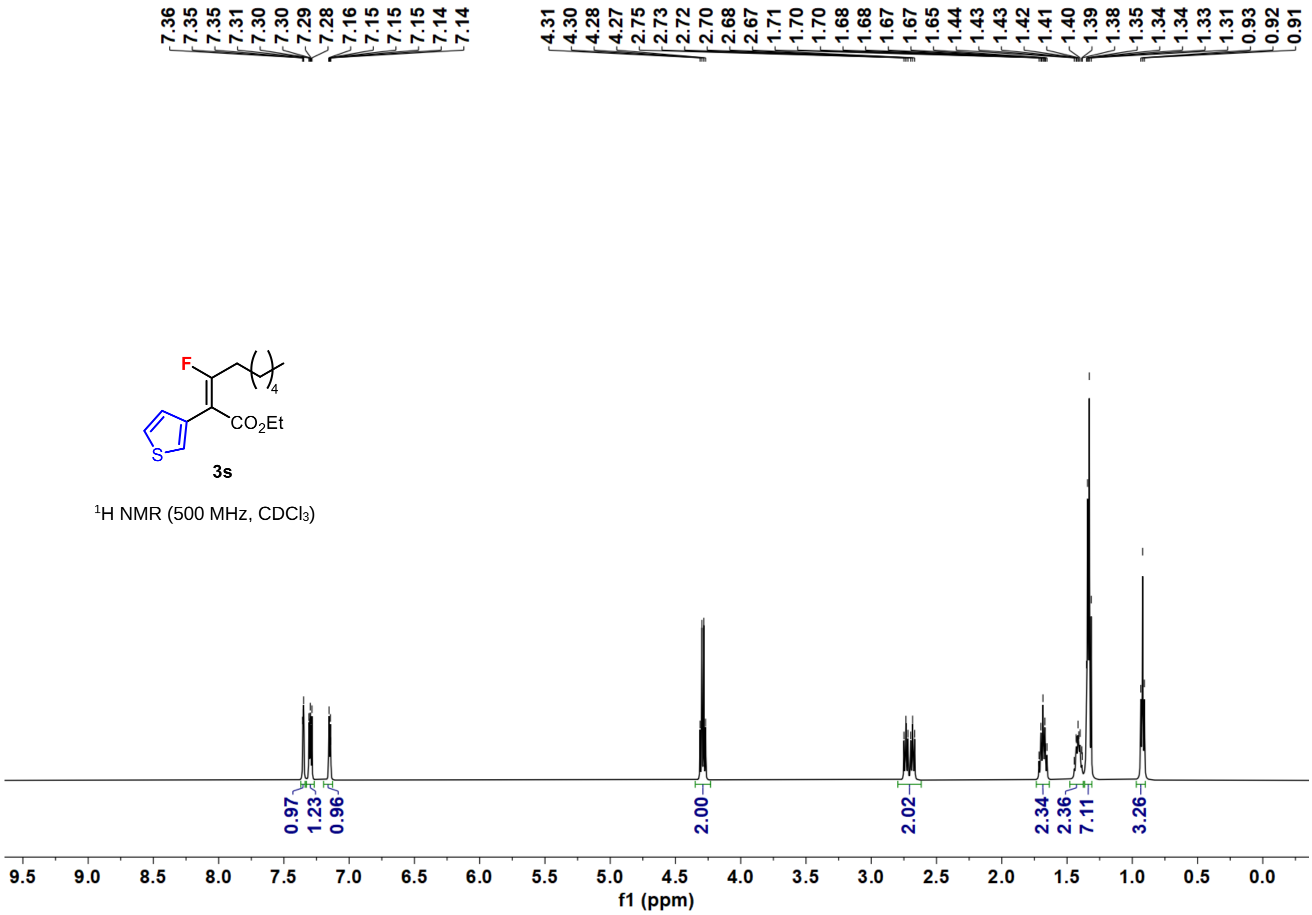
7.36 7.35 7.35 7.31 7.30 7.30 7.29 7.28 7.16 7.15 7.15 7.15 7.14 7.14
4.31 4.30 4.28 4.27 2.75 2.73 2.72 2.70 2.68 2.67 1.71 1.70 1.70 1.68 1.68 1.67 1.67 1.65 1.44 1.43 1.43 1.43 1.42 1.41 1.40 1.39 1.38 1.35 1.34 1.34 1.33 1.31 0.93 0.92 0.91
3s
^{1}H NMR (500 MHz, CDCl$_3$)
0.97
1.23
0.96
2.00
2.02
2.34
2.36
7.11
3.26
9.5 9.0 8.5 8.0 7.5 7.0 6.5 6.0 5.5 5.0 4.5 4.0 3.5 3.0 2.5 2.0 1.5 1.0 0.5 0.0
f1 (ppm)

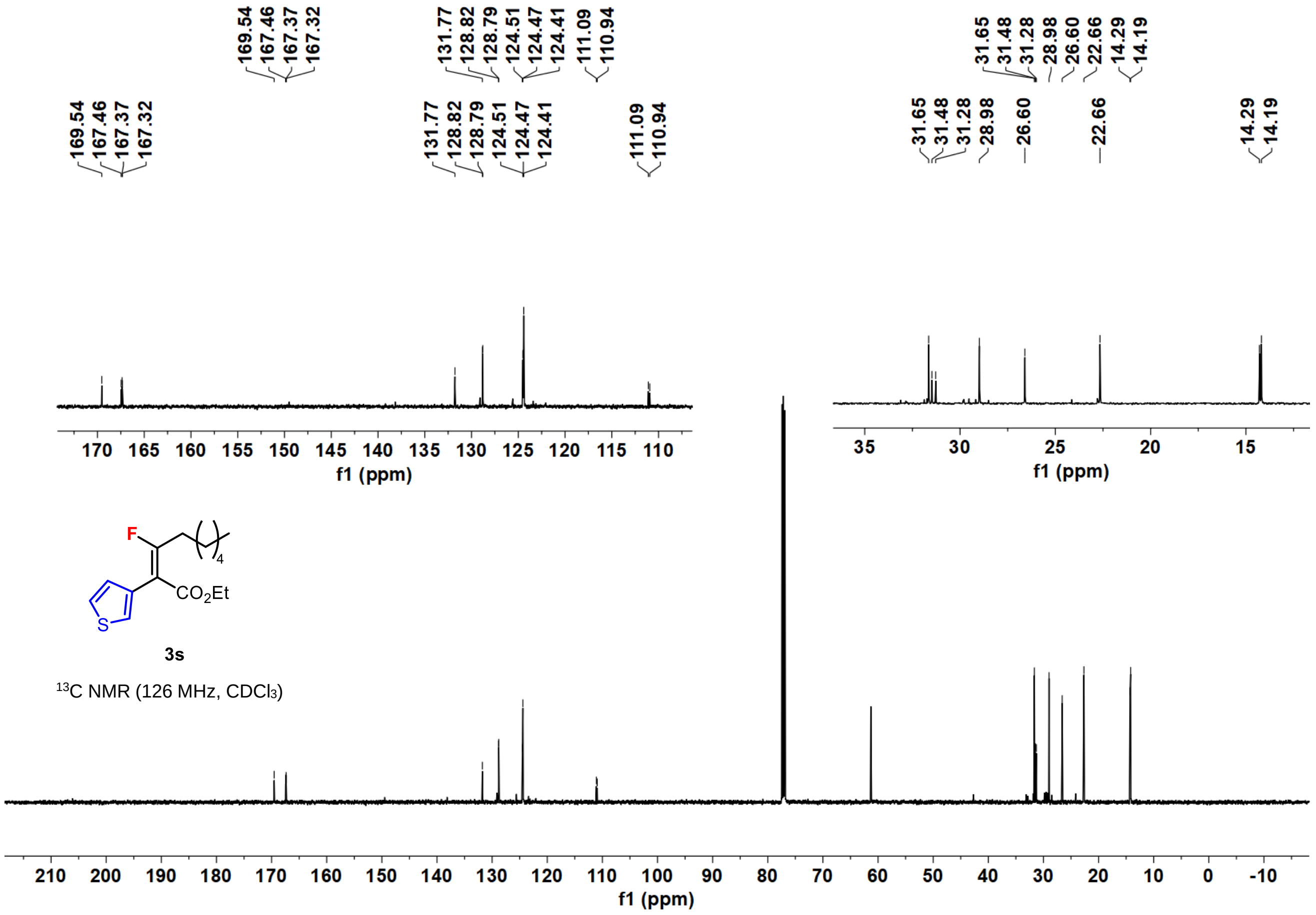
169.54
167.46
167.37
167.32
131.77
128.82
128.79
124.51
124.47
124.41
111.09
110.94
31.65
31.48
31.28
28.98
26.60
22.66
14.29
14.19
f1 (ppm)
F
4
CO2Et
S
3s
13C NMR (126 MHz, CDCl3)

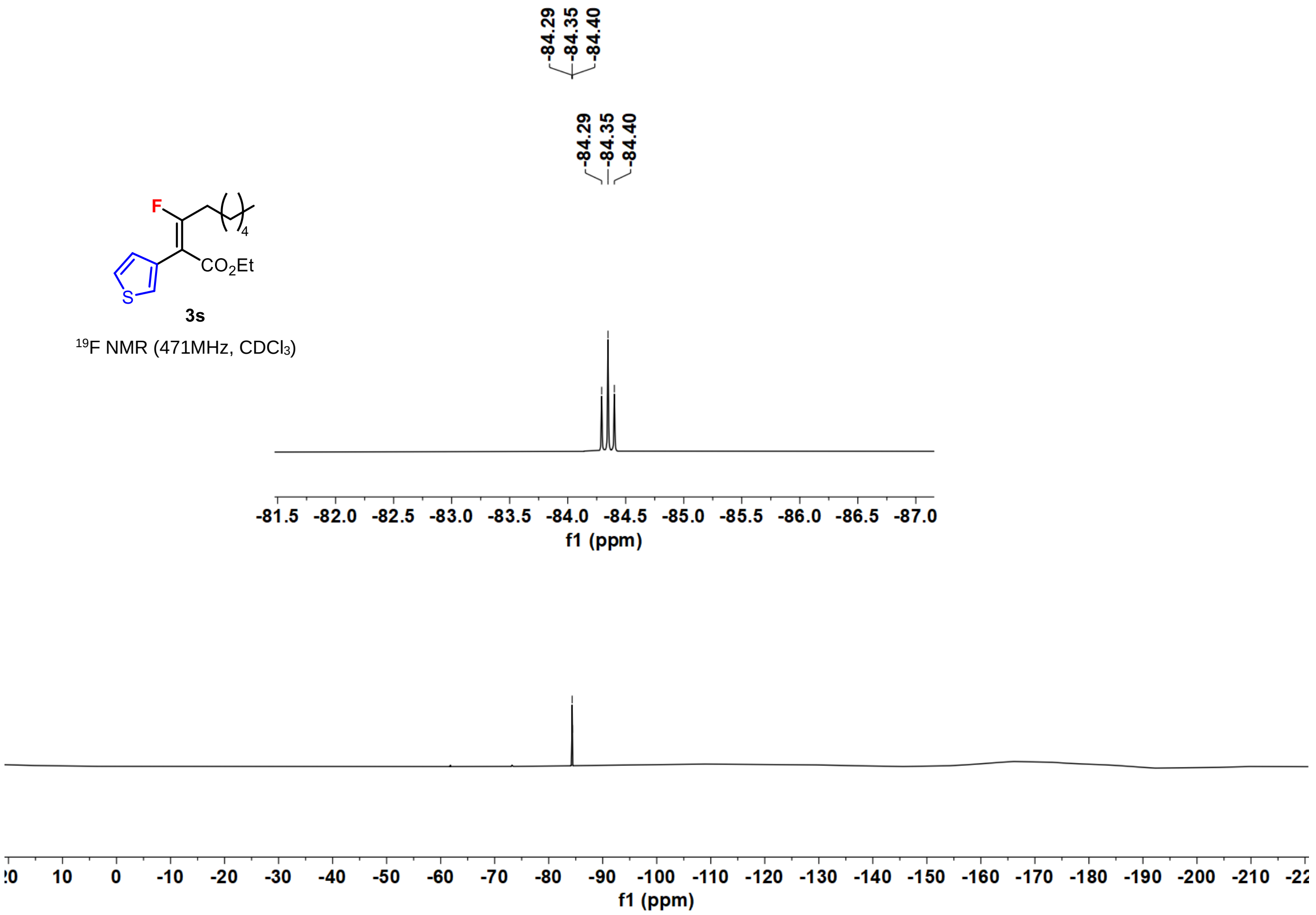
-84.29
-84.35
-84.40
F
4
CO2Et
S
3s
19F NMR (471MHz, CDCl3)
f1 (ppm)

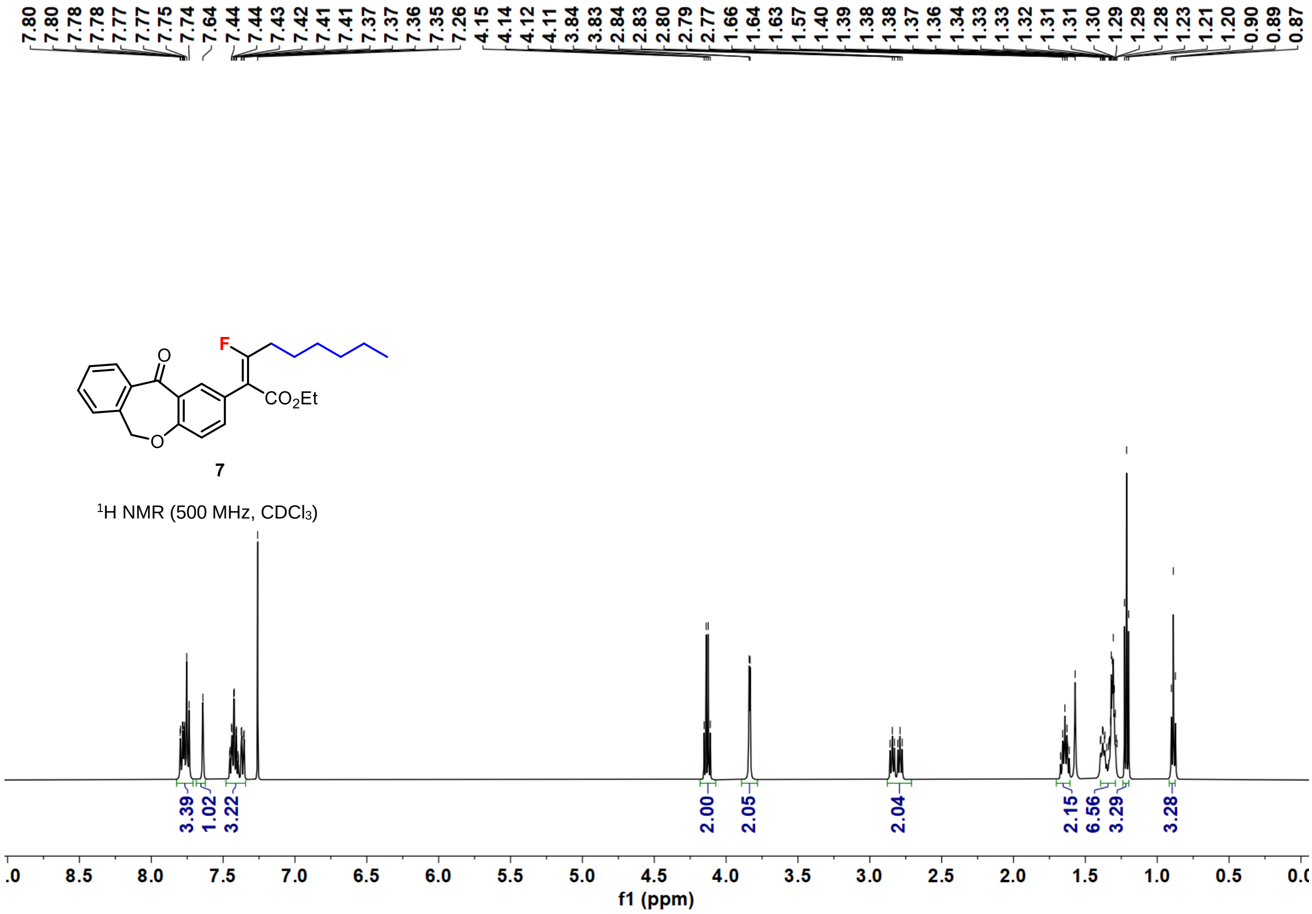

7

^1H NMR (500 MHz, $CDCl_3$)

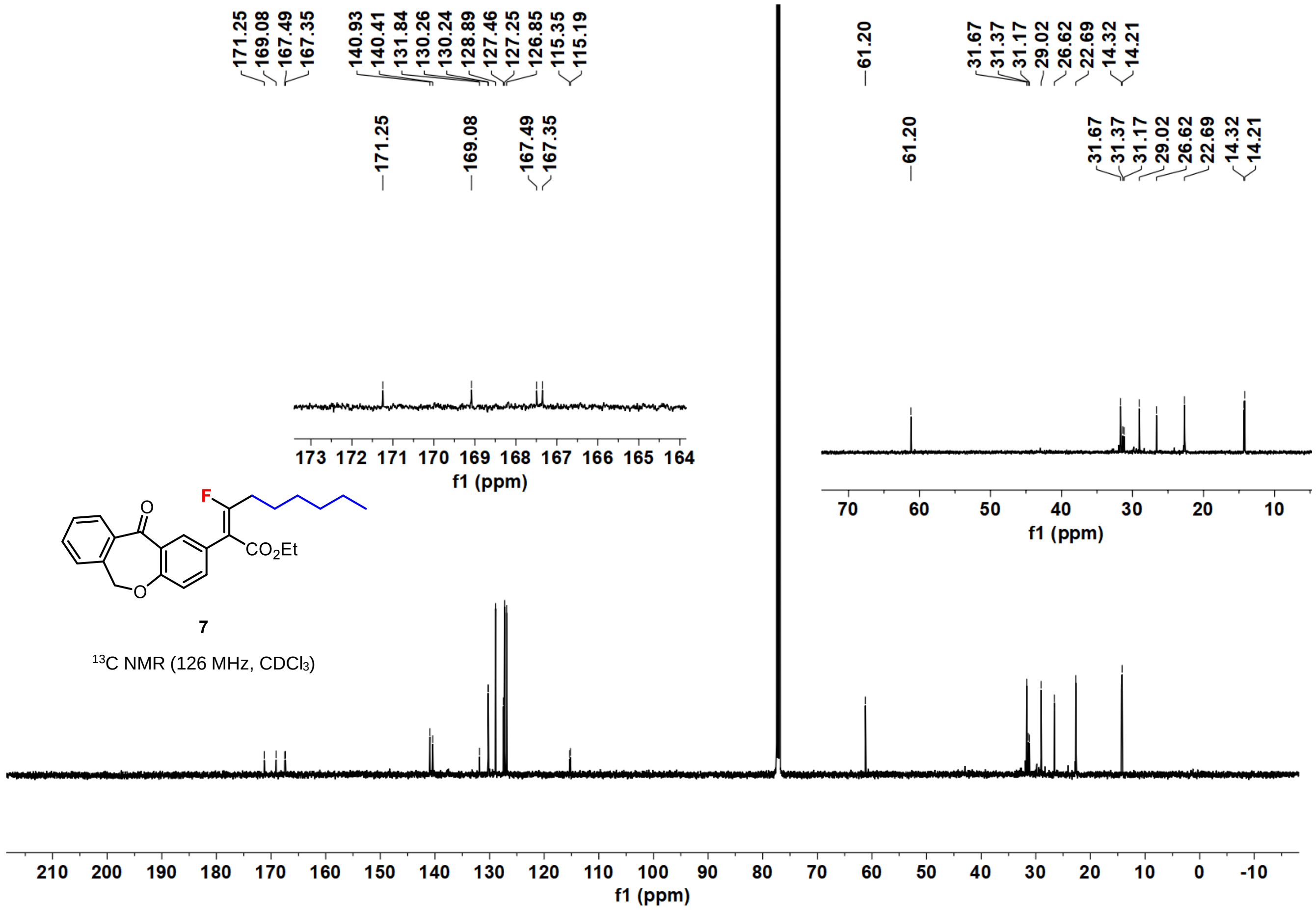

7

^{13}C NMR (126 MHz, $CDCl_3$)

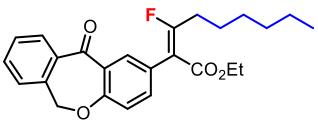

7

^{19}F NMR (471MHz, $CDCl_3$)

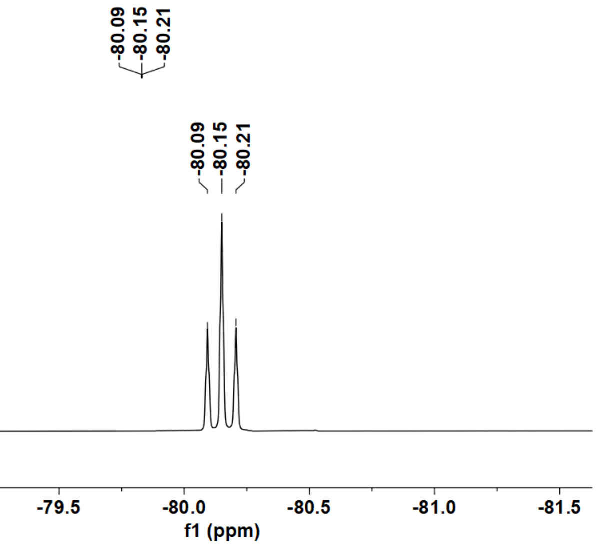

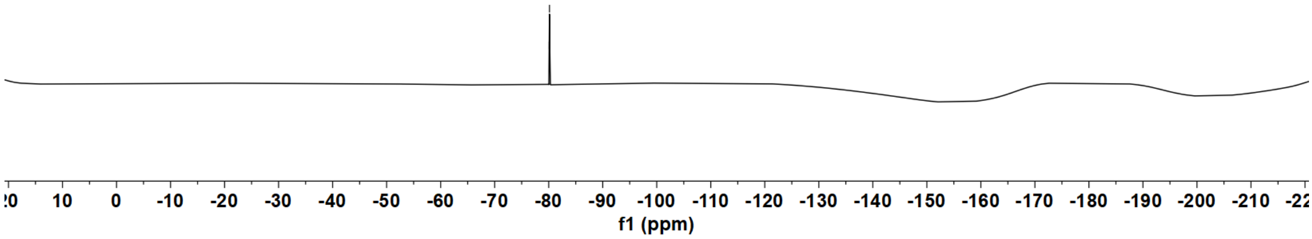

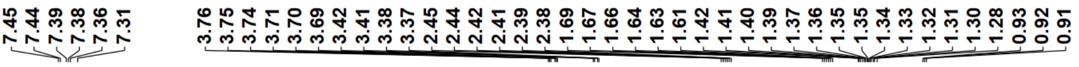

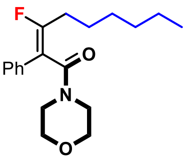

9

^{1}H NMR (500 MHz, $CDCl_3$)

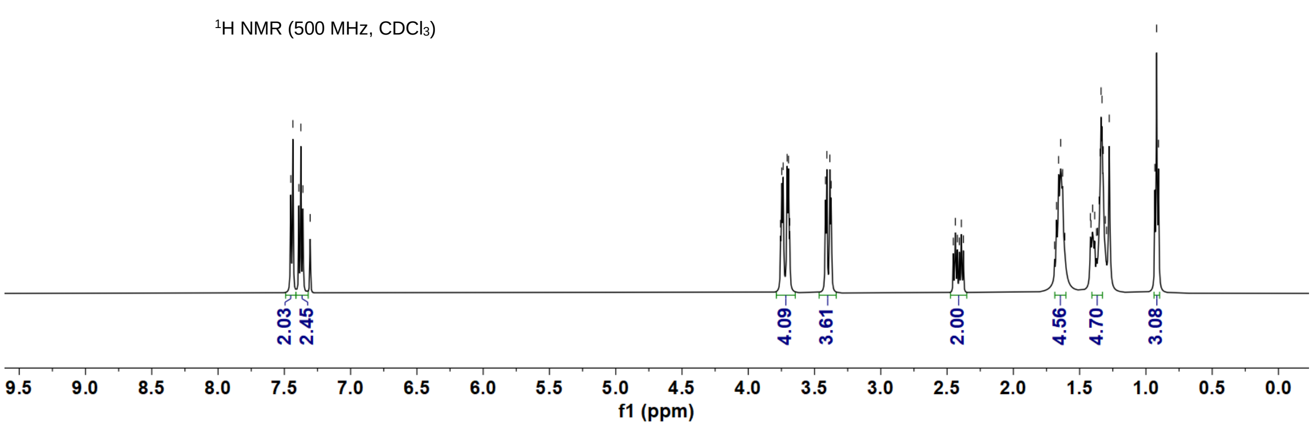

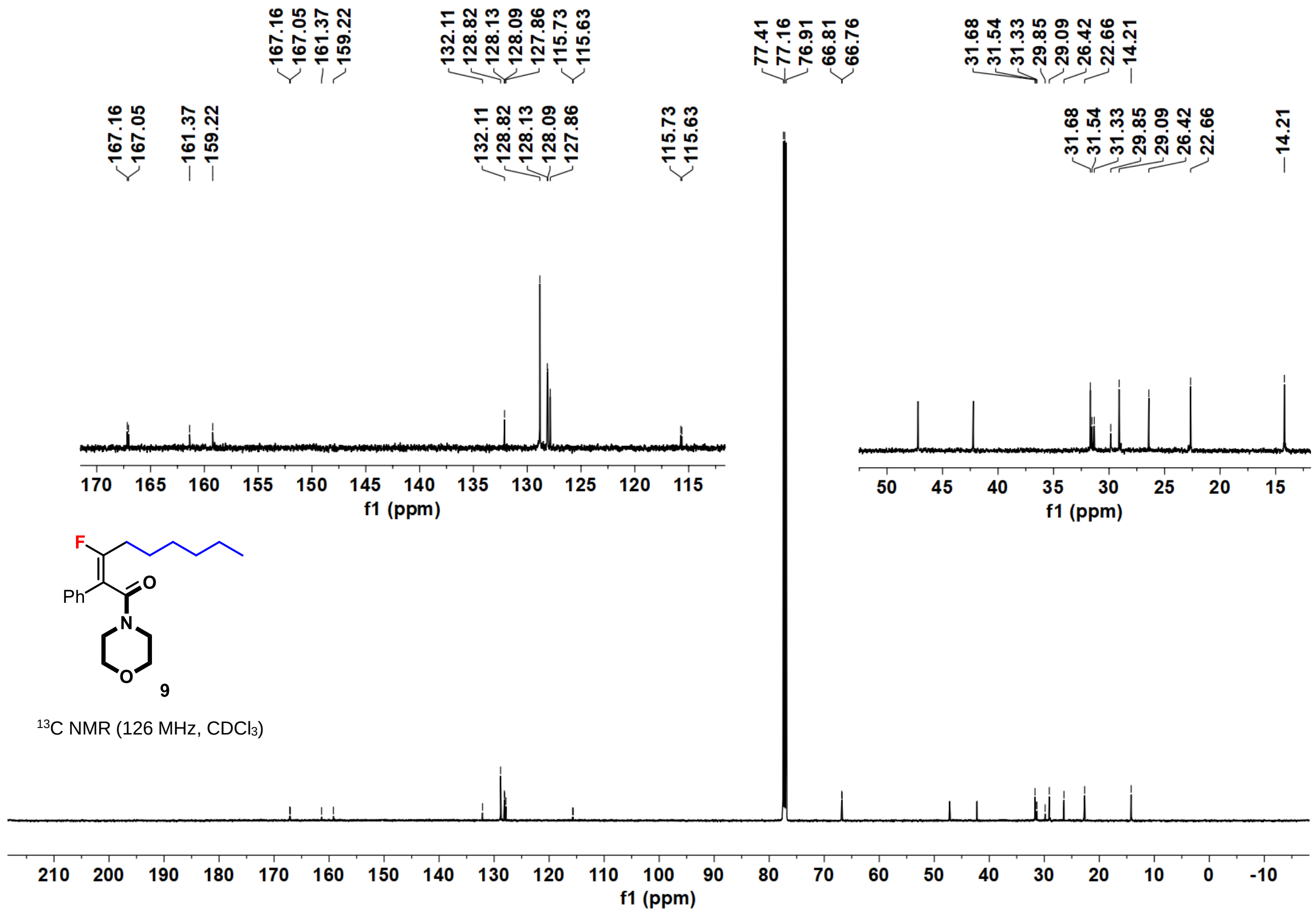
9
^{13}C NMR (126 MHz, CDCl$_3$)
167.16 167.05 161.37 159.22 132.11 128.82 128.13 128.09 127.86 115.73 115.63 77.41 77.16 76.91 66.81 66.76 31.68 31.54 31.33 29.85 29.09 26.42 22.66 14.21
f1 (ppm)

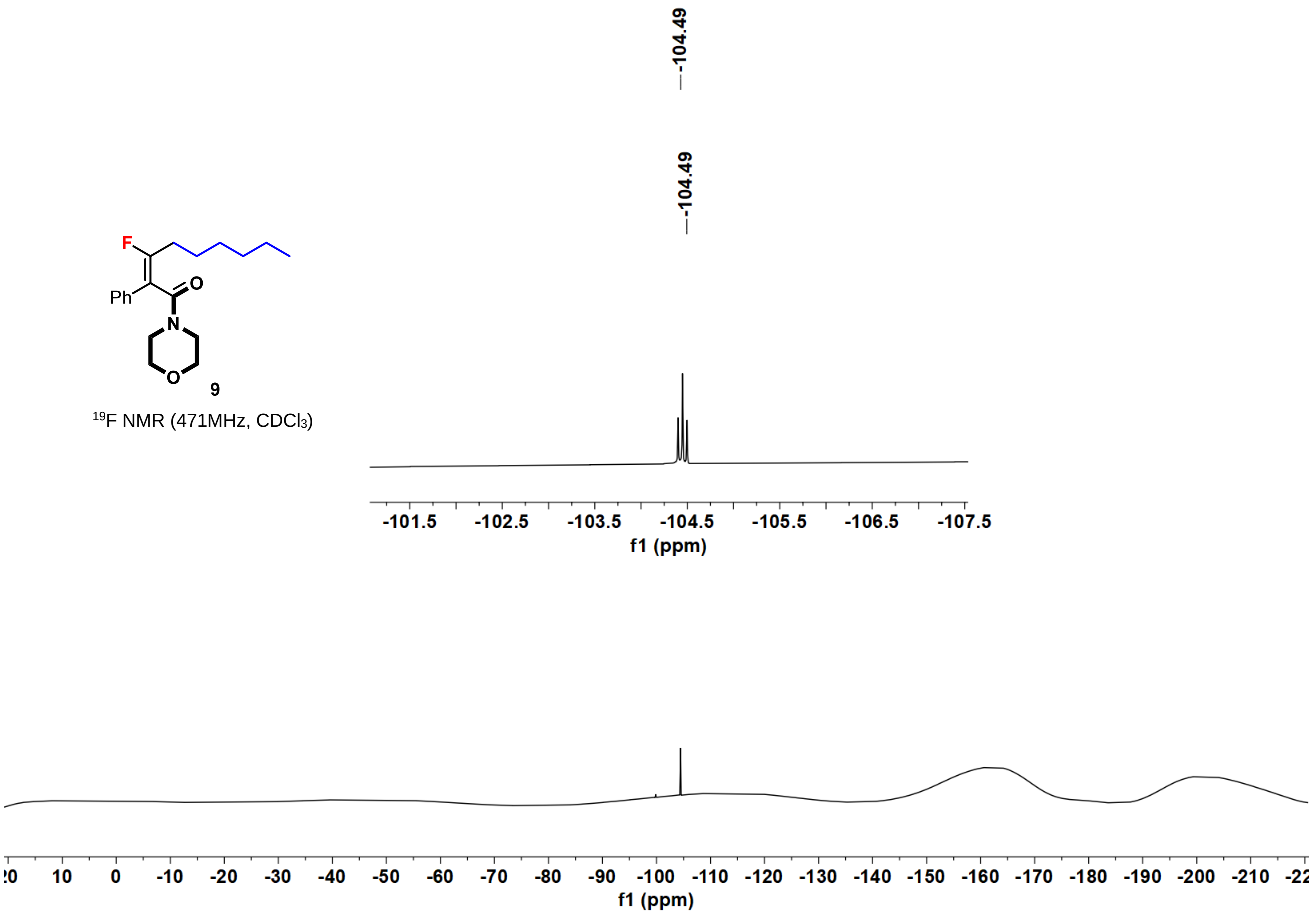
9
^{19}F NMR (471MHz, CDCl$_3$)
-104.49
f1 (ppm)

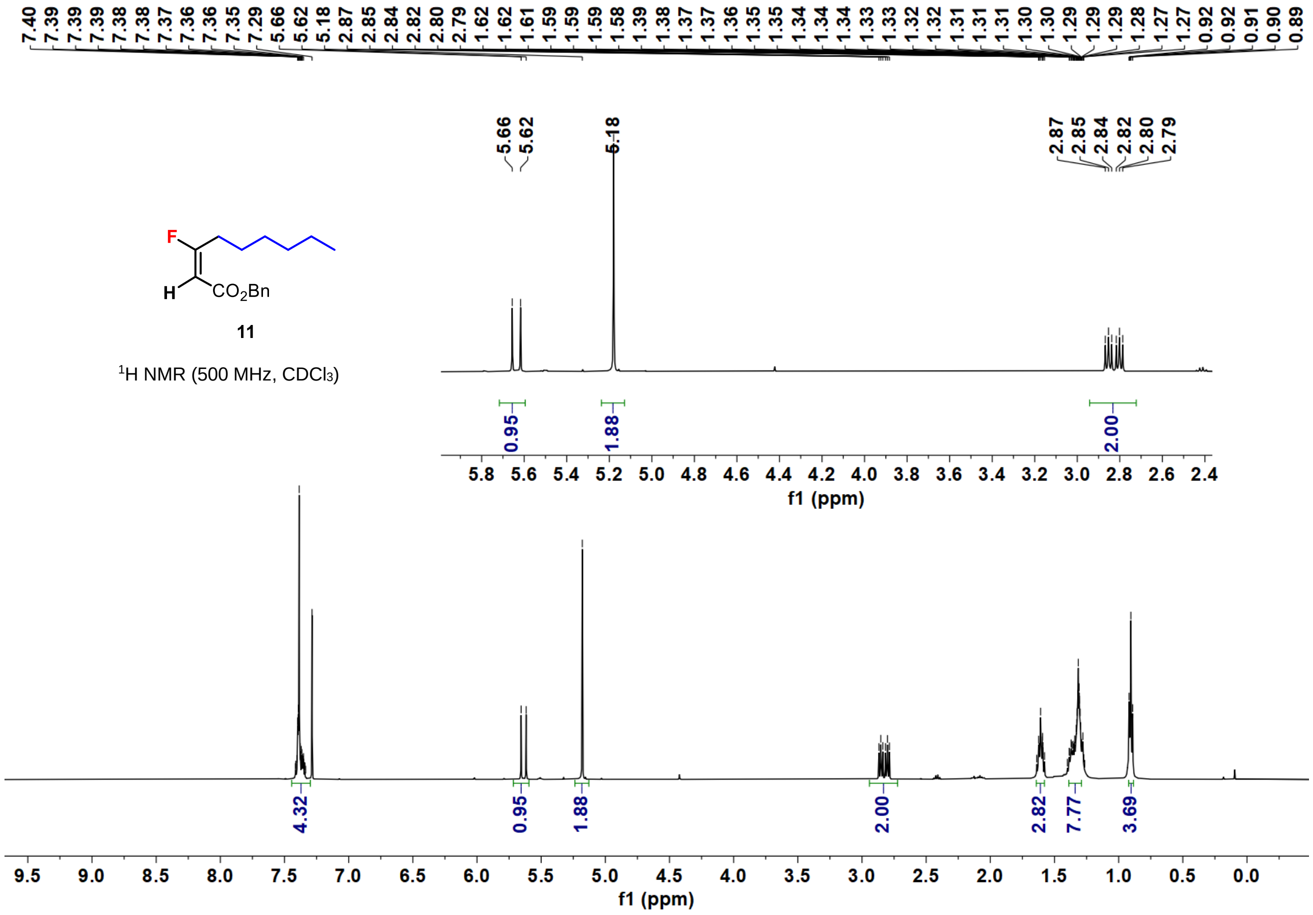
7.40 7.39 7.39 7.39 7.38 7.38 7.37 7.36 7.36 7.35 7.29 5.66 5.62 5.18 2.87 2.85 2.84 2.82 2.80 2.79 1.62 1.62 1.61 1.59 1.59 1.59 1.58 1.39 1.38 1.37 1.37 1.36 1.35 1.35 1.34 1.34 1.34 1.33 1.33 1.32 1.32 1.31 1.31 1.31 1.30 1.30 1.29 1.29 1.29 1.28 1.27 1.27 0.92 0.92 0.91 0.90 0.89
5.66 5.62 5.18 2.87 2.85 2.84 2.82 2.80 2.79
F
H
CO_2Bn
11
^{1}H NMR (500 MHz, CDCl3)
0.95 1.88 2.00
f1 (ppm)
4.32 0.95 1.88 2.00 2.82 7.77 3.69
f1 (ppm)

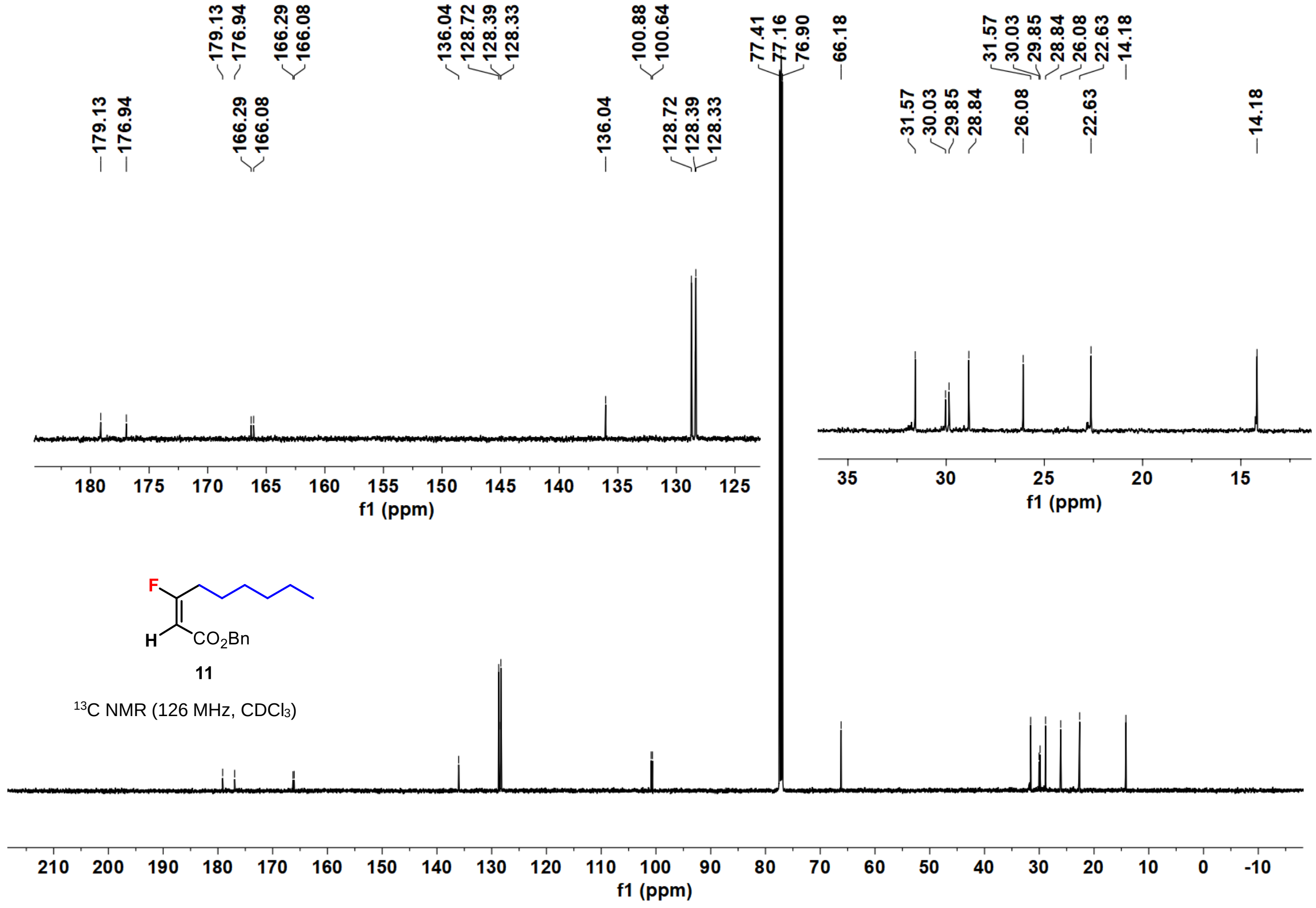
179.13 176.94 166.29 166.08 136.04 128.72 128.39 128.33 100.88 100.64 77.41 77.16 76.90 66.18 31.57 30.03 29.85 28.84 26.08 22.63 14.18
f1 (ppm)
F
H
CO_2Bn
11
^{13}C NMR (126 MHz, CDCl3)
f1 (ppm)

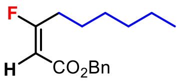

11

^{19}F NMR (471MHz, $CDCl_3$)

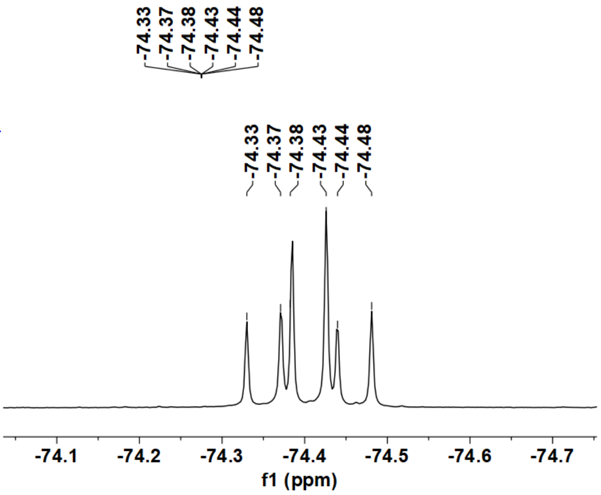

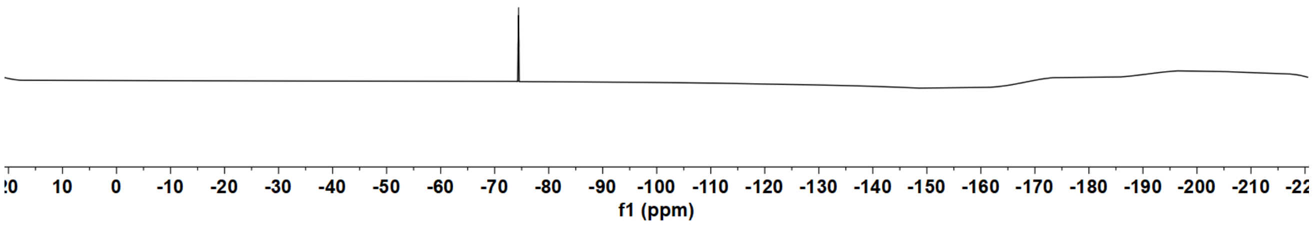

Supplement: Supplementary file 1 — ol3c04037_si_001.pdf [file ol3c04037_si_001.pdf]
